# Supplementary material for: Conserved early steps of stemmadenine biosynthesis[image]
Source: J Biol Chem. 2025 Dec 30;302(2):111120. doi: 10.1016/j.jbc.2025.111120 (PMC12860956; doi:10.1016/j.jbc.2025.111120)
Supplement: Supplementary Material [file mmc1.pdf]

Supporting information

Conserved early steps of stemmadenine biosynthesis

Mohamed O. Kamileen[1], Yoko Nakamura[1,2], Marlen Sigmund[1], Radhika Keshan[1], Veit Gabe[3], Sarah Heinicke[1], Maritta Kunert[1], Benke Hong[4], Ryan Alam[1], Gyumin Kang[1], Lorenzo Caputi[1], Sarah E. O'Connor[1*].

[1]Department of Natural Product Biosynthesis, Max Planck Institute for Chemical Ecology, Jena, Germany.
[2]Research Group Biosynthesis and NMR, Max Planck Institute for Chemical Ecology, Jena, Germany.
[3]Microscopic Imaging Service Group, Max Planck Institute for Chemical Ecology, Jena, Germany.
[4]Zhejiang Key Laboratory of Precise Synthesis of Functional Molecules, Department of Chemistry, School of Science and Research Center for Industries of the Future, Westlake University, Hangzhou, China.

*Corresponding author: oconnor@ice.mpg.de

Contents

- Supporting Table S1-S5 pages 2-7
- Supporting NMR Table S1-S6 pages 8-10
- Supporting Figure S1-S39 pages 11-51
- References page 52

Supporting Tables

Table S1. Nucleotide sequences of biosynthetic genes and transcriptional elements described in this study.

Gene Name	Nucleotide sequence
P19<br>(GoldenBraid 2.0 part ID GB0038)	ATGGAACGAGCTATACAAGGAAACGACGCTAGGGAACAAGCTAACAGTGAACGTTGGGATGGAGGATCAGGAGGTACCACTTCT<br>CCCTTCAAACTTCCTGACGAAAGTCCGAGTTGGACTGAGTGGCGGCTACATAACGATGAGACTAATTCGAATCAAGATAATCCC<br>CTTGGTTTCAAGGAAAGCTGGGGTTTCGGGAAAGTTGTATTTAAGAGATATCTCAGATACGACAGGACGGAAGCTTCACTGCAC<br>AGAGTCCTTGGATCTTGGACGGGAGATTCGGTTAACTATGCAGCATCTCGATTTTTCGGTTTCGACCAGATCGGATGTACCTAT<br>AGTATTCGGTTTCGAGGAGTTAGTATCACCGTTTCTGGAGGCTCTCGAACTCTTCAGCATCTCTGTGAGATGGCAATTCGGTCT<br>AAGCAAGAACTGCTACAGCTTGCCCCAATCGAAGTGGAAAGTAATGTATCAAGAGGATGCCCTGAAGGTACTGAAACCTTCGAA<br>AAAGAAAGCGAGTGA
GFP<br>(Green fluorescent protein)	ATGGTGAGCAAGGGCGAGGAGCTGTTCACCGGGGTGGTGCCCATCCTGGTCGAGCTGGACGGCGACGTAAACGGCCACAAGTTC<br>AGCGTGTCCGGCGAGGGCGAGGGCGACGCCACCTACGGCAAGCTGACCCTGAAGTTCATCTGCACCACCGGCAAGCTGCCCGTG<br>CCCTGGCCCACCCTCGTGACCACCTTCAGCTACGGCGTGCAGTGCTTCAGCCGCTACCCCGACCACATGAAGCAGCACGACTTC<br>TTCAAGTCCGCCATGCCCGAAGGCTACGTCCAGGAGCGCACCATCTTCTTCAAGGACGACGGCAACTACAAGACCCGCGCCGAG<br>GTGAAGTTCGAGGGCGACACCCTGGTGAACCGCATCGAGCTGAAGGGCATCGACTTCAAGGAGGACGGCAACATCCTGGGGCAC<br>AAGCTGGAGTACAACTACAACAGCCACAACGTCTATATCATGGCCGACAAGCAGAAGAACGGCATCAAGGTGAACTTCAAGATC<br>CGCCACAACATCGAGGACGGCAGCGTGCAGCTCGCCGACCACTACCAGCAGAACACCCCCATCGGCGACGGCCCCGTGCTGCTG<br>CCCGACAACCACTACCTGAGCACCCAGTCCGCCCTGAGCAAAGACCCCAACGAGAAGCGCGATCACATGGTCCTGCTGGAGTTC<br>GTGACCGCCGCCGGGATCACTCACGGCATGGACGAGCTGTACAAGTGA
SlUbq10p<br>(*Solanum lycopersicum* Ubiquitin 10 promoter)	GGAGGTCAACTACCCCAATTTAAATTTTATTTGATTAAGATATTTTTATGGACCTACTTTATAATTAAAAATATTTTCTATTTG<br>AAAAGGAAGGACAAAAATCATACAATTTTGGTCCAACTACTCCTCTCTTTTTTTTTTTGGCTTTATAAAAAAGGAAAGTGATTA<br>GTAATAAATAATTAAATAATGAAAAAAGGAGGAAATAAAATTTTCGAATTAAAATGTAAAAGAGAAAAAGGAGAGGGAGTAATC<br>ATTGTTTAACTTTATCTAAAGTACCCCAATTCGATTTTACATGTATATCAAATTATACAAATATTTTATTAAAATATAGATATT<br>GAATAATTTTATTATTCTTGAACATGTAAATAAAAATTATCTATTATTTCAATTTTTATATAAACTATTATTTGAAATCTCAAT<br>TATGATTTTTTAATATCACTTTCTATCCATGATAATTTCAGCTTAAAAAGTTTTGTCAATAATTACATTAATTTTGTTGATGAG<br>GATGACAAGATTTCGGTCATCAATTACATATACACAAATTGAAATAGTAAGCAACTTGATTTTTTTTCTCATAATGATAATGAC<br>AAAGACACGAAAAGACAATTCAATATTCACATTGATTTATTTTTATATGATAATAATTACAATAATAATATTCTTATAAAGAAA<br>GAGATCAATTTTGACTGATCCAAAAATTTATTTATTTTTACTATACCAACGTCACTAATTATATCTAATAATGTAAAACAATTC<br>AATCTTACTTAAATATTAATTTGAAATAAACTATTTTTATAACGAAATTACTAAATTTATCCAATAACAAAAAGGTCTTAAGAA<br>GACATAAATTCTTTTTTTGTAATGCTCAAATAAATTTGAGTAAAAAAGAATGAAATTGAGTGATTTTTTTTTAATCATAAGAAA<br>ATAAATAATTAATTTCAATATAATAAAACAGTAATATAATTTCATAAATGGAATTCAATACTTACCTCTTAGATATAAAAAATA<br>AATATAAAAATAAAGTGTTTCTAATAAACCCGCAATTTAAATAAAATATTTAATATTTTCAATCAAATTTAAATAATTATATTA<br>AAATATCGTAGAAAAAGAGCAATATATAATACAAGAAAGAAGATTTAAGTACAATTATCAACTATTATTATACTCTAATTTTGT<br>TATATTTAATTTCTTACGGTTAAGGTCATGTTCACGATAAACTCAAAATACGCTGTATGAGGACATATTTTAAATTTTAACCAA<br>TAATAAAACTAAGTTATTTTTAGTATATTTTTTTGTTTAACGTGACTTAATTTTTCTTTTCTAGAGGAGCGTGTAAGTGTCAAC<br>CTCATTCTCCTAATTTTCCCAACCACATAAAAAAAAAAATAAAGGTAGCTTTTGCGTGTTGATTTGGTACACTACACGTCATTAT<br>TACACGTGTTTTCGTATGATTGGTTAATCCATGAGGCGGTTTCCTCTAGAGTCGGCCATACCATCTATAAAATAAAGCTTTCTG<br>CAGCTCATTTTTTCATCTTCTATCTGATTTCTATTATAATTTCTCTGAATTGCCTTCAAATTTCTCTTTCAAGGTTAGAATTTT<br>TCTCTATTTTTTGGTTTTTGTTTGTTTAGATTCTGAGTTTAGTTAATCAGGTGCTGTTAAAGCCCTAAATTTTGAGTTTTTTTC<br>GGTTGTTTTGATGGAAAATACCTAACAATTGAGTTTTTTCATGTTGTTTTGTCGGAGAATGCCTACAATTGGAGTTCCTTTCGT<br>TGTTTTGATGAGAAAGCCCCTAATTTGAGTGTTTTTCCGTCGATTTGATTTTAAAGGTTTATATTCGAGTTTTTTTCGTCGGTT<br>TAATGAGAAGGCCTAAAATAGGAGTTTTTCTGGTTGATTTGACTAAAAAAGCCATGGAATTTTGTGTTTTTGATGTCGCTTTGG<br>TTCTCAAGGCCTAAGATCTGAGTTTCTCCGGTTGTTTTGATGAAAAAGCCCTAAAATTGGAGTTTTTATCTTGTGTTTTAGGTT<br>GTTTTAATCCTTATAATTTGAGTTTTTTCGTTGTTCTGATTGTTGTTTTTATGAATTTTGCAG
SlUbq10t (*Solanum lycopersicum* Ubiquitin 10 terminator)	GCTTGTTGTGGTTGTCTGGTTGCGTCTGTTGCCCGTTGTCTGTTGCCCATTGTGGTGGTTGTGTTTGTATGATGGTCGTTAAGG<br>ATCATCAATGTGTTTTCGCTTTTTGTTCCATTCTGTTTCTCATTTGTGAATAATAATGGTATCTTTATGAATATGCAGTTTGTG<br>GTTTCTTTTCTGATTGCAGTTCTGAGCATTTTGTTTTTGCTTCCGTTTACTATACCACTTACAGTTTGCACTAATTTAGTTGAT<br>ATGCGAGCCATCTGATGTTTGATGATTCAAATGGCGTTTATGTAACTCGTACCCGAGTGGATGGAGAAGAGCTCCATTGCCGGT<br>TTGTTTCATGGGTGGCGGAGGGCAACTCCTGGGAAGGAACAAAAGAAAAACCGTGATACGAGTTCATGGGTGAGAGCTCCAGCT<br>TGATCCCTTCTCTGTCGATCAAATTTGAATTTTTGGATCACGGCAGGCTCACAAGATAATCCAAAGTAAAACATAATGAATAGT<br>ACTTCTCAATGATCACTTATTTTTAGCAAATCAGCAATTGTGCATGTCAAATGATTTCGGTGTAAGAGAAAGAGTTGATGAATC<br>AAAATATCTGTAGCTGGATCAAGAATCTGAGGCAGTTGTATGTATCAATGATCTTTCCGCTACAATGATGTTAGCTATCCGAGT<br>CAAATTGTTGTAGAATTGCATACTTCGGCATCACATTCTGGATGACATAATAAATAGGAAGTCTTCAGATCCCTAAAAAATTGA<br>GAGCTAATAACATTAGTCCTAGATGTAACTGGGTGACAACCAAGAAAGAGACATGCAAATACTACTTTTGTTTGAAGGAGCATC<br>CCTGGTTTGACATATTTTTTCTGAATATCAAACTTTGAAACTCTACCTAGTCTAATGTCTAACGACAGATCTTACTGGTTTAAC<br>TGCAGTGATATCTACTATCTTTTGGAATGTTTTCTCCTTCAGTTATACATCAAGTTCCAAGATGCAGGTGTGCTTGATTGATGT<br>ACATGGCTGTGAGAAGTGCATCCTGATGTTCAGATGATGGTTCATTCTAATGTCTTTTCCTTCAATCAGTTTTCTCAGTCTGAC<br>TTAGCTTGTTTCATCTGCATGTTTGAATGTTCGTTTACTCATAGTAATTGCATTTTTGTAGCAGAACATATCATTGGTCATGGT<br>TTCAACTGTGCGCGAGTCTTATGCTTATTCAAACTAGGAAAGCCTCCGTCTAGAGGGTACACGAGTTGTTGCTCTGTGTGCGTC<br>AGTCCATAGTATTAATCTTGCTAGTTGTAGTATATTGTTTATGTGGACTCGGAATTCATCATATGCTCCTTCTTTGCATCAAGT<br>AAGGCAAGGTAATGTATAGAAGCTTTTTAACTCTTTCATGGAAGCTGGCCTTTGCCAGCATACCATCCAGAAGATATCAACCCT<br>GCATCTTGGCTGCCG
TiSGD<br>*T. iboga*<br>Strictosidine β-d-glucosidase<br>(This study)<br>(PX547877.1)	ATGCATTCGTATTCAACAACTATGGAAACTACTCATAGCCCTCTTGTTGCTGCTATTGCTCCAAGATCAACTGCAGTAGCAGAC<br>ACGAAGAATTCTAACGGCACTAGACCAGCGAGCAAGGTCGTCCATCGTCGAGAATTCCCTGAAGATTTTATCTTTGGTGCCGGA<br>GGATCCGCTTATCAGTGTGAGGGAGCAGCTAATGAAGGTAATCGAGGCCCAAGTATATGGGATACTTTCACTCAGCAAACACCA<br>GGCAAAATAGCTGACAGGTCAAATGGAGACAAAGCCATCAATTCTTACCATATGTACAAGGAAGATGTTAAGATTATGAAGCAG<br>ACAGGCCTGGAAGCATATAGATTCTCAATTTCCTGGTCCAGAGTATTGCCAGGTGGAAGATTATCAGCTGGAGTTAATAAGGAG<br>GGAGTCAAGTTCTACCATGACTTTATAGATGAGCTTCTAGCCAATGGCATCAAACCCTTTGCAACTCTCTTCCACTGGGATGCT<br>CCCCAAGCCCTTGAAGACGAGTATGGTGGCTTCTTGAGTTCCCGGATTGTGGACGATTTTTGTGAGTATGCAGAATTTTGTTTT<br>TGGGAATTTGGTGACAAAGTGAAAAATTGGACAACATTCAATGAGCCTCACACCTATAGTGTCAACGGCTATACCATTGGTGAA<br>TTTGCACCAGGGAGAGGTGGATATGACAAAGGTGATCCAGGCACAGAACCCTATTTAGTGTCACACAATATCCTTCTTGCTCAC<br>AAAGCAGCTGTGGAAATATATAGGAAGAAATTTCAGGAATGTCAAGAAGGTGAAATTGGATTTGTAGTTAATGCAACTTGGATG<br>GAGCCTCTCAATCACAACAAGGCTGACATTGAAGCTCAGAAAAGAGCTCTTGATTTTATGCTTGGATGGTTTATGGAGCCATTA<br>ACAACCGGTGACTACCCAAAATCCATGAGAAACCTAGTTGGAGGACGTCTTCCAACATTTTCACCTGAAGAATCCGAAGGATTA<br>AAAGGCTGCTATGATTTTATTGGAATCAATTATTATACTGCTACTTACGTGACTGATGCAGTTAAATCTACCAATGAACGTTTA<br>GATTACAATACTGATGGTCAATATGTCACTACTTTTGACCGGGATAATGTACCCATCGGTCCAGTGTTGTATGGAGGGTGGCAG<br>CATGTTGTTCCAGTTGGACTCCACAAACTTTTGGTTTACACAAAGGACACATACCATGTTCCAGTCCTTTACGTCACGGAAAAT<br>GGTATGGTTGAAGAAAACAAAACCAGCATGCTGCTTCCAGAAGCTCGTCACGACACCAATAGGGTAGATTTTCACCGAGAGCAT<br>ATTTCAAGCGTGCGAGATGCTATTGATGATGGTGTGAATGTAAAAGGTTACTTTGCCTGGTCATTCTTCGACAACTTTGAGTGG<br>AATTTGGGTTTTACATGTCGTTATGGAATTATTCATGTTGATTTCGAAAGTTTTGCAAGATATCCGAAGGATTCAGCCATATGG<br>TACAAGAATTTCATTTTTGGAAAATCTCACACTTTGCCGGTTAAAAGACCTCGTGACGAAGATCGAGAAGCTGAGTTAGTCAAA<br>AGGCAAAAATCCCAAGCAGGCACAAATAATATGGTCTTTTGA

TiGS<br>*T. iboga*<br>Geissoschizine synthase<br>(This study)<br>(PX547878.1)	ATGGCTGCAGAAACAGCCCAACTCGACCTCTCAGTGAAAGCTATTGGATGGGGTGCTACTGATTCATCTGGGGTTCTTCAGCCC<br>ATCAAGTTCTGCAGAAGGACCCCGGGTGAGCGCGATGTGAAGATAAGAGTGTTGTATTGTGGTCTCTGCAATTTCGATATGGAA<br>ATGGTCAGGAACAAATGGGGTTTCACTAGATATCCTTACGTATTTGGACATGAGACTGCGGGTGAGGTGGTAGAAGTAGGCAGC<br>AAGGTGCAGAGATTCAAGGTTGGGGACAAAGTGGCTGTGGGGTGCATGGTTGGATCTTGCGGCAAATGTTACAATTGTGAAAAT<br>GGTATGGAAAATTACTGCCCAGAGCCCAACATGGCTGATGGCTCTGTTTACCGTGAACAAGGAGAGCGTTCATATGGTGGTTGT<br>TCCAATGTCATGGTTGTTGATGAGAAATTCGTTCTCCGTTGGCCCGAGAACTTCCCACAGGACAAAGGAGTTCCTCTCCTGTGT<br>GCTGGGGTCGTTGTTTATAGTCCATTAAAACATTTTGGACTCGACAAACCTGGAATGCACATTGGTGTTTTTGGCCTTGGAGGT<br>CTTGGCTCTGTTGCTGTCAAGTTTATTAAGGCTTTTGGTTCTAAGGCAACTGTGATCAGTACATCAAGGCGTAAAGAGAAGGAA<br>GCCATCGAAGAGCATGGTGCAGATGCATTTGTTGTAAACAATGAGACTGAGCAATTGGAGGCCTTAGCAGGTACAATGGATGCC<br>GTTGTTGACACTACCCCTGGTGGACTCACTCCCATGTCACTCATGCTCAATTTGCTAAAGTTTGACGGAACAGTGATTATGGTT<br>GGTGCACCAGAAAGAGTATTTGACCTCCCAGTGCCACCGCTCATTCTTGGAAGGAAAAAGGTAGTTGGGAGCTCCACTGGAGGT<br>CTGAGGGAGTACCAAGAGGTGCTTGATTTTTCAGCCAAACACAACATTCTGTGTGATACTGAGGTCATCTCGATTGACTATCTC<br>AGCACTGCCATGGAGCGCGTCAGGAATCTGGATGTCAAATACCGATTCTCAATTGACATTGGCAATACATTGAAATATGATGAA<br>TGA
TiSBE<br>*T. iboga*<br>Sarpagan bridge enzyme<br>(This study)<br>(PX547883.1)	ATGGAGATGATGAACTTCTCTTTCACCTCCCCAGTTTTCCTTCTCCTCTCCTTCTTTCTTCTCATGTTACTCAAACAACTAAGA<br>GGAAACAAAGGTGACAAGGGCAAAAAGTTGCCACCAGGTCCAAAAAAACTTCCCATTGTCGGAAATTTGCACCAGATGCTTGGA<br>TCACTGCCTCATCGTGTTCTCAAGAAATTGGCAGATCAATATGGACCGGTTATGCACTTACAGATCGGTGAACTTTCAGCAGTA<br>ATCATATCATCTGCACAAAAAGCAAAACAAGTCTTACAAACTCATGGCATCGCCGTTGCAGATCGGCCCCAAACTACTGTTGCT<br>AAGATCATGTTATACAATAGTCTGGGTGCCACTTTCGCTTCATATGGCGATTATTTGAAGCAATTGCGTCAAATTTATGCATTG<br>GAACTTCTAAGCCCCAAGAGTGTTCGATCGTTCTGGACTATCATGGAAGATGAGCTTTCCATTATGGTTACATCAATCAAGTCT<br>GACGTTGGAGAACCAGTGATTTTGCATGATAAAATGATGACTTATTTGTACGCCACACTTTGCAGAGCTACTGTTGGTAGCGTA<br>TGCAATGGACGAGAGACGTTGATAATGGCTGCAAGAGAAACTTCAGCGCTTTCTGCGGCCATTAGGATTGAAGATCTCTTCCCT<br>TCAGTAAAAATACTTCCTGTTGTCAGTGGACTAAAGTCAAGATTAACCAAACTGATGAAACAACTCGATACTGTTCTAGAGAAT<br>ATAATCAGTGAGCGGGAGAAGAACTTATTCTCATCCAACAACAACCAGCCATTGTCGGATGAAGAAGATATGCTGGGCGTTCTC<br>CTGATGTACAAGAACGGAAAGGGAAAAGACTCTAAATTCCGAATCACCAACAATGACATCAAGGCTATCATTTTTGAATTGATC<br>TTGGCTGGAACTCTAAGTTCAGCAGCTATAGTTGAGTGGTGCATGTCTGAGATGATGAAAAACCCTAGAGTCCTGAAGAAGGCA<br>CAGGATGAAGTGAGGCAAGTTCTCAACGGTAAGAAAACAATCAATGGAGATGATGTTACCAAAATGGATTATGTGAACATGGTA<br>GTCAAGGAATCTGTGAGATTACACCCTCCTGCTCCCCTATTGTTTCCAAGAGAATGCAGGGAAGAATTTGAGATTGACGGAATG<br>ACCATACCCAAAAAATCTTGGGTGATTATAAATTATTGGGCAGTGGGAAGAGACCCTGAACTTTGGGACGATGCCAACGAGTTT<br>AAGCCAGAGAGATTCAGCAACAAACCAATTGATTTCTATGGAAGCCATTTTGAGTTGATACCATTTGGGGCAGGAAGGAGGGTT<br>TGTCCAGGCATATTATTTGGAGTGACTAATGTTGAACTTTTGCTTGCAGCGCTTCTTTATCATTTTGACTGGAAACTCCCTGGA<br>AGCATGAAACCTGAAGAATTAGATATGACTGAGTTATTTGGTGCTGGTTGCATAAGGGAAAACCCATTGTGCCTAATTCCCAAC<br>ATCATTTCAGGAAATTAA
TiGO<br>*T. iboga*<br>Geissoschizine oxidase<br>(This study)<br>(PX547879.1)	ATGGAGCTCTCTTTCTCCTCCCCGGCTCTCTACATCCTCTACTTCATGCTTTTCTTTCTTGTAAAGCAACTGTTAAAGCCCAAA<br>AGTCACAAGAAATTGCCACCAGGTCCTAGGACGCTACCCATAATCGGAAACTTGCACCAGCTTATGGGACCATTGCCTCATCGC<br>ACCCTCAAGGAGTTGTCCGATAAACATGGACCTCTCATGCACTTGAAGATGGGTGAACGTTCAGCAATTATCGTATCAGATGCA<br>AGAATGGCAAAAATAGTCCTGCACAATCATGGGCTGGCAGTGGCGGACCGGTCGGTGAATACTGTTGCTAGCATCATGACCTAC<br>AACAGTTTGGGGGTTACATTTGCTCAATACGGCGATTACTTGACAAAGCTGCGTCAGATTTACACTTTGGAACTCCTTAGTCCC<br>AAAAAAGTTCGATCATTTTACAGTTGTTTTGAAGACGAGCTTGATGGTTTTGTGAAATCAATCAAGTCTCAAGTCGGACAACCC<br>ATGGTTTTGTACGAGAAAGCTTCTACTTACTTGTATGCCACAATTTGCAGAACTATTTTTGGAAGCATATGCAAGGAGAGGGAG<br>AAGATGATCAAGATAGTGAAAAGAACATCGTTACTCTCTGGAACCCCTCTCAGGTTGGAGGATCTGTTCCCTTCAATGAGCGTG<br>TTTTGTAGGTTCAGCAAAACACTGAACCAATTGAGGGATCTGTTACAGGAAATGGATGATATTCTCGAGGATATTATCATTGAG<br>CGTGAAAAGGCCGCTGAAATCTCGACAGAGGCCAAGGACGACGAAGATATGCTCAGCGTTCTCTTGAGGCATAAATGGCACAAT<br>CCCAGCGGCGCAAAGTTCAGAATCACAAACGCAGACATCAAAGCCATCATTTTTGAGTTGATCTTAGCTGCAACTCTAAGCGTA<br>GCAGATGTTACAGAATGGGCTATGGTTGAAATACTGAGGGATCCCAAAAGTTTGAAGAAGGTATACGACGAAGTCAGAGAAGTC<br>TGCAAGGAGAAGAAAAGAGTCACCGGATATGACGTTGAGAAATTAGAATATATGCACCTTTGTTTTAAGGAATCTACAAGAATA<br>CACCCTGCTGCTCCTCTCTTAGTCCCCAGAGAATGCAGAGAAGACTTCGAGGTCGACGGATACACTGTTCCAAAAGGAGCATGG<br>GTGTTGACAAACTGCTGGGCTGTCCAGATGGATCCCAAGATCTGGCCGGAGCCTGAGAAGTTTGATCCAGAGCGATACATCCGT<br>GATCCCATGGATTTCTACGGAAGCAACTTTGAGTTGATACCCTTTGGTACGGGAAGGAGAGGATGTCCAGGAATCTTGTTTGGA<br>GTGACCAATGCTGAGCTTCTCTTAGCTGCAATGTTCTATCATTTTGATTGGGAAATTGCAGACGGGAAAAAACCAGAAGAAATA<br>GATCTTACCGAGGACTTTGGTGCTGGTTGCATTATGAAATATCCTTTGAAACTTGTTCCACATCTTGCCAACGAATAG
TiRedox1<br>*T. iboga*<br>Redox enzyme 1<br>(This study)<br>(PX547880.1)	ATGGCTGATCGCGTCAAGACCATCGGATGGGCAGCTCATGACAGTTCCGGGATCCTCTCTCCCTTCGAATTCACCCGAAGGGCA<br>ACTGGTGATGAAGATGTAAGGTTTAAGGTGCTATATTGTGGTATCTGTCACTCCGACCTTCACAATGTCAAGAACGAAATGGGC<br>TTCACTTCATACCCTTGTGTCCCAGGGCATGAAGTTGTAGGTGAAGTGACGGAGGTTGGAAGCAAAGTCAAGAAATTCAAAGCT<br>GGAGACAAAGTTGGGGTAGGATTATTCGTCGATTCGTGTCGCGAATGCGAGCAGTGTAAAAATGATCTAGAGCCCTACTGCCCC<br>AAGTTGAAAATGGCCTATCTGTCTCCTGATGATGATGGAACCATTATTCAGGGAGGATACTCTAATGAGATGGTAGTCAAGGAA<br>CACTATGTACTTCGGTGGCCGGAAACTCTTCCTCTCGATGCTGGTGCACCGCTAGTCGGCGCCGGTAGCACTGTCTATAGTCCA<br>ATGAAGTATTATGGACTTGATAAGCCAGGACAACATTTGGGAGTTGTTGGCCTCGGTGGACTCGGCCACTTAGCTGTGAAATTC<br>GCCAAGGCTTTTGGACTCAAAGTCACCGTGATCAGTACTTCCCCTGGGAAAAAGGAAGAAGCCATCACTCACCTAGGAGCTGAC<br>TCATTTTTGGTCAGCACTGATCAAGAGCAAATGCAGAAAGCAACGAGCACCTTAGACGGTATCATTGACACAGTATCAGCACCT<br>CATGCAGTGATGCCATTGTTTTTCTTATTGAAACCTCATGGGAAGCTTATTGTAGTCGGGGCACCAAATAAGCCACTTGAAATA<br>GATGTTCCATTTCTTCTTATGGGAAGAAAAATGCTGGGAGCTTCCGCTGTCGGGGGAATGAAGGAGACTCAAGAAATGCTTGAT<br>TTTGCAGCACAACACAATATCTCTGCAGATGTTGAAGTTGTGCCAATGGACTACGTGAACAAAGCTATTGAGCGCCTTGAGAAA<br>GGCGATGTTAGGTATCGATTTGTTCTTGATATTGGGAATACAATGGGCGCTGCCTAG
TiRedox2<br>*T. iboga*<br>Redox enzyme 1<br>(This study)<br>(PX547881.1)	ATGGAAAGGGAAGTTCAAATCCCAGAAATCGAGTTGAACTCCGGGCACAAGATGCCACTTGTGGGCTTCGGGACATGTGTGCCA<br>GATCCCATTCCGCCATTGGAGGAACTTGTAGCAAGCTTCTTGGAGGCTATCAAAGTTGGGTATCGCCACTTTGACACCGCATCA<br>AGCTATGGCACAGAGGAGGCCCTTGGTAAAGCTGTGGCTCAAGCAATAGAGAGTGGCTTGGTTAAGGGCAGAGAGGAATTCTTC<br>ATCAGTGATAAATTGTGGTGTGAAGATGCTGATCATGATCTTATCTTGCCAGCCCTCAAGAAAACACTAGGGAATCTGGGGCTT<br>GATTATTTGGATCTTTATATGATACATATGCCAGTAAGAGTGAGGCAGGGTGCTCCAATGTTCAATTATTCAAAAGAAGATTTA<br>CTCCCTTTTGATATACAAGGGACATGGAAGGCCATGGAAGAATGCAGCAAATTGGGCTTGGTCAAGTCTATTGGTGTAAGCAAC<br>TACACTTGTGAAAAACTCGCAAAACTCTTAGAAAATGCCACCATTCCTCCTGCAGTTAATCAGGTGGAGATGAACGTTGTTTGG<br>CAACAGAGAAAACTGCTGCCATTTTGCAAAGAGAAAAACATTCATGTCACCGCATGGTCTCCTCTGTTATCTTATGGTGCCATT<br>TGGGGCAGTAATGCAGTGATGGAGAATCCAGTCCTCGTAGATATTGCAGCTTCCAAGAACAAGATCGTGGTTCAGGTTGCACTA<br>AGATGGATATATGAGCAAGGAGCAAGTTTCATCATGAGGACCTTTAAAAAGGAGAGAATGTTCGAAAATGTTCAGATTTTCGAC<br>TGGGAACTCACTAAAGAAGAATTGGACAAGATTCAACAAATCCCTCAACGTAGGGGCACTTTGGGAGAAGATTTCATCCATCCA<br>GAGGGGCCAATTAAATCAGTGGAGGAACTCTGGGATGGAGATTTGTAA
TiSAT<br>*T. iboga*<br>Stemmadenine O-acetyltransferase<br>(This study)<br>(PX547882.1)	ATGACTTCCCAGATGGATGTAGTATCAGAGGAGCTCATTCAACCATCATCTCCAACACCCGAAAGCTTAAAACACCATAAACTT<br>TCACTCCTAGACCAAGTTTCGTTGACATGTCATATTCCTATTATTTTTTTCTACCCAAATCAGTTGATCGCCTCAAATGTCGAC<br>CGTGCCCAAAGATCTGAGCACCTGAAACAATCTTTGTCCAAAGTGTTAACTAATTTTTATGTTTTAGCAGGAAGAATTAATCTA<br>AATTCTTCCATAGATTGTAATGATTCTGGAGTTCCGTTCATCGAAGCTCGAGTTCAGGCTGAACTCTCACAGGCAATTCAGAAC<br>GTCGCCATAGAGGAATTGAATCAATATCTTCCGATCGAACCTTATCCCGGCGGGCAAAGCGAGGTGAAAAAGGATATTCCCCTT<br>GCTGTGAAAATCAGTTTCTTTGACTGTGGAGGCGCAGCAATTGGTGTCTGCTTGTCACATAAGATAGCTGATATATTGTCCTTG<br>GCCACCTTCCTCAATGCTTGGACTGCCACATGCCGAGGGGAAACCCATGTTGTGCTGCCTAATTTTGACTTGGGATCCCATCAT<br>TTTCCGCCAATGGACAACATCCCGGCACCTGACTGGGTACCGGATGAAAAGATTGTGATGAAAAGATTCATTTTTGATAAAGAA

	AAATTAGCAGCCCTCAAAGCACAAGCTTCCTCAGCCTCGGAGGTGAAGAATCCCAGTCGGATAGAAGTTGTTACGGCTTTTATA<br>TGGAAGCACTTCATTGACGTGACCCGAGCGAAATTGGATACTAAAAACAAGTTTGCCGCAGCTCAGGCAGTGAACTTGAGGACA<br>AGAATGAGTACACCCCTTCCTCAGTCTGCTATGGGGAACATCGCCACACTTGCATTCGCTGTTGCAGAGGAGGACAAGGATTTT<br>CACGATCTTGTTGGTCCATTGAGAACTGGCCTAGGGAAAATTGACAACGAACATGTTAAGGACTTACAAAAGGGAGTGACTTAT<br>TTGGATTTTGAAGCGGAACCACAAGAATTGTCTTCTTTCAGCAGTTGGTGTAGGCTTGGCTTTTATGAAATGGACTTTGGGTGG<br>GGGAAGCCTCTTTCTGTGTGCACTGCAAATTTGCCCATGAAGAATACCGTATATATGATGGATACTAGATCTGGAGATGGAATA<br>GAAGCATGGATTAGCATGGCGGAAGATGAAATGGCTATGCTTCCCACTGAATTCCTTTCACTTGTAGACAGTGATTTTAGCAAG<br>TGA
CrSGD<br>*C. roseus*<br>Strictosidine β-d-glucosidase<br>(AF112888.1)	ATGGGATCTAAAGATGATCAGTCCCTTGTTGTTGCCATTTCTCCAGCTGCTGAACCAAATGGAAATCATTCTGTCCCCATCCCA<br>TTCGCCTACCCCAGTATCCCCATTCAACCTAGAAAGCACAACAAGCCCATCGTTCATCGTCGAGATTTCCCCTCAGATTTCATC<br>TTGGGTGCCGGAGGATCTGCTTATCAGTGTGAGGGTGCATATAATGAAGGCAACCGCGGTCCCAGTATATGGGATACTTTCACA<br>AACCGATATCCAGCCAAAATAGCTGATGGATCTAATGGCAATCAAGCCATCAATTCTTACAATTTGTACAAGGAAGATATCAAG<br>ATTATGAAGCAAACAGGCTTGGAATCATATAGGTTTTCAATTTCATGGTCAAGAGTATTGCCAGGTGGGAATCTATCCGGTGGA<br>GTGAATAAAGATGGTGTCAAGTTCTATCATGACTTTATAGATGAGCTTCTAGCCAATGGCATCAAACCCTTTGCAACTCTCTTC<br>CACTGGGATCTTCCCCAAGCTCTTGAAGACGAGTATGGAGGCTTCTTGAGTGATCGAATTGTGGAAGATTTTACGGAGTATGCA<br>GAATTTTGCTTTTGGGAATTCGGTGACAAAGTAAAATTTTGGACGACTTTCAATGAACCACATACTTATGTTGCAAGTGGATAT<br>GCCACTGGTGAATTTGCACCAGGAAGAGGTGGTGCAGATGGCAAGGGGGAACCTGGCAAAGAACCCTATATAGCGACACATAAT<br>TTACTTCTTTCTCACAAAGCTGCTGTGGAAGTATATAGGAAAAATTTTCAGAAATGTCAAGGAGGTGAAATTGGAATTGTACTT<br>AATTCAATGTGGATGGAGCCTCTCAATGAAACCAAAGAAGATATTGATGCTCGGGAAAGGGGTCTTGATTTCATGCTCGGATGG<br>TTCATAGAGCCATTAACAACGGGTGAATACCCAAAATCCATGAGAGCTCTTGTAGGAAGCCGTCTTCCAGAATTTTCAACAGAA<br>GTTTCCGAAAAATTAACAGGATGCTATGATTTTATCGGAATGAATTATTATACAACTACTTATGTTTCTAATGCAGACAAAATT<br>CCCGATACTCCGGGTTACGAAACAGATGCTCGAATTAATAAGAATATTTTTGTCAAAAAAGTTGATGGGAAGGAAGTGCGCATT<br>GGTGAACCGTGCTATGGGGGATGGCAGCATGTTGTTCCATCTGGACTCTACAATCTCTTGGTTTACACTAAGGAGAAATACCAT<br>GTTCCAGTGATTTATGTCTCAGAATGTGGTGTGGTTGAGGAAAATAGAACCAACATATTACTTACAGAAGGTAAAACCAACATA<br>TTACTTACAGAAGCTCGTCACGATAAACTCAGGGTTGATTTTCTACAAAGTCATCTCGCTAGCGTGCGAGATGCTATTGATGAT<br>GGTGTGAATGTAAAAGGATTCTTTGTTTGGTCATTCTTCGACAACTTCGAATGGAATTTGGGATATATATGCCGTTATGGAATT<br>ATCCATGTTGATTATAAAACTTTTCAAAGATATCCAAAGGATTCTGCCATATGGTACAAGAATTTCATTAGTGAAGGATTTGTT<br>ACGAATACAGCTAAAAAGAGATTCCGAGAAGAAGATAAACTAGTTGAGTTAGTCAAGAAGCAAAAATACTAA
CrGS<br>*C. roseus*<br>Geissoschizine synthase<br>(MF770507.1)	ATGGCCGGAGAAACAACCAAACTCGACCTTTCAGTGAAGGCTGTGGGATGGGGTGCTGCAGATGCATCTGGTGTCCTTCAGCCC<br>ATCAAGTTCTATAGAAGAGTCCCTGGTGAACGGGATGTGAAGATTAGAGTTTTGTACTCTGGTGTTTGCAATTTCGATATGGAA<br>ATGGTCAGAAACAAGTGGGGTTTCACCAGATATCCTTATGTGTTTGGACATGAAACTGCCGGTGAGGTGGTAGAAGTTGGCAGC<br>AAAGTAGAGAAATTCAAGGTTGGAGACAAGGTAGCTGTGGGATGTATGGTCGGATCTTGTGGTCAATGTTATAATTGTCAAAGT<br>GGAATGGAGAATTACTGCCCAGAGCCCAATATGGCTGATGGATCTGTTTACCGTGAGCAAGGGGAACGATCCTATGGGGGTTGT<br>TCAAATGTGATGGTTGTTGATGAAAAGTTCGTCCTCCGATGGCCCGAAAACTTGCCTCAAGATAAAGGGGTTGCTCTCCTTTGT<br>GCTGGGGTTGTTGTTTATAGCCCAATGAAACATTTGGGACTCGATAAGCCAGGAAAGCATATTGGGGTTTTCGGGCTGGGAGGT<br>CTTGGTTCTGTTGCTGTTAAGTTTATTAAGGCTTTTGGTGGTAAGGCTACTGTTATTAGTACATCAAGGCGTAAGGAGAAGGAA<br>GCCATTGAAGAACATGGTGCTGATGCTTTTGTTGTCAACACTGACTCTGAGCAATTGAAGGCTCTGGCAGGTACTATGGATGGT<br>GTTGTGGACACCACCCCAGGTGGCCGCACTCCTATGTCACTTATGCTCAATTTGCTCAAGTTTGACGGCGCGGTTATGCTCGTA<br>GGTGCACCGGAGTCGCTATTTGAGCTCCCTGCGGCACCTCTCATTATGGGAAGGAAAAAGATAATCGGAAGTTCCACTGGAGGT<br>CTCAAAGAGTACCAAGAAATGCTTGATTTCGCAGCCAAACATAACATTGTATGTGATACTGAAGTTATTGGGATTGACTATCTC<br>AGCACTGCTATGGAACGTATCAAGAATTTGGATGTCAAGTACCGTTTTGCGATTGACATTGGAAATACATTGAAATTTGAGGAA<br>TGA
CrSBE<br>*C. roseus*<br>Sarpagan bridge enzyme<br>(PV446798.1)	ATGGATGAGATGATGAACTTCTCCCTCACCTCTCCCATTTCCTTTCTCCTCTCCTTACTTCTTATCCTATTTCTTATCCTATTT<br>ATGATGAAAGGAAACCAAACCCACAATGGCAAAAAATTACCACCGGGTCCCAAAAAACTTCCTATTATAGGAAACCTCCATCAA<br>ATGATAGGTTCACATCCTCATCGTGTTCTAAAAAAATTGGCCGATAAATATGGACCTATTATGCACTTGCAAATGGGCCAACGA<br>TCAGCCGTTGTTATATCATCAGCGGAAAAAGCAAAAGAAATATTGAACATTTATGGTGTTCAAGTTGCTGATAGACCTCAACTC<br>ATCGTATCTAAAATTATGTTGTACAATAGTTTGGGTGTCACTTTTGCTCCTTATGGTGACTACTTAAAGCAATTGCGTCAAATT<br>TATGCCGTAGAACTTCTTAGCCCTAAAACAGTTAGGTCCTTTTGGACAATTATGGAAGATGAAGTTTCCACAATGGTTAACTCA<br>ATTAAATCTGAAATTGGTCAACCAATAATTTTGCATGATAAAATGATGACTTATTTGTATACTATGCTTTGTAGAGTTACATTT<br>GGTGGCGTATGTAATGGACGCGAGACACTAATAATGGCAGCTAAAGAAACGTCAGCGCTTTCTGCTGCTATTAGGATTGAGGAT<br>TTGTTTCCTTCAGTGAAAATACTTCCTTTAATTAGTGGATTAAAGTCAAGATTAAACAATTTGTTGAAAACACTTGATACCATT<br>CTTGAGGATATTATCAGTGTTCGTGAGAAGAAATTATTAACTCAGCCATTGTTGGATGATGAAGATATGTTGGGAGTTCTCCTC<br>AAGTACAAAAATGGAAAGGGAAAAGATACTAAATTTAGAGTCACCAACAACGACATCAAGGCAGTCATTTTTGAAATTATCTTA<br>GCTGGCACCCTAAGTTCATCAGCTATAGTAGAATGGTGTATGTCTGAAATGATAAAAAACTCTGAAAAGTTTAAAAAGGCACAA<br>GATGAAGTTAGGAAAGTTTTAAAGGGTAAAAAAACAATTAGTGGAAGTGATGTTGGTAAAATGGAATATGTTAAAATGGTGGTT<br>AAGGAATCTTTGAGATTACACCCTCCTGCTCCCATATTGTTTCCAAGAGAATGTAGGGAAGAATTTGAGATAGATGGAATGACT<br>ATACCTAAAAAAACTTGGTTGATTGTAAACTACTGGCCAATAGGAAGAGATCCCAGACTTTGGGACGACGCCGACAAGTTTGAG<br>CCGGAGAGGTTCAGTAATAGCAGCATTGATTTCAATGGAAGCCATTTTGAGTTGATACCATTTGGTGCTGGAAGAAGGATTTGT<br>CCTGGAATATTACTTGGAACAACAAATGTTGAGCTTTTACTTGCTACATTTCTCTATTATTTTGATTGGAAACTTCCTCAAGGT<br>ATGAAACCCGAAGAATTAGATATGAATGAGGTATTTGGTGCTGGTTGCATAAGGGAAAGTCCATTGTGTCTCATTCCTAGCATC<br>TCATCAGCAGTTGAAGGAAATTAA
CrGO<br>*C. roseus*<br>Geissoschizine oxidase<br>(JN613015.1)	ATGGAGTTTTCTTTCTCCTCACCAGCTCTCTACATAGTTTATTTCTTGCTTTTCTTTGTGGTAAGGCAATTATTGAAACCCAAA<br>AGTAAGAAAAAATTGCCACCAGGTCCAAGAACACTACCCTTAATTGGAAACCTTCATCAACTCTCGGGACCTTTACCTCATCGT<br>ACCCTAAAAAATTTGTCCGATAAACATGGTCCTTTGATGCACGTGAAAATGGGCGAACGTTCGGCAATTATAGTATCAGATGCA<br>AGAATGGCAAAAATAGTTCTTCATAATAACGGTTTAGCCGTTGCAGATCGGTCAGTAAATACTGTCGCAAGTATTATGACTTAT<br>AATAGTTTGGGTGTTACCTTTGCTCAGTATGGAGATTACTTAACAAAATTACGTCAAATCTATACTTTGGAACTTTTAAGTCAG<br>AAAAAAGTTCGATCTTTCTACAGTTGTTTTGAAGATGAACTCGATACTTTTGTTAAGTCAATTAAGTCTAACGTTGGACAACCT<br>ATGGTTTTGTACGAGAAAGCTTCTGCTTATTTGTATGCTACCATTTGTAGAACTATTTTTGGGAGTGTTTGCAAGGAAAAGGAG<br>AAAATGATTAAGATAGTGAAGAAAACGTCGTTACTGTCTGGAACACCACTTAGATTGGAGGATCTTTTTCCTTCTATGAGCATT<br>TTTTGTAGATTTAGTAAAACTTTGAATCAATTGAGAGGACTTTTGCAAGAAATGGATGATATTTTGGAAGAAATCATAGTTGAA<br>AGGGAAAAGGCTTCTGAAGTCTCAAAAGAGGCAAAGGATGATGAAGATATGCTTAGTGTTCTGTTGAGGCATAAATGGTACAAT<br>CCAAGTGGTGCTAAATTCAGAATCACTAATGCAGACATCAAAGCCATTATCTTTGAGTTGATCTTAGCAGCAACCCTAAGTGTA<br>GCAGATGTTACAGAATGGGCTATGGTTGAAATTCTAAGGGATCCCAAAAGTTTGAAAAAAGTGTACGAAGAAGTACGAGGTATT<br>TGTAAAGAAAAGAAAAGAGTGACAGGATACGACGTTGAAAAAATGGAATTTATGCGTCTTTGTGTGAAGGAATCAACAAGAATA<br>CATCCAGCTGCTCCTCTTTTAGTTCCTAGAGAATGCAGAGAAGATTTTGAAGTAGATGGATACACAGTTCCAAAAGGGGCTTGG<br>GTTATAACAAATTGCTGGGCTGTTCAAATGGATCCTACAGTATGGCCCGAACCAGAGAAGTTTGATCCAGAAAGATATATTCGT<br>AACCCAATGGATTTCTATGGAAGTAACTTTGAATTGATACCATTTGGGACAGGTAGAAGAGGATGTCCTGGTATTTTATATGGA<br>GTGACAAATGCTGAGTTTATGTTGGCTGCTATGTTTTATCATTTTGATTGGGAAATAGCAGATGGTAAAAAACCAGAAGAAATT<br>GATTTGACTGAGGACTTTGGTGCTGGTTGCATTATGAAGTATCCTTTGAAATTGGTTCCTCATCTTGTTAACGATTAG
CrRedox1<br>*C. roseus*<br>Redox enzyme 1<br>(MF770509.1)	ATGGCTGATCGCGTGAAGACCGTAGGATGGGCAGCTCACGACAGCTCCGGCTTCCTCTCTCCCTTCCAATTCACTCGAAGGGCA<br>ACAGGTGAAGAAGATGTGAGGTTGAAGGTGTTGTACTGTGGTGTCTGTCACTCAGACCTTCATAACATCAAGAACGAAATGGGA<br>TTCACCTCCTACCCTTGTGTCCCCGGGCATGAGGTTGTGGGGGAAGTGACGGAAGTGGGGAACAAAGTAAAGAAATTCATAATT<br>GGTGATAAAGTTGGGGTTGGATTATTCGTTGACTCATGTGGCGAATGCGAACAATGTGTGAATGATGTAGAAACCTATTGTCCC<br>AAATTGAAAATGGCTTATTTATCCATTGATGATGATGGAACTGTGATTCAAGGAGGGTACTCAAAGGAAATGGTCATCAAAGAA

	CGCTACGTTTTCCGGTGGCCGGAAAATCTTCCTCTACCCGCCGGTACACCGCTTCTGGGTGCCGGTAGTACAGTTTATAGTCCA<br>ATGAAATACTATGGACTTGATAAGTCAGGACAACATCTAGGAGTTGTTGGCCTTGGTGGACTTGGTCATTTAGCTGTTAAATTT<br>GCAAAGGCTTTTGGACTTAAAGTTACTGTCATTAGTACCTCTCCTAGCAAGAAGGATGAAGCCATCAATCACCTTGGTGCTGAT<br>GCATTTTTAGTTAGCACTGATCAAGAGCAAACCCAGAAAGCAATGAGCACAATGGATGGCATAATTGACACAGTATCAGCTCCT<br>CATGCATTGATGCCATTGTTTTCCCTATTGAAACCAAATGGGAAGCTAATTGTTGTTGGTGCACCAAATAAACCAGTTGAATTA<br>GATATTCTATTTCTTGTCATGGGAAGGAAAATGCTCGGAACATCTGCTGTTGGGGGAGTGAAGGAGACACAAGAGATGATCGAT<br>TTTGCAGCAAAACATGGTATAGTTGCAGATGTAGAAGTTGTGGAAATGGAGAATGTGAACAATGCAATGGAGCGGCTTGCGAAG<br>GGGGATGTTAGATATAGATTTGTTCTTGATATTGGGAATGCAACAGTAGCTGTCTGA
CrRedox2<br>*C. roseus*<br>Redox enzyme 1<br>(MF770510.1)	ATGGAAAAGCAAGTTGAGATCCCTGAAGTAGAATTGAATTCAGGACATAAAATGCCAATTGTGGGATACGGAACATGCGTGCCA<br>GAACCCATGCCACCGTTGGAAGAACTAACCGCAATCTTCTTAGATGCAATAAAGGTTGGTTACCGGCACTTCGACACGGCTTCG<br>AGTTACGGCACAGAGGAGGCACTTGGTAAAGCCATAGCTGAGGCTATAAACAGCGGTTTGGTTAAAAGTAGGGAGGAATTCTTC<br>ATCAGTTGTAAGCTGTGGATTGAAGATGCAGATCATGACCTTATCTTGCCTGCCCTCAACCAGTCACTTCAGATTCTTGGGGTT<br>GATTATTTGGATCTATATATGATACACATGCCGGTGAGAGTGAGGAAAGGTGCTCCCATGTTCAATTATTCAAAAGAGGATTTC<br>CTTCCATTTGACATACAAGGTACATGGAAGGCCATGGAAGAGTGCAGCAAACAAGGATTGGCCAAGTCTATTGGTGTCAGCAAC<br>TACTCTGTTGAAAAACTCACTAAACTCCTAGAAACCTCCACTATTCCCCCCGCCGTTAATCAGGTTGAGATGAATGTAGCTTGG<br>CAACAGAGAAAATTGCTGCCATTTTGCAAAGAGAAAAACATTCATATAACATCATGGTCTCCACTCCTATCTTATGGTGTCGCT<br>TGGGGAAGTAATGCTGTCATGGAAAATCCTGTTCTCCAACAAATTGCGGCTTCCAAAGGCAAAACTGTGGCACAGGTGGCACTA<br>AGATGGATATACGAGCAAGGAGCAAGTCTCATTACAAGGACTTCCAACAAGGACAGAATGTTTGAAAATGTTCAAATTTTTGAT<br>TGGGAACTCAGTAAAGAAGAATTGGATCAAATTCATGAAATCCCACAACGTAGGGGTACTTTAGGTGAAGAATTTATGCATCCA<br>GAAGGACCGATCAAATCCCCAGAGGAGCTTTGGGATGGAGACTTGTGA
CrSAT<br>*C. roseus*<br>Stemmadenine O-<br>acetyltransferase<br>(MF770511.1)	ATGGCACCCCAGATGCAGATATTGTCAGAGGAACTGATTCAACCATCATCTCCGACACCCCAAACCTTGAAAACCCATAAACTT<br>TCCCATCTTGATCAAGTTTTATTAACATGTCATATCCCTATTATTCTCTTTTATCCAAATCAATTGGACTCAAATCTCGATCGA<br>GCCCAAAGATCTGAGAATCTAAAACGATCTTTATCAACAGTGTTAACTCAATTTTACCCTTTAGCCGGAAGAATCAATATAAAT<br>TCTTCCGTAGATTGTAATGATTCCGGAGTCCCTTTTCTTGAAGCTCGAGTTCATTCCCAACTCTCAGAAGCAATTAAAAATGTC<br>GCCATAGATGAACTCAATCAATACCTGCCATTCCAACCTTATCCCGGTGGGGAGGAAAGTGGGTTGAAAAAAGATATCCCCTTA<br>GCTGTAAAAATCAGTTGTTTCGAATGTGGCGGAACAGCAATTGGGGTCTGTATTTCCCACAAGATTGCCGATGCGTTGTCCTTG<br>GCTACCTTCCTCAATTCATGGACCGCAACATGCCAGGAAGAGACTGATATTGTTCAACCTAATTTCGATCTGGGATCCCATCAT<br>TTTCCGCCTATGGAAAGCATCCCAGCACCTGAATTTCTACCGGATGAAAATATTGTGATGAAACGGTTCGTGTTCGATAAAGAA<br>AAATTAGAAGCTCTAAAAGCCCAATTAGCTTCCTCTGCAACAGAAGTGAAGAATTCAAGTCGGGTACAAATTGTTATTGCTGTT<br>ATATGGAAGCAATTCATTGACGTGACCCGGGCAAAATTCGATACCAAGAACAAATTAGTAGCAGCTCAAGCAGTGAATTTGAGA<br>TCAAGAATGAATCCACCATTTCCTCAATCTGCTATGGGGAATATAGCCACAATGGCATATGCCGTTGCAGAGGAGGATAAGGAT<br>TTTTCAGATCTTGTCGGTCCATTGAAAACCAGTCTTGCAAAAATTGATGATGAACATGTTAAAGAATTACAAAAAGGAGTAACA<br>TATTTGGATTATGAAGCTGAACCACAAGAATTGTTCTCTTTTAGTAGTTGGTGCAGGCTTGGGTTTTATGATTTGGATTTTGGA<br>TGGGGAAAGCCTGTTTCTGTTTGTACAACAACTGTGCCTATGAAGAATTTGGTATATTTGATGGATACAAGAAATGAAGATGGA<br>ATGGAAGCATGGATCAGCATGGCTGAAGATGAGATGTCTATGCTTTCCTCTGATTTTCTTTCACTTCTGGACACTGATTTTAGC<br>AATTGA

Table S2. Primer sequences used for functional characterization of biosynthetic genes in this study.

**Gene**	**Plasmid**	**Primer direction**	**Sequence (5’-3’)**
Nicotiana benthamiana single transcriptional unit constructs (In-Fusion cloning)			
P19 (TBSV)	3Ω1	Forward	**TTTATGAATTTTGCAGCTCG**ATGGAACGAGCTATACAAGGAAA
		Reverse	**GACAACCACAACAAGCACCG**TCACTCGCTTTCTTTTTCGAAGG
GFP	3Ω1	Forward	**TTTATGAATTTTGCAGCTCG**ATGGTGAGCAAGGGCGAG
		Reverse	**GACAACCACAACAAGCACCG**TCACTTGTACAGCTCGTCCATGC
CrSGD	3Ω1	Forward	**TTTATGAATTTTGCAGCTCG**ATGGGATCTAAAGATGATCAGTC
		Reverse	**GACAACCACAACAAGCACCG**TTAGTATTTTTGCTTCTTGACTAACT
CrGS	3Ω1	Forward	**TTTATGAATTTTGCAGCTCG**ATGGCCGGAGAAACAA
		Reverse	**GACAACCACAACAAGCACCG**TCATTCCTCAAATTTCAATGTATTTC
CrSBE	3Ω1	Forward	**TTTATGAATTTTGCAGCTCG**ATGGATGAGATGATGAACTTCTC
		Reverse	**GACAACCACAACAAGCACCG**TTAATTTCCTTCAACTGCTGATGA
CrGO	3Ω1	Forward	**TTTATGAATTTTGCAGCTCG**ATGGAGTTTTCTTTCTCCTCAC
		Reverse	**GACAACCACAACAAGCACCG**CTAATCGTTAACAAGATGAGGAA
CrRedox1	3Ω1	Forward	**TTTATGAATTTTGCAGCTCG**ATGGCTGATCGCGTGAAGA
		Reverse	**GACAACCACAACAAGCACCG**TCAGACAGCTACTGTTGCAT
CrRedox2	3Ω1	Forward	**TTTATGAATTTTGCAGCTCG**ATGGAAAAGCAAGTTGAGATCC
		Reverse	**GACAACCACAACAAGCACCG**TCACAAGTCTCCATCCCAAA
CrSAT	3Ω1	Forward	**TTTATGAATTTTGCAGCTCG**ATGGCACCCCAGATGCA
		Reverse	**GACAACCACAACAAGCACCG**TCAATTGCTAAAATCAGTGTCCA
TiSGD	3Ω1	Forward	**TTTATGAATTTTGCAGCTCG**ATGCATTCGTATTCAACAACTATGG
		Reverse	**GACAACCACAACAAGCACCG**TCAAAAGACCATATTATTTGTGCCTG
TiGS	3Ω1	Forward	**TTTATGAATTTTGCAGCTCG**ATGGCTGCAGAAACAGC
		Reverse	**GACAACCACAACAAGCACCG**TCATTCATCATATTTCAATGTATTGCCA
TiSBE	3Ω1	Forward	**TTTATGAATTTTGCAGCTCG**ATGGAGATGATGAACTTCTCTTTCA
		Reverse	**GACAACCACAACAAGCACCG**TTAATTTCCTGAAATGATGTTGGGAATTA
TiGO	3Ω1	Forward	**TTTATGAATTTTGCAGCTCG**ATGGAGCTCTCTTTCTCCTC
		Reverse	**GACAACCACAACAAGCACCG**CTATTCGTTGGCAAGATGTG
TiRedox1	3Ω1	Forward	**TTTATGAATTTTGCAGCTCG**ATGGCTGATCGCGTCAA
		Reverse	**GACAACCACAACAAGCACCG**CTAGGCAGCGCCC
TiRedox2	3Ω1	Forward	**TTTATGAATTTTGCAGCTCG**ATGGAAAGGGAAGTTCAAATCC
		Reverse	**GACAACCACAACAAGCACCG**TTACAAATCTCCATCCCAGAGT
TiSAT	3Ω1	Forward	**TTTATGAATTTTGCAGCTCG**ATGACTTCCCAGATGGATGTAG
		Reverse	**GACAACCACAACAAGCACCG**TCACTTGCTAAAATCACTGTCTAC
Sequence verification primers for 3Ω1 construct		Forward	GATGAAAAAGCCCTAAAATTGGAG
		Reverse	ATTATTCACAAATGAGAAACAGAATGG
*The In-Fusion cloning overhangs for 3Ω1 cloning are indicated in **bold**.			

Gene	Plasmid	Primer direction	Sequence (5’-3’)
Escherichia coli expression using pOPINF constructs (In-Fusion cloning): N-terminal His_6-tag			
CrSGD	pOPINF	Forward	**AAGTTCTGTTTCAGGGCCCG**GGATCTAAAGATGATCAGTC
		Reverse	**ATGGTCTAGAAAGCTTTA**GTATTTTTGCTTCTTGACTAACT
CrRedox1	pOPINF	Forward	**AAGTTCTGTTTCAGGGCCCG**GCTGATCGCGTGAAGA
		Reverse	**ATGGTCTAGAAAGCTTTA**GACAGCTACTGTTGCAT
CrRedox2	pOPINF	Forward	**AAGTTCTGTTTCAGGGCCCG**GAAAAGCAAGTTGAGATCC
		Reverse	**ATGGTCTAGAAAGCTTTA**CAAGTCTCCATCCCAAA
CrSAT	pOPINF	Forward	**AAGTTCTGTTTCAGGGCCCG**GCACCCCAGATGCA
		Reverse	**ATGGTCTAGAAAGCTTTA**ATTGCTAAAATCAGTGTCCA
TiSGD	pOPINF	Forward	**AAGTTCTGTTTCAGGGCCCG**CATTCGTATTCAACAACTATGG
		Reverse	**ATGGTCTAGAAAGCTTTA**AAAGACCATATTATTTGTGCCTG
TiRedox1	pOPINF	Forward	**AAGTTCTGTTTCAGGGCCCG**GCTGATCGCGTCAA
		Reverse	**ATGGTCTAGAAAGCTTTA**GGCAGCGCCCA
TiRedox2	pOPINF	Forward	**AAGTTCTGTTTCAGGGCCCG**GAAAGGGAAGTTCAAATCC
		Reverse	**ATGGTCTAGAAAGCTTTA**CAAATCTCCATCCCAGAGT
TiSAT	pOPINF	Forward	**AAGTTCTGTTTCAGGGCCCG**ACTTCCCAGATGGATGTAG
		Reverse	**ATGGTCTAGAAAGCTTTA**CTTGCTAAAATCACTGTCTAC
Sequence verification primers for pOPINF construct		Forward	TAATACGACTCACTATAGGG
		Reverse	TAGCCAGAAGTCAGATGCT
Escherichia coli expression using pET-28a(+) constructs (In-Fusion cloning): C-terminal His_6-tag			
CrGS	pET28a(+)	Forward	**AGGAGATATACCATGG**ATGGCCGGAGAAACA
		Reverse	**GGTGGTGGTGCTCGAG**TTCCTCAAATTTCAATGTATTTCC
TiGS	pET28a(+)	Forward	**AGGAGATATACCATGG**ATGGCTGCAGAAACAG
		Reverse	**GGTGGTGGTGCTCGAG**TTCATCATATTTCAATGTATTGCCAA
Sequence verification primers for pET28a construct		Forward	TAATACGACTCACTATAGGG
		Reverse	GCTAGTTATTGCTCAGCGG
*In-Fusion cloning overhangs for pOPINF and pET-28a(+) cloning are indicated in **bold**.			

Gene	Plasmid	Primer direction	Sequence (5’-3’)
Saccharomyces cerevisiae expression using pESC-HIS (MCS2) constructs (In-Fusion cloning)			
CrGO	pESC-HIS	Forward	**GAGAAAAAACCCCGGATCC**ATGGAGTTTTCTTTCTCCTCAC
		Reverse	**ACTTCTGTTCCATGTCGAC**CTAATCGTTAACAAGATGAGGAA
CrSBE	pESC-HIS	Forward	**GAGAAAAAACCCCGGATCC**ATGGATGAGATGATGAACTTCTC
		Reverse	**ACTTCTGTTCCATGTCGAC**TTAATTTCCTTCAACTGCTGATGA
TiGO	pESC-HIS	Forward	**GAGAAAAAACCCCGGATCC**ATGGAGCTCTCTTTCTCCTC
		Reverse	**ACTTCTGTTCCATGTCGAC**CTATTCGTTGGCAAGATGTG
TiSBE	pESC-HIS	Forward	**GAGAAAAAACCCCGGATCC**ATGGAGATGATGAACTTCTCTTTCA
		Reverse	**ACTTCTGTTCCATGTCGAC**TTAATTTCCTGAAATGATGTTGGGAATTA
Sequence verification primers pESC-HIS construct		Forward	ATGATTTTTGATCTATTAACAGATA
		Reverse	GTATAATGTTACATGCGTACAC
*In-Fusion cloning overhangs for pESC-HIS (MCS2) cloning are indicated in **bold**.			

Table S3. Primer sequences used for the generation CDS parts for the assembly of multigene constructs using GoldenBraid 2.0 cloning in this study.

Gene	Level 1 CDS	Primer direction	Sequence (5’-3’)
Nicotiana benthamiana multigene constructs			
Level 1 CDS fragment for 3α1 or 3α2 GoldenBraid 2.0 assembly			
Domestication of TiRedox2 for pUPD entry	Frag. A	Forward	GCGCCGTCTCGCTCGAATGGAAAGGGAAGTTCAAATCC
		Reverse	GCGCCGTCTCGGGGACCATGCGGTGACATGA
	Frag. B	Forward	GCGCCGTCTCGTCCCCTCTGTTATCTTATGGT
		Reverse	GCGCCGTCTCGCTCGAAGCTTACAAATCTCCATCCCAGAG
	Full length	Forward	GCGCCGTCTCGCTCGAATGGAAAGGGAAGTTCAAATCC
		Reverse	GCGCCGTCTCGCTCGAAGCTTACAAATCTCCATCCCAGAG
Domestication of TiSBE for pUPD entry	Frag. A	Forward	GCGCCGTCTCGCTCGAATGGAGATGATGAACTTCTCTTTC
		Reverse	GCGCCGTCTCGGGATCTCTTCCCACTGCCCA
	Frag. B	Forward	GCGCCGTCTCGATCCTGAACTTTGGGACGATG
		Reverse	GCGCCGTCTCGCTCGAAGCTTAATTTCCTGAAATGATGTTGGG
	Full length	Forward	GCGCCGTCTCGCTCGAATGGAGATGATGAACTTCTCTTTC
		Reverse	GCGCCGTCTCGCTCGAAGCTTAATTTCCTGAAATGATGTTGGG
TiGO	Full length	Forward	**TTCAGAGGTCTCTA**ATGGAGCTCTCTTTCTCCTC
		Reverse	**AGCGTGGGTCTCGAAGC**CTATTCGTTGGCAAGATGTG
TiRedox1	Full length	Forward	**TTCAGAGGTCTCTA**ATGGCTGATCGCGTCAAGA
		Reverse	**AGCGTGGGTCTCGAAGC**CTAGGCAGCGCCCATTG
TiSAT	Full length	Forward	**TTCAGAGGTCTCTA**ATGACTTCCCAGATGGATGT
		Reverse	**AGCGTGGGTCTCGAAGC**TCACTTGCTAAAATCACTGTCTA
P19	Full length	Forward	**TTCAGAGGTCTCTA**ATGGAACGAGCTATACAAGGAAA
		Reverse	**AGCGTGGGTCTCGAAGC**TCACTCGCTTTCTTTTTCGAAGG
Plasmid validation	P19	Forward	TATTTAAGAGATATCTCAGATACG
		Reverse	CTGGTCGAAACCGAAAAATC

	TiGO	Forward	CAGGTTGGAGGATCTGTTCCC
		Reverse	TGCCTCAAGAGAACGCTGAG
	TiRedox1	Forward	CTTCCTCTCGATGCTGGTGC
		Reverse	AGTCCAAAAGCCTTGGCGAA
	TiRedox2	Forward	GTAAGAGTGAGGCAGGGTGC
		Reverse	AACTGCAGGAGGAATGGTGG
	TiSAT	Forward	ACCCATGTTGTGCTGCCTAA
		Reverse	GGATTCTTCACCTCCGAGGC
Overhangs designed for non-domesticated sequences 3α1 or 3α2 GoldenBraid assembly			

Table S4. Primer sequences used for CDS (domesticated) amplification to generate C-terminal eYFP fusion constructs for subcellular localization with GoldenBraid 2.0 in this study.

**Gene**	**Plasmid**	**Primer direction**	**Sequence (5'-3')**
Nicotiana benthamiana single transcriptional unit constructs (3α1, GoldenBraid 2.0)			
TiSGD	3α1	Forward	**TTCAGAGGTCTCTA**ATGCATTCGTATTCAACAACTATG
		Reverse	**AGCGTGGGTCTCGCTGC***AGAAGAACCAGAACC*AAAGACCATATTATTTGTGCCTG
TiGS	3α1	Forward	**TTCAGAGGTCTCTA**ATGGCTGCAGAAACAGC
		Reverse	**AGCGTGGGTCTCGCTGC***AGAAGAACCAGAACC*TTCATCATATTTCAATGTATTGCCAAT
TiSBE	3α1	Forward	**TTCAGAGGTCTCTA**ATGGAGATGATGAACTTCTCTTTCA
		Reverse	**AGCGTGGGTCTCGCTGC***AGAAGAACCAGAACC*ATTTCCTGAAATGATGTTGGGAATTA
TiGO	3α1	Forward	**TTCAGAGGTCTCTA**ATGGAGCTCTCTTTCTCCT
		Reverse	**AGCGTGGGTCTCGCTGC***AGAAGAACCAGAACC*TTCGTTGGCAAGATGTG
TiRedox1	3α1	Forward	**TTCAGAGGTCTCTA**ATGGCTGATCGCGT
		Reverse	**AGCGTGGGTCTCGCTGC***AGAAGAACCAGAACC*GGCAGCGCCCA
TiRedox2	3α1	Forward	**TTCAGAGGTCTCTA**ATGGAAAGGGAAGTTCAAATC
		Reverse	**AGCGTGGGTCTCGCTGC***AGAAGAACCAGAACC*CAAATCTACATCCCAGAGTTC
TiSAT	3α1	Forward	**TTCAGAGGTCTCTA**ATGACTTCCCAGATGGATGTA
		Reverse	**AGCGTGGGTCTCGCTGC***AGAAGAACCAGAACC*CTTGCTAAAATCACTGTCTACAAG
Sequence verification primers 3α1 construct		Forward	GATGAAAAAGCCCTAAAATTGGAG
		Reverse	ATTATTCACAAATGAGAAACAGAATGG
*GoldenBraid 2.0 compatible cloning overhangs for 3α1 transcriptional unit assembly are indicated in **bold**.<br>**GSGSS linker sequences are indicated by *italics*.<br>Transcriptional unit = Ubq10Promoter+5UTR+Gene+C-terminal-YFP+3UTR+Ubq10Terminator			

Table S5. Primer sequences used for qPCR analysis in this study.

**Target gene**	**Primer direction**	**Sequence (5'-3')**
CrSGD	Forward	ATTGATGCTCGGGAAAGGGG
	Reverse	AGACGGCTTCCTACAAGAGC
CrGS	Forward	ATGGCCGGAGAAACAACCAA
	Reverse	CCGTTCACCAGGGACTCTTC
TiSGD	Forward	TGGTGGCTTCTTGAGTTCCC
	Reverse	GTTCTGTGCCTGGATCACCT
TiGS	Forward	ATTACTGCCCAGAGCCCAAC
	Reverse	GTCCTGTGGGAAGTTCTCGG
Nicotiana benthamiana Elongation Factor 1 alpha (NbEF1α) – Reference gene (PQ008965.1)	Forward	AGCGTGGTTATGTTGCCTCA
	Reverse	GACCTGGGAGGTGAAACTGG

Supporting NMR Tables

Table NMR S1. NMR shifts of stemmadenine as formate salt.

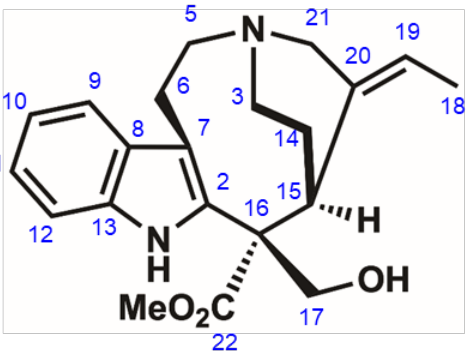

stemmadenine (formate salt) (MeOH-d_3, 700 MHz, 298 K)				
**pos.**	**δ_H**	**mult.**	**J_{HH}**	**δ_C**
1(NH)	10.14	-	-	-
2	-	-	-	134.9
3α	3.10	*ddd*	13.6/13.4/6.4	47.2
3β	3.27	*dd*	13.4/6.1	47.2
5α	3.47	*m*	-	56.8
5β	3.38	*m***	-	56.8
6α	3.54	*m*	-	24.4
6β	3.38	*m***	-	24.4
7	-	-	-	110.9
8	-	-	-	128.2
9	7.53	*d*	7.9	118.6
10	7.07	*dd*	7.9/7.6	120.4
11	7.13	*dd*	8.1/7.6	123.0
12	7.39	*d*	8.1	112.5
13	-	-	-	137.0
14α	2.36	*ddd*	16.1/12.9/6.6	26.1
14β	2.61	*m*	-	26.1
15α	3.80	*dd*	12.7/3.0	36.5
16	-	-	-	61.7
17a	4.35	*s*	-	69.4
17b	4.35	*s*	-	69.4
18	1.77	*dd*	6.9/2.0	14.3
19	5.57	*q*	6.9	130.9
20	-	-	-	129.0
21α	3.37	*m***	-	55.1
21β	2.91	*brd*	14.8	55.1
22	-	-	-	174.2
OMe	3.80	*s*	-	53.0

** overlapped signals *J* unresolved, 700 MHz in MeOH-d_3

Table NMR S2. NMR shifts of 16(*R*)-19*E*-isositirikine as acetate salt.

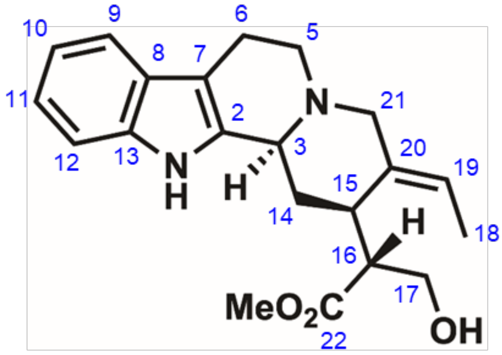

16(*R*)-19*E*-isositirikine (acetate salt) ($CDCl_3$, 700 MHz, 298 K)				
**pos.**	**δ_H**	**mult.**	**J_{HH}**	**δ_C**
1(NH)	9.00	-	-	-
2	-	-	-	131.5
3α	4.55	*m*	-	52.7
5α	3.24	*ddd*	13.2/11.3/5.2	50.4
5β	3.34	*ddd*	13.2/5.8/2.1	50.4
6α	2.76	*m*	-	17.4
6β	2.98	*m*	-	17.4
7	-	-	-	107.2
8	-	-	-	127.3
9	7.44	*d*	7.8	118.3
10	7.09	*dd*	7.8/7.4	120.0
11	7.16	*dd*	8.0/7.4	122.3
12	7.37	*d*	8.0	111.7
13	-	-	-	136.7
14α	2.30	*m***	-	29.2
14β	2.31	*m***	-	29.2
15α	3.10	*ddd*	11.2/4.7/4.7	32.5
16	2.41	*ddd*	11.2/7.8/5.0	49.3
17a	3.54	*dd*	11.5/7.8	62.1
17b	3.47	*dd*	11.5/5.0	62.1
18	1.64	*brd*	6.7	13.7
19	5.70	*q*	-	126.8
20	-	-	-	131.4
21α	3.21	*d*	12.9	51.6
21β	3.56	*d*	12.9	51.6
22	-	-	-	175.3
OMe	3.79	*s*	-	52.6

** overlapped signals *J* unresolved, 700 MHz in $CDCl_3$

Table NMR S3. NMR shifts of 16(*S*)-19*E*-isositirikine as acetate salt.

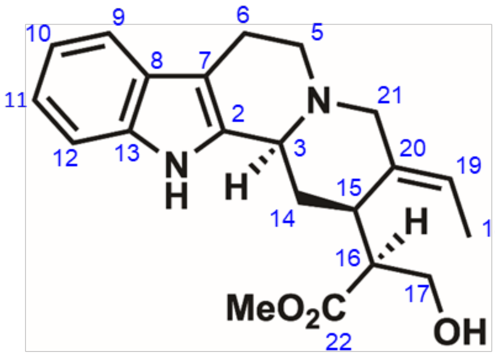

16(*R*)-19*E*-isositirikine (acetate salt) (CDCl3, 700 MHz, 298 K)				
**pos.**	**δH**	**mult.**	**JHH**	**δC**
1(NH)	9.76	-	-	-
2	-	-	-	130.6
3α	4.65	*m*	-	52.8
5α	3.16	*ddd*	12.2/11.1/5.1	50.2
5β	3.35	*m***	-	50.2
6α	2.77	*m*	-	17.4
6β	3.00	*m*	-	17.4
7	-	-	-	106.5
8	-	-	-	127.0
9	7.41	*d*	7.7	118.4
10	7.07	*brdd*	7.7/7.5	120.0
11	7.13	*brdd*	8.1/7.5	122.4
12	7.31	*d*	8.1	111.6
13	-	-	-	136.9
14α	2.37	*m***	-	28.5
14β	2.59	*m*	-	28.5
15α	3.25	*m*	-	32.8
16	2.35	*m***	-	48.2
17a	3.90	*m*	-	62.1
17b	3.90	*m*	-	62.1
18	1.57	*dd*	6.8/1.1	13.4
19	5.64	*q*	6.8	126.9
20	-	-	-	131.1
21α	3.38	*d*	12.5	52.3
21β	3.82	*d*	12.5	52.3
22	-	-	-	174.5
OMe	3.47	*s*	-	51.8
** overlapped signals *J* unresolved, 700 MHz in CDCl3				

Table NMR S4. NMR shifts of 16(*R*)-17D-19*E*-isositirikine as acetate salt.

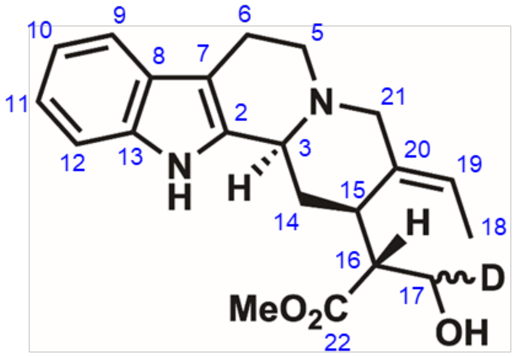

16(*R*)-17D-19*E*-isositirikine (acetate salt) (CDCl3, 700 MHz, 298 K)				
**pos.**	**δH**	**mult.**	**JHH**	**δC**
1(NH)	9.31	-	-	-
2	-	-	-	130.8
3α	4.61	*m*	-	52.6
5α	3.26	*m*	-	49.9
5β	3.31	*m***	-	49.9
6α	2.78	*m*	-	17.4
6β	2.94	*m*	-	17.4
7	-	-	-	106.7
8	-	-	-	127.0
9	7.41	*d*	7.9	118.3
10	7.07	*brdd*	7.9/7.5	119.9
11	7.14	*brdd*	8.0/7.5	122.4
12	7.35	*brd*	8.0	111.7
13	-	-	-	136.7
14α	2.35	*ddd*	15.3/5.7/5.7	28.7
14β	2.29	*m*	-	28.7
15α	3.10	*m*	-	32.4
16	2.40	*m*	-	49.1
17a*	3.52	*d*	7.5	61.5
17b*	3.46	*d*	5.1	61.5
18	1.61	*brd*	6.8	13.7
19	5.71	*q*	6.8	128.0
20	-	-	-	130.4
21α	3.35	*d*	12.3	51.8
21β	3.55	*d*	12.3	51.8
22	-	-	-	175.0
OMe	3.75	*s*	-	52.5
** overlapped signals *J* unresolved, *0.5H, 700 MHz in CDCl3				

Table NMR S5. NMR shifts of 16(*S*)-17D-19*E*-isositirikine as acetate salt.

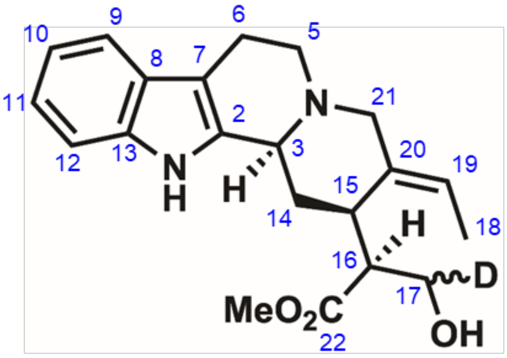

16(*R*)-17D-19*E*-isositirikine (acetate salt)
($CDCl_3$, 700 MHz, 298 K)

pos.	δ_H	mult.	J_{HH}	δ_C
1(NH)	10.29	-	-	-
2	-	-	-	130.1
3α	4.71	*m*	-	52.8
5α	3.17	*m***	-	50.1
5β	3.35	*m*	-	50.1
6α	2.76	*m*	-	17.1
6β	3.00	*m*	-	17.1
7	-	-	-	106.0
8	-	-	-	126.8
9	7.38	*d*	7.8	118.3
10	7.05	*brdd*	7.8/7.5	119.9
11	7.11	*brdd*	8.1/7.5	122.4
12	7.30	*brd*	8.1	111.7
13	-	-	-	137.0
14α	2.37	*ddd*	15.1/5.9/5.9	28.2
14β	2.64	*m*	-	28.2
15α	3.19	*m*	-	32.6
16	2.29	*m*	-	48.1
17a*	3.87	*m*	-	61.6
17b*	3.87	*m*	-	61.6
18	1.55	*brd*	6.7	13.4
19	5.64	*q*	6.7	127.4
20	-	-	-	130.7
21α	3.40	*d*	12.3	51.7
21β	3.82	*d*	12.3	51.7
22	-	-	-	174.4
OMe	3.44	*s*	-	51.7

** overlapped signals *J* unresolved, *0.5H, 700 MHz in $CDCl_3$

Table NMR S6. NMR shifts of condylocarpine as formate salt.

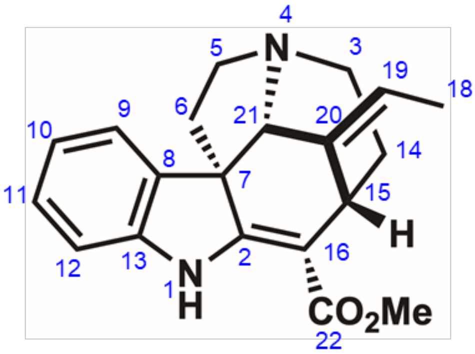

condylocarpine (formate salt)
(MeOH-*d_3*, 700 MHz, 298 K)

pos.	δ_H	mult.	J_{HH}	δ_C
1(NH)	9.13	-	-	-
2	-	-	-	168.7
3α	3.05	*m*	-	47.2
3β	3.22	*m***	-	47.2
5α	3.23	*m***	-	52.9
5β	3.40	*m*	-	52.9
6α	2.87	*m*	-	44.8
6β	2.08	*m*	-	44.8
7	-	-	-	60.0
8	-	-	-	134.6
9	7.32	*d*	7.3	121.1
10	6.91	*dd*	7.6/7.3	122.2
11	7.15	*dd*	7.8/7.6	129.5
12	6.92	*d*	7.8	111.3
13	-	-	-	146.0
14a	2.01	*m***	-	27.9
14b	2.01	*m***	-	27.9
15	4.07	*dd*	4.7/4.7	29.4
16	-	-	-	101.6
18	1.64	*d*	6.7	13.0
19	5.49	*q*	6.7	122.0
20	-	-	-	135.2
21β	4.51	*brs*	-	69.6
22	-	-	-	168.5
OMe	3.80	*s*	-	51.7

** overlapped signals *J* unresolved, 700 MHz in MeOH-*d_3*

Supporting Figures

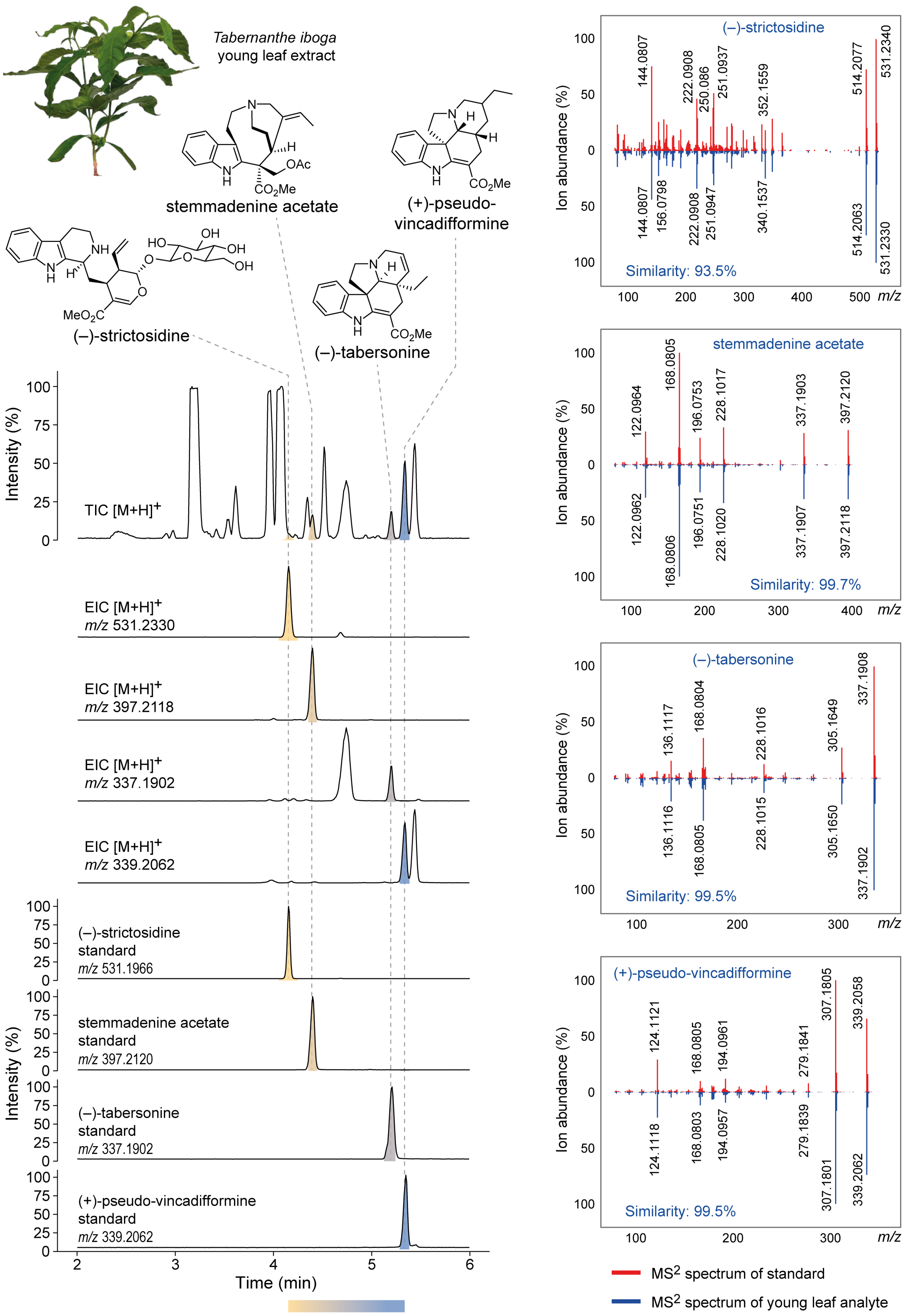

Abbreviation: TIC = Total ion chromatogram, EIC = Extracted ion chromatogram

Figure S1. Monoterpene indole alkaloids (MIAs) detected in the young leaf of *Tabernanthe iboga*. LC-MS chromatograms showing the presence of early-pathway MIAs in young leaves of *T. iboga*. MS^2 spectra of authentic standards and corresponding analytes are shown.

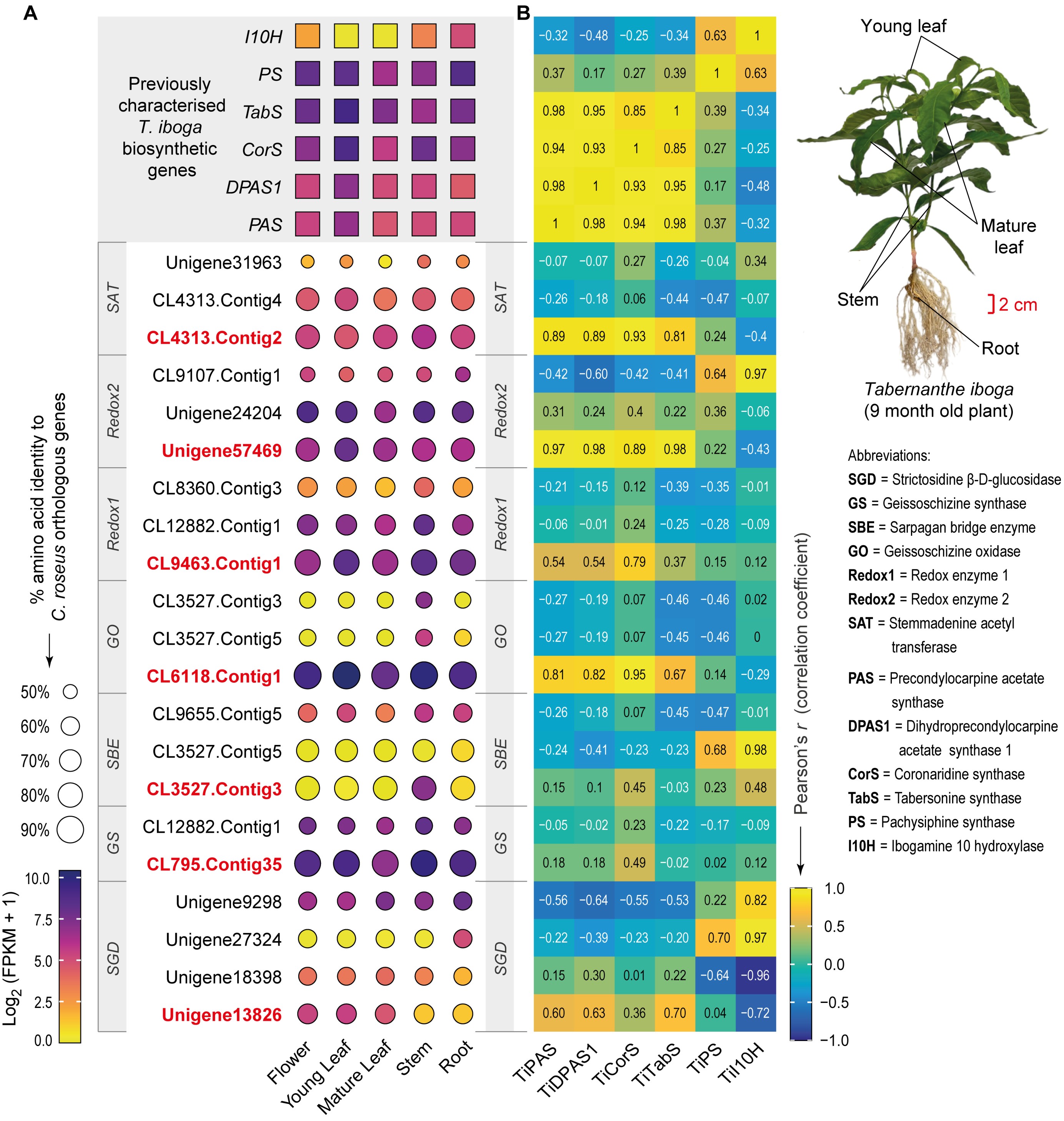

	TiPAS	TiDPAS1	TiCorS	TiTabS	TiPS	TiI10H
I10H	−0.32	−0.48	−0.25	−0.34	0.63	1
PS	0.37	0.17	0.27	0.39	1	0.63
TabS	0.98	0.95	0.85	1	0.39	−0.34
CorS	0.94	0.93	1	0.85	0.27	−0.25
DPAS1	0.98	1	0.93	0.95	0.17	−0.48
PAS	1	0.98	0.94	0.98	0.37	−0.32
Unigene31963	−0.07	−0.07	0.27	−0.26	−0.04	0.34
CL4313.Contig4	−0.26	−0.18	0.06	−0.44	−0.47	−0.07
CL4313.Contig2	0.89	0.89	0.93	0.81	0.24	−0.4
CL9107.Contig1	−0.42	−0.60	−0.42	−0.41	0.64	0.97
Unigene24204	0.31	0.24	0.4	0.22	0.36	−0.06
Unigene57469	0.97	0.98	0.89	0.98	0.22	−0.43
CL8360.Contig3	−0.21	−0.15	0.12	−0.39	−0.35	−0.01
CL12882.Contig1	−0.06	−0.01	0.24	−0.25	−0.28	−0.09
CL9463.Contig1	0.54	0.54	0.79	0.37	0.15	0.12
CL3527.Contig3	−0.27	−0.19	0.07	−0.46	−0.46	0.02
CL3527.Contig5	−0.27	−0.19	0.07	−0.45	−0.46	0
CL6118.Contig1	0.81	0.82	0.95	0.67	0.14	−0.29
CL9655.Contig5	−0.26	−0.18	0.07	−0.45	−0.47	−0.01
CL3527.Contig5	−0.24	−0.41	−0.23	−0.23	0.68	0.98
CL3527.Contig3	0.15	0.1	0.45	−0.03	0.23	0.48
CL12882.Contig1	−0.05	−0.02	0.23	−0.22	−0.17	−0.09
CL795.Contig35	0.18	0.18	0.49	−0.02	0.02	0.12
Unigene9298	−0.56	−0.64	−0.55	−0.53	0.22	0.82
Unigene27324	−0.22	−0.39	−0.23	−0.20	0.70	0.97
Unigene18398	0.15	0.30	0.01	0.22	−0.64	−0.96
Unigene13826	0.60	0.63	0.36	0.70	0.04	−0.72

Figure S2. Early pathway candidate identification and selection. (**A**) Expression profiles of *Tabernanthe iboga* genes orthologous to functionally characterized *Catharanthus roseus* biosynthetic genes (SGD to SAT) across various tissues. The heatmap indicates transcript abundance in bulk tissue samples across five tissues of the *T. iboga* plant. At the same time, the size of each dot in the dot plot reflects the % amino acid identity to the corresponding *C. roseus* enzyme. (**B**) Pearson correlation coefficients of *T. iboga* early pathway candidates (SGD – SAT) to previously characterized *T. iboga* genes are also shown. The *T. iboga* enzymes PAS (1), DPAS1 (1), CorS (1), TabS (1), PS (2), and I10H (1) were used as bait genes to identify candidates by co-expression, with positively correlated genes prioritized (highlighted in red) for further functional testing as putative biosynthetic genes of the stemmadenine acetate biosynthetic pathway.

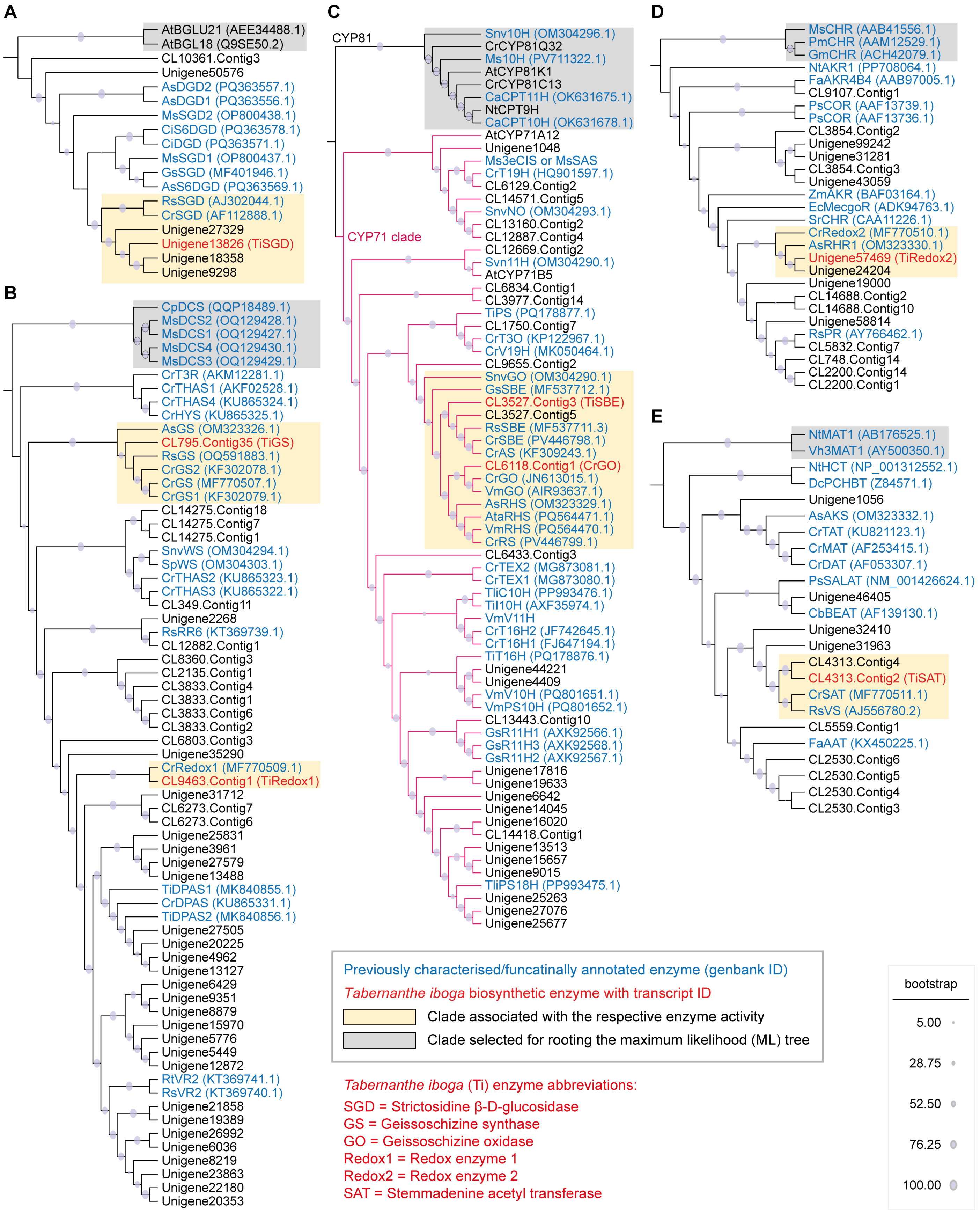

Figure S3. Phylogenetic relationships of stemmadenine biosynthetic enzymes with previously characterized plant natural product biosynthetic enzymes. Maximum likelihood phylogenetic trees were generated to examine the relationships between stemmadenine biosynthetic enzymes and their plant homologs. (**A**) Relationship of SGD with other characterized and annotated plant SGD enzymes. (**B**) Relationship of GS and Redox1 with medium chain alcohol dehydrogenase/reductase (MDR) family enzymes involved in monoterpene indole alkaloid (MIA) biosynthesis. (**C**) Relationship of GO with cytochrome P450 enzymes of the CYP71 family associated with MIA biosynthesis. (**D**) Relationship of Redox2 with known plant aldo-keto reductases (AKRs). (**E**) Relationship of SAT with a subset of plant acetyl transferases involved in plant natural product biosynthesis. All trees shown were inferred using maximum likelihood methods (1000 bootstraps). Trees are rooted at the clades highlighted in grey boxes.

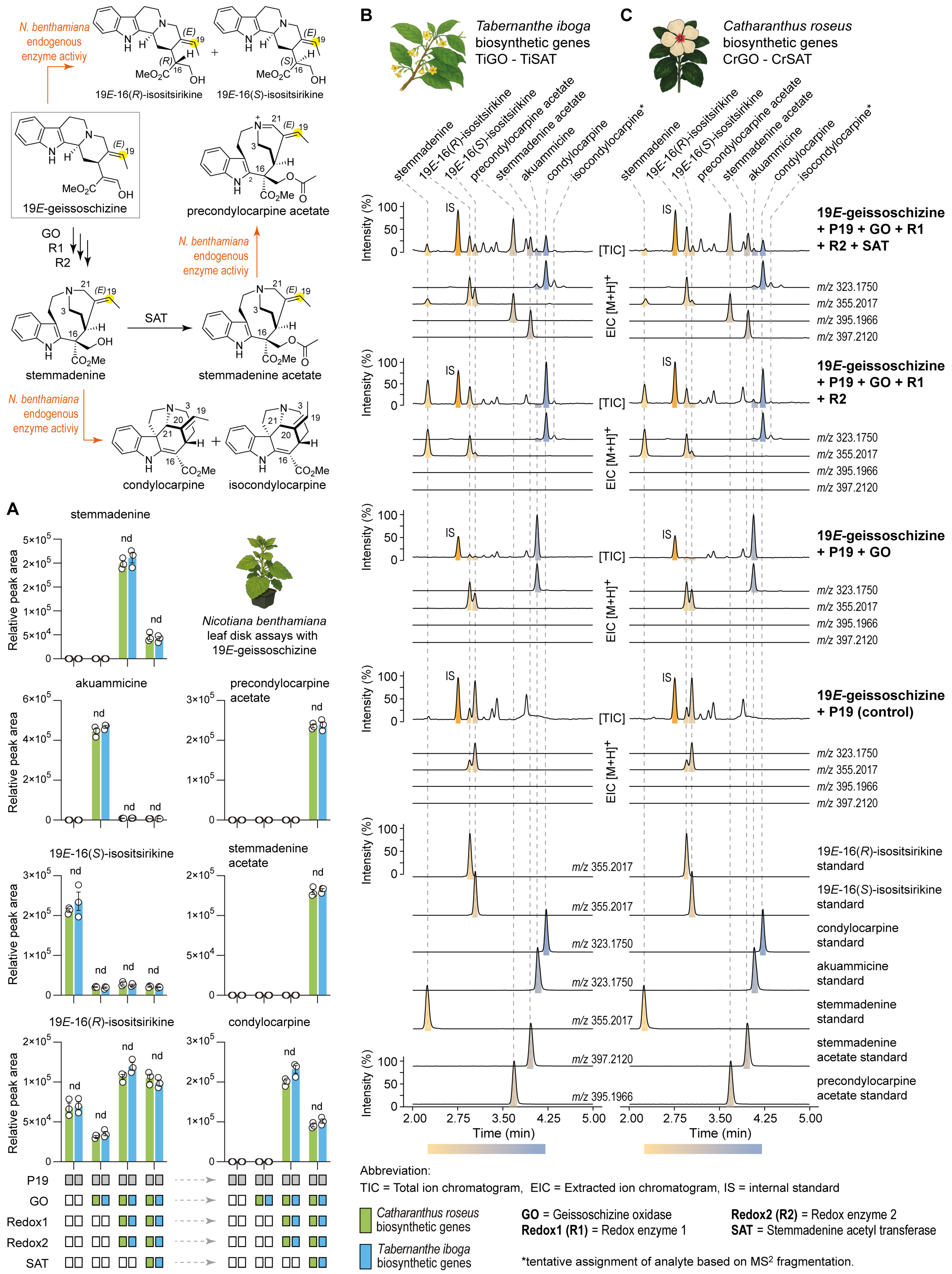

Figure S4. Heterologous production of stemmadenine acetate in *N. benthamiana* catalyzed by *Tabernanthe iboga* genes. (A) Stepwise pathway reconstitution of *T. iboga* candidate genes (blue) involved in stemmadenine acetate biosynthesis in *Nicotiana benthamiana*, starting from the substrate 19*E*-geissoschizine, and benchmarked against functionally characterized *Catharanthus roseus* genes (green) (3). Quantitative analysis shows no statistically significant difference in the accumulation of biosynthetic products from GO to SAT between the *T. iboga* and *C. roseus* gene sets (unpaired t-test, $n = 3$ biological replicates, nd = not significant $P > 0.05$). (**B**) Representative LC-MS chromatogram of biosynthetic products generated by stepwise reconstitution of *T. iboga* biosynthetic genes. (**C**) Representative LC-MS chromatogram of products generated using the corresponding *C. roseus* biosynthetic genes. Endogenous *N. benthamiana* metabolic activity affecting monoterpene indole alkaloids (MIAs) is also shown, revealing background modifications that occur during pathway reconstitution in this heterologous host. Relative peak area is normalized to the internal standard (IS). Bar graphs are plotted as the mean ± SD.

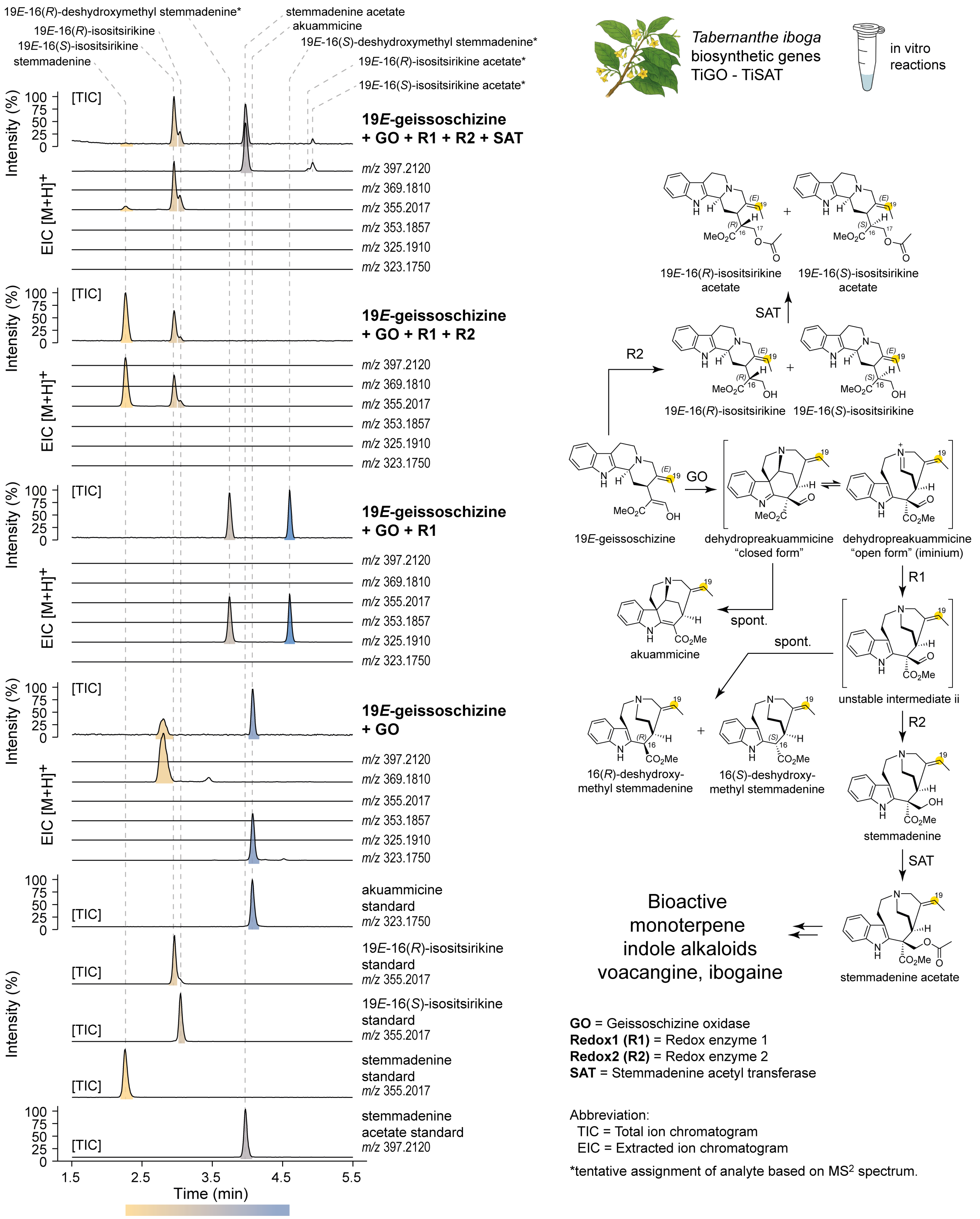

Figure S5. Stepwise in vitro reconstitution of the stemmadenine acetate biosynthetic pathway from 19*E*-geissoschizine using *Tabernanthe iboga* biosynthetic enzymes. LC-MS chromatograms showing the sequential enzymatic conversion of 19*E*-geissoschizine to stemmadenine acetate via the action of *T. iboga* GO, Redox1, Redox2, and SAT. Enzymes were incubated in combination to demonstrate stepwise pathway progression from 19*E*-geissoschizine substrate. Reaction products were identified based on comparison with authentic standards using retention times and MS^2 fragmentation spectra.

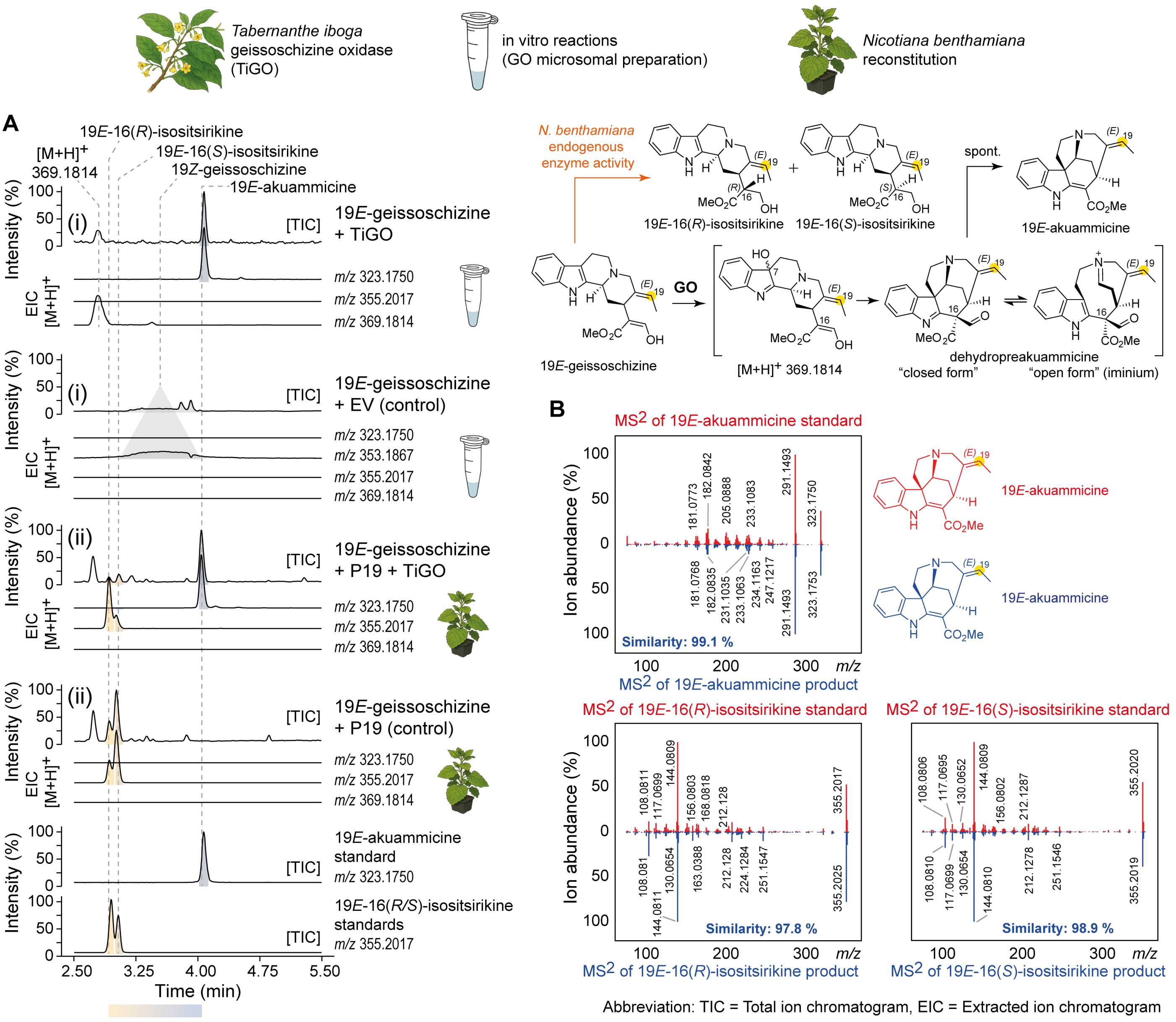

Figure S6. Functional characterization of *Tabernanthe iboga* geissoschizine oxidase (TiGO) activity with 19*E*-geissoschizine. (**A**) (i) LC-MS chromatograms of in vitro enzymatic assays using yeast microsomes expressing TiGO incubated with 19*E*-geissoschizine as substrate. (ii) LC-MS chromatograms of TiGO transiently expressed in *N. benthamiana* with the P19 viral silencing suppressor, assayed in leaf discs supplied with 19*E*-geissoschizine. In both systems, TiGO catalyzed the oxidation of 19*E*-geissoschizine to produce the thermodynamic product akuammicine (19*E*-akuammicine) in the absence of downstream biosynthetic enzymes. The endogenous metabolic activity of *N. benthamiana* led to the reduction of 19*E*-geissoschizine to 19*E*-isositsirikine isomers. (**B**) MS^2 spectrum of TiGO reaction products detected in both in vivo and in vitro assays, compared with spectra of authentic standards.

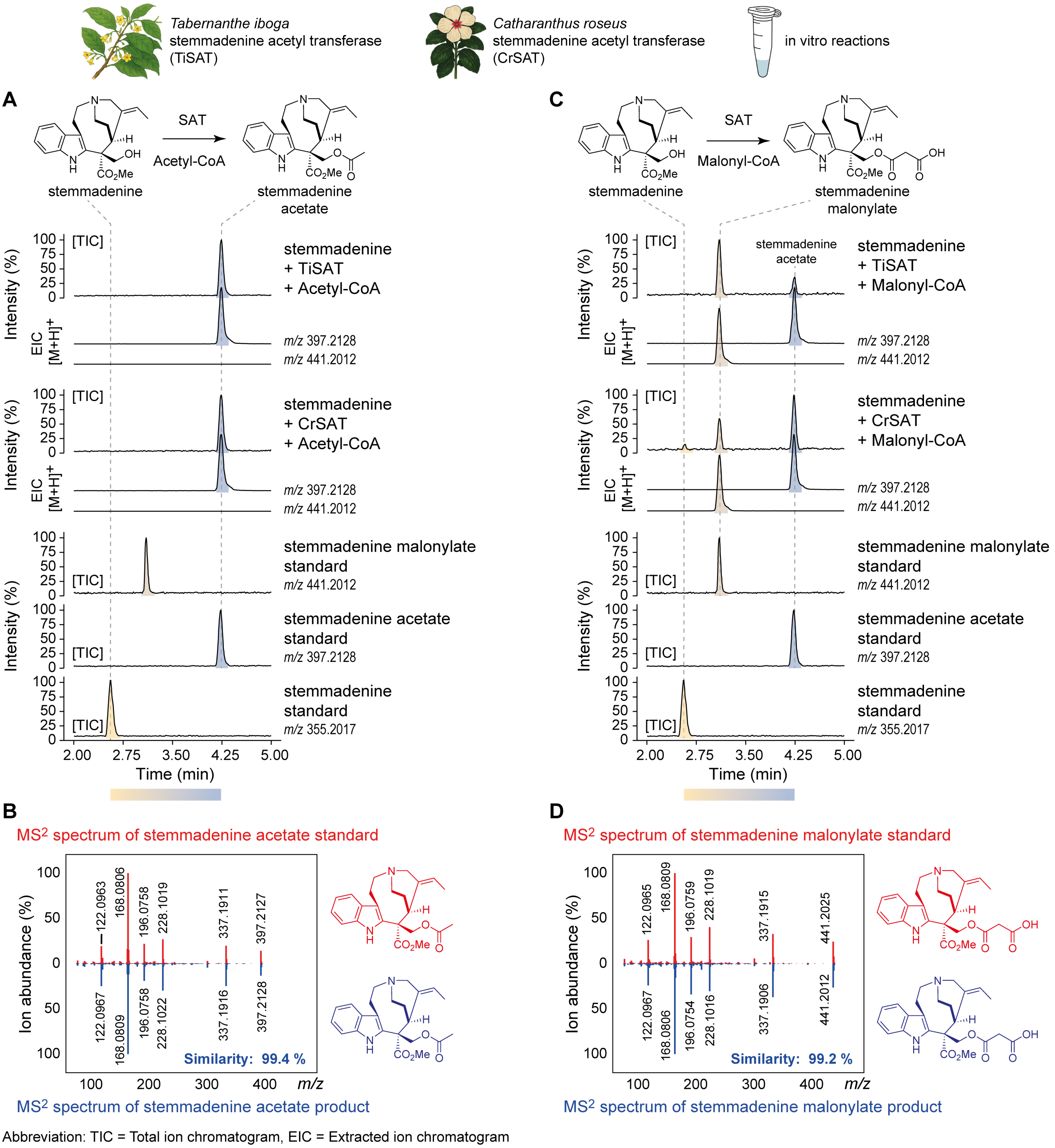

Figure S7. Functional characterization of *Tabernanthe iboga* stemmadenine acetyltransferase (TiSAT) in vitro. (**A**) LC-MS chromatograms of in vitro enzymatic assays using heterologously expressed TiSAT incubated with the substrate stemmadenine and acetyl-CoA as the acyl donor. The previously characterized *Catharanthus roseus* stemmadenine acetyltransferase (CrSAT) (3, 4) was incubated as a positive control under identical conditions. TiSAT catalyzed the acetylation of stemmadenine to yield stemmadenine acetate, consistent with the activity of CrSAT. (**B**) MS^2 spectrum of stemmadenine acetate generated by TiSAT, compared with that of the authentic standard. (**C**) LC-MS chromatograms of in vitro assays of TiSAT incubated with stemmadenine and malonyl-CoA as donor substrate. CrSAT was assayed in parallel. Both TiSAT and CrSAT catalyzed the malonylation of stemmadenine to produce stemmadenine malonylate. In addition, stemmadenine acetate was detected in both reactions, likely due to the co-purification of acetyl-CoA from the heterologous expression systems. (**D**) MS^2 spectra of stemmadenine malonylate produced in the presence of malonyl-CoA by TiSAT, confirming the identity of the product.

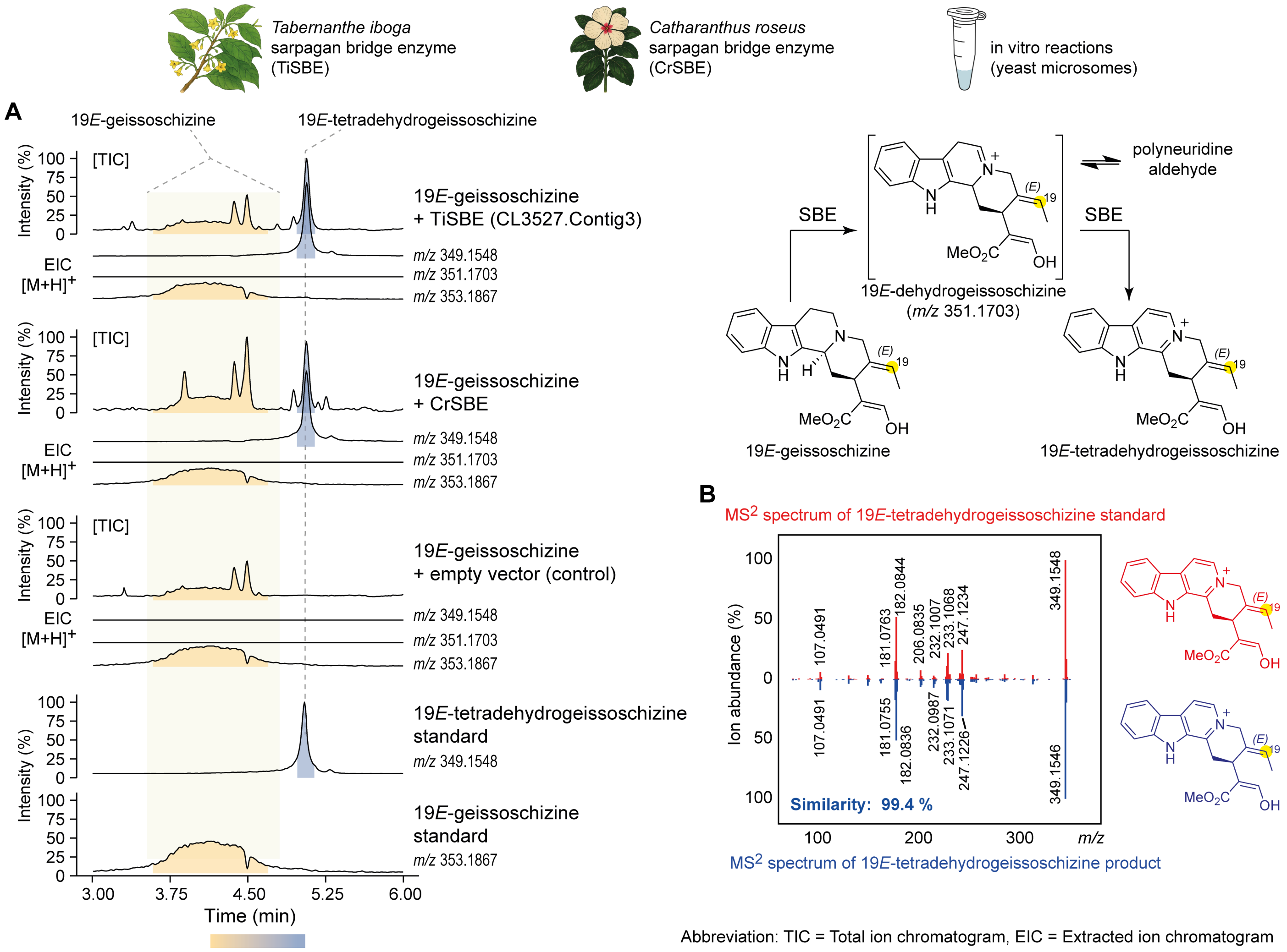

Figure S8. Functional characterization of *Tabernanthe iboga* sarpagan bridge enzyme (TiSBE) activity in vitro using 19*E*-geissoschizine. (**A**) LC-MS chromatograms of in vitro enzymatic assays using yeast microsomes expressing TiSBE and previously characterized *Catharanthus roseus* SBE (CrSBE) (5). Microsomal fractions were assayed with 19*E*-geissoschizine as substrate under identical conditions. CrSBE, previously characterized, was used as a positive control (5). Assays revealed that TiSBE catalyzed the formation of 19*E*-tetradehydrogeissoschizine via the unstable intermediate 19*E*-dehydrogeissoschizine, consistent with the activity observed for CrSBE. (**B**) MS^2 spectrum of 19*E*-tetradehydrogeissoschizine produced by TiSBE, compared with that of the authentic standard.

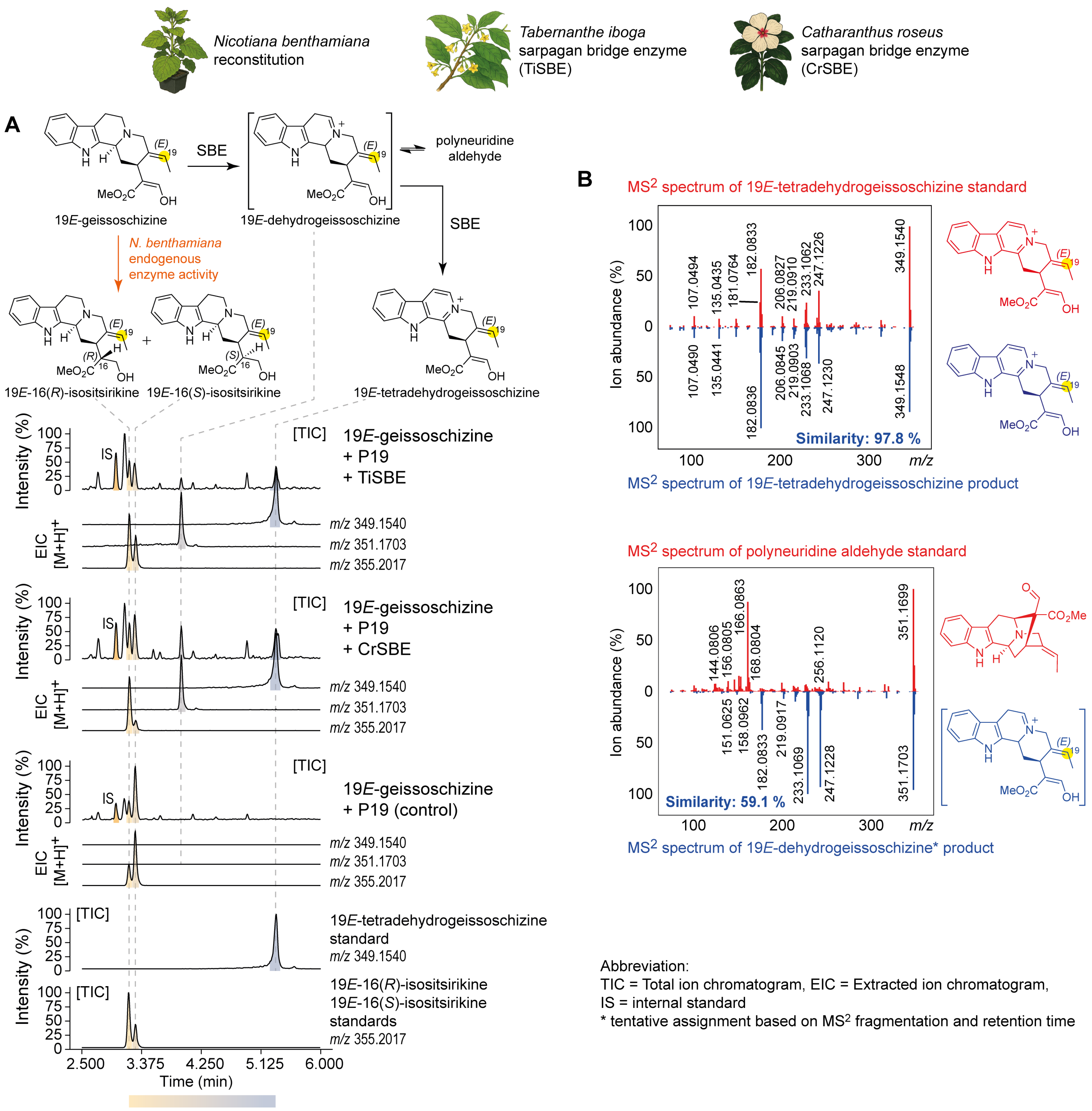

Figure S9. Functional characterization of *Tabernanthe iboga* sarpagan bridge enzyme (TiSBE) activity in *Nicotiana benthamiana* using 19*E*-geissoschizine as substrate. (**A**) LC-MS chromatograms of TiSBE transiently expressed in *N. benthamiana* with the P19 viral silencing suppressor, assayed in leaf-disks supplied with the substrate 19*E*-geissoschizine. The previously characterized *Catharanthus roseus* SBE (CrSBE) (5) was included as a positive control under identical conditions. Similar to CrSBE, TiSBE catalyzed the formation of 19*E*-tetradehydrogeissoschizine via the unstable intermediate 19*E*-dehydrogeissoschizine. The final product was confirmed using an authentic standard, while the intermediate was annotated based on its MS^2 fragmentation pattern. Notably, the endogenous metabolic activity of *N. benthamiana* resulted in the reduction of 19*E*-geissoschizine to 19*E*-isositsirikine isomers. (**B**) MS^2 spectrum of 19*E*-tetradehydrogeissoschizine produced by TiSBE, compared with the spectrum of the authentic standard. MS^2 spectrum of the 19*E*-dehydrogeissoschizine differs from that of polyneuridine aldehyde, which is known to exist in equilibrium with 19*E*-dehydrogeissoschizine, further supporting its structural annotation.

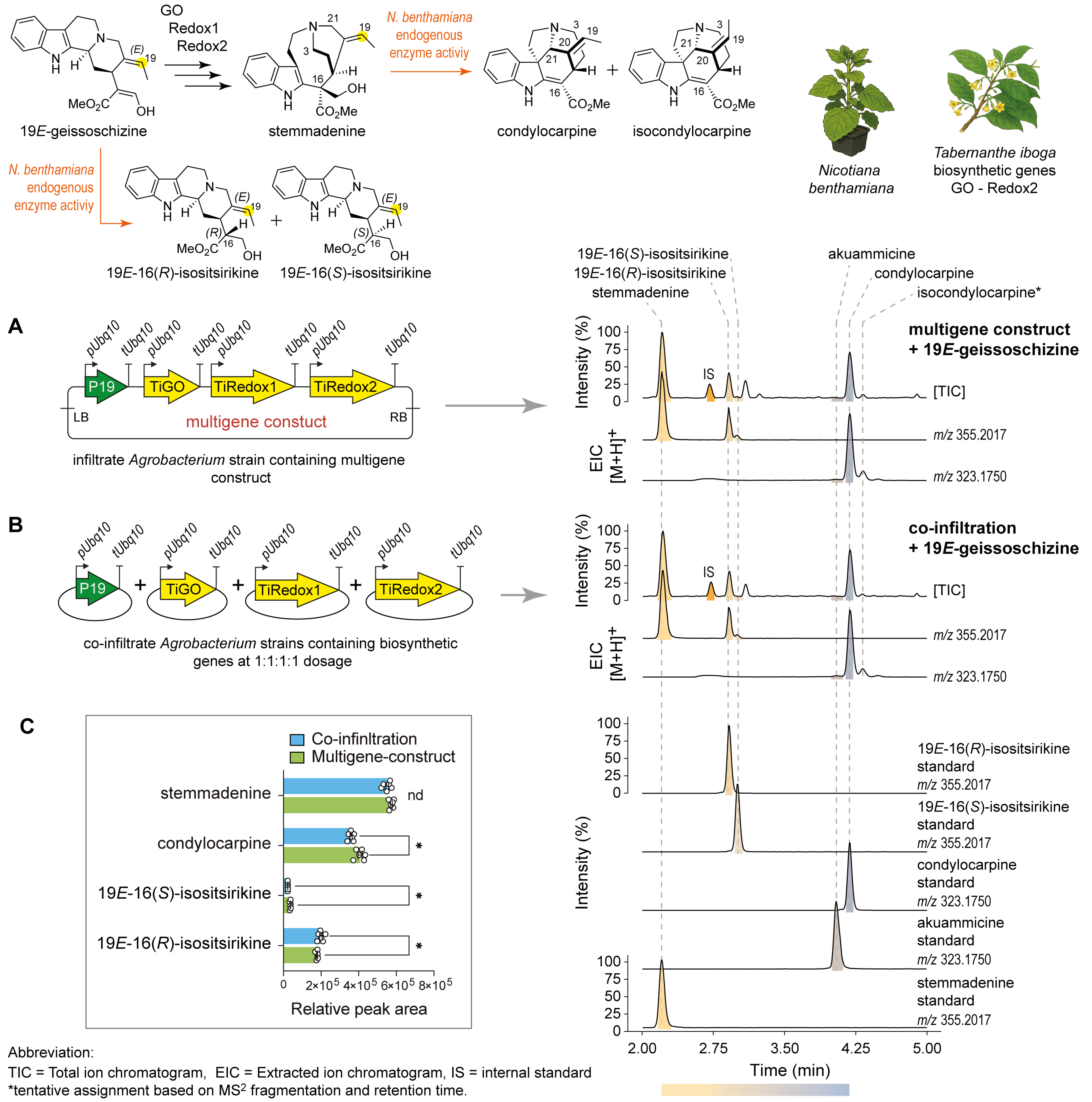

Abbreviation:
TIC = Total ion chromatogram, EIC = Extracted ion chromatogram, IS = internal standard
*tentative assignment based on MS[2] fragmentation and retention time.

Figure S10. Biosynthesis of stemmadenine in *Nicotiana benthamiana* using a multigene construct of *Tabernanthe iboga* biosynthetic genes. (**A**) A multigene construct comprising *T. iboga* biosynthetic genes GO, Redox1, and Redox2, along with the P19 viral silencing suppressor, enables the production of stemmadenine in *N. benthamiana* when the substrate 19*E*-geissoschizine is supplied exogenously. A Representative LC-MS chromatogram of the biosynthetic product, stemmadenine, is shown. (**B**) Co-infiltration of individual GO, Redox1, and Redox2 constructs with P19 in *N. benthamiana* also resulted in the production of stemmadenine, as shown in the representative LC-MS chromatogram. (**C**) Comparison of stemmadenine yields from multigene versus co-infiltration-based transient expression systems, using equivalent substrate concentrations. No statistically significant difference (nd) in stemmadenine production was observed between the two expression strategies (unpaired t-test, n = 6 biological replicates). However, the multigene construct produced statistically lower (*) levels of undesired side-products, resulting from endogenous *N. benthamiana* metabolic activity (unpaired t-test, *$P < 0.05$, nd = not significant $P > 0.05$). Relative peak area is normalized to the internal standard (IS). Bar graphs are plotted as the mean ± SD.

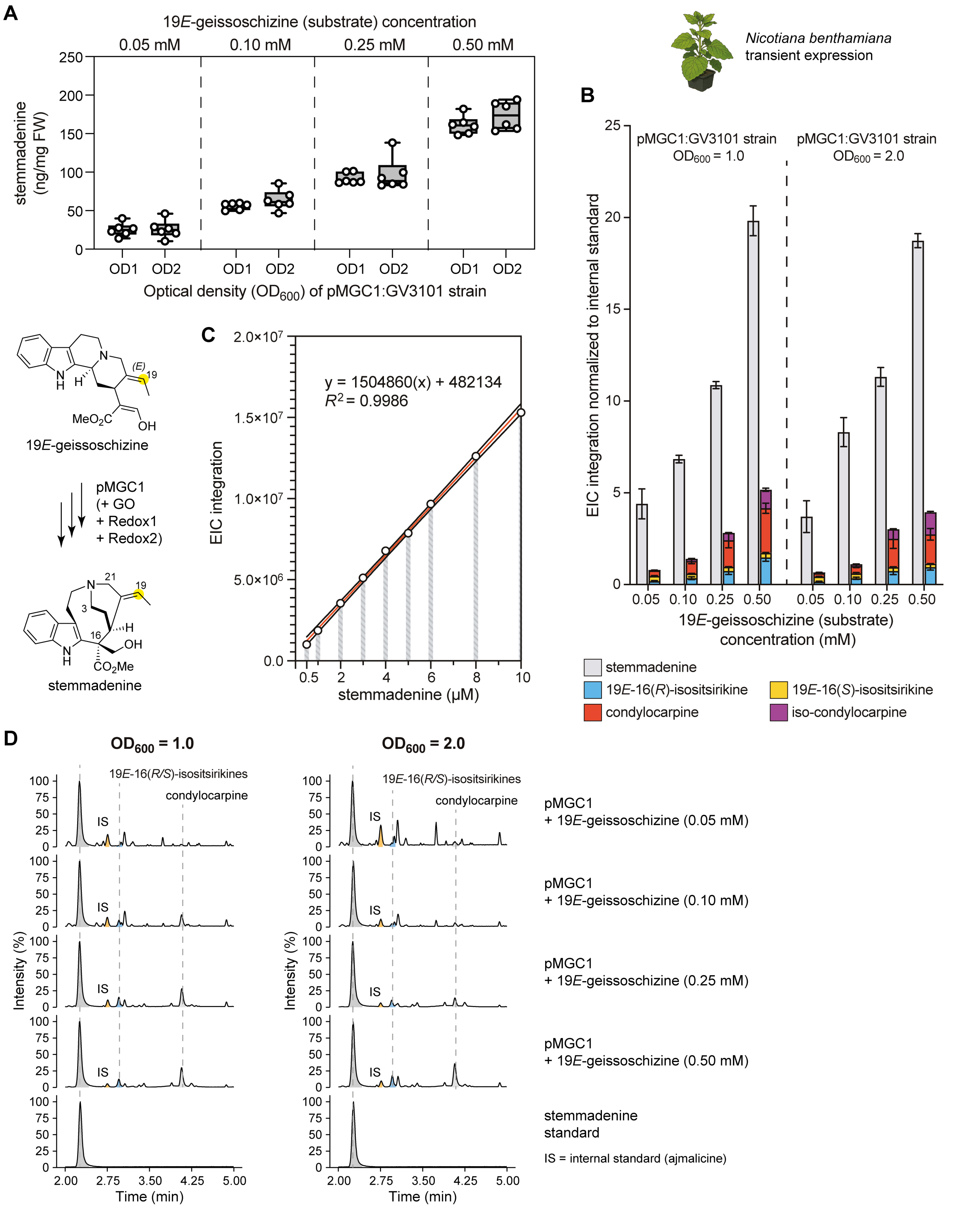

Figure S11. Quantification of stemmadenine production in *Nicotiana benthamiana* using a *Tabernanthe iboga* multigene construct with 19*E*-geissoschizine feeding. (A) Stemmadenine titers from leaves transiently expressing pMGC1 at *Agrobacterium* densities OD_{600} = 1.0 or 2.0, followed by exogenous 19*E*-geissoschizine at 0.05, 0.10, 0.25, or 0.50 mM. Tissue was harvested 12–16 h post-substrate infiltration (n = 6 biological replicates). (**B**) Qualitative product profiles (stemmadenine and co-produced MIAs) normalized to internal standard and dilution factor. (**C**) Calibration curve for stemmadenine quantification; best-fit line (red) with 95% confidence interval (black). (**D**) Representative LC–MS chromatograms illustrating product profile under the conditions in (A).

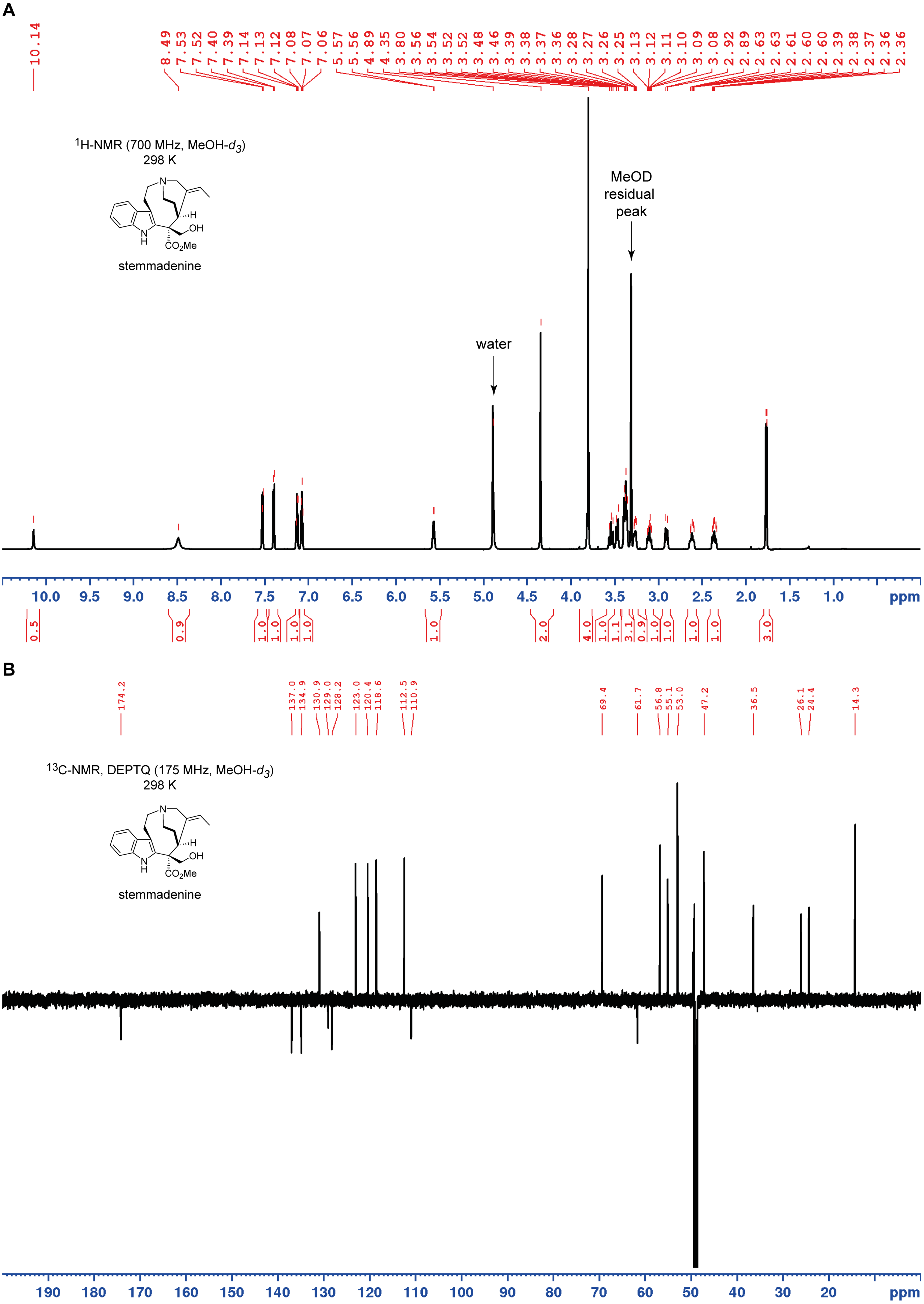

Figure S12. NMR of stemmadenine. (A) ^{1}H NMR with water presaturation full range in MeOH-d_3 of stemmadenine as formate. (B) DEPTQ full range in MeOH-d_3 of stemmadenine as formate.

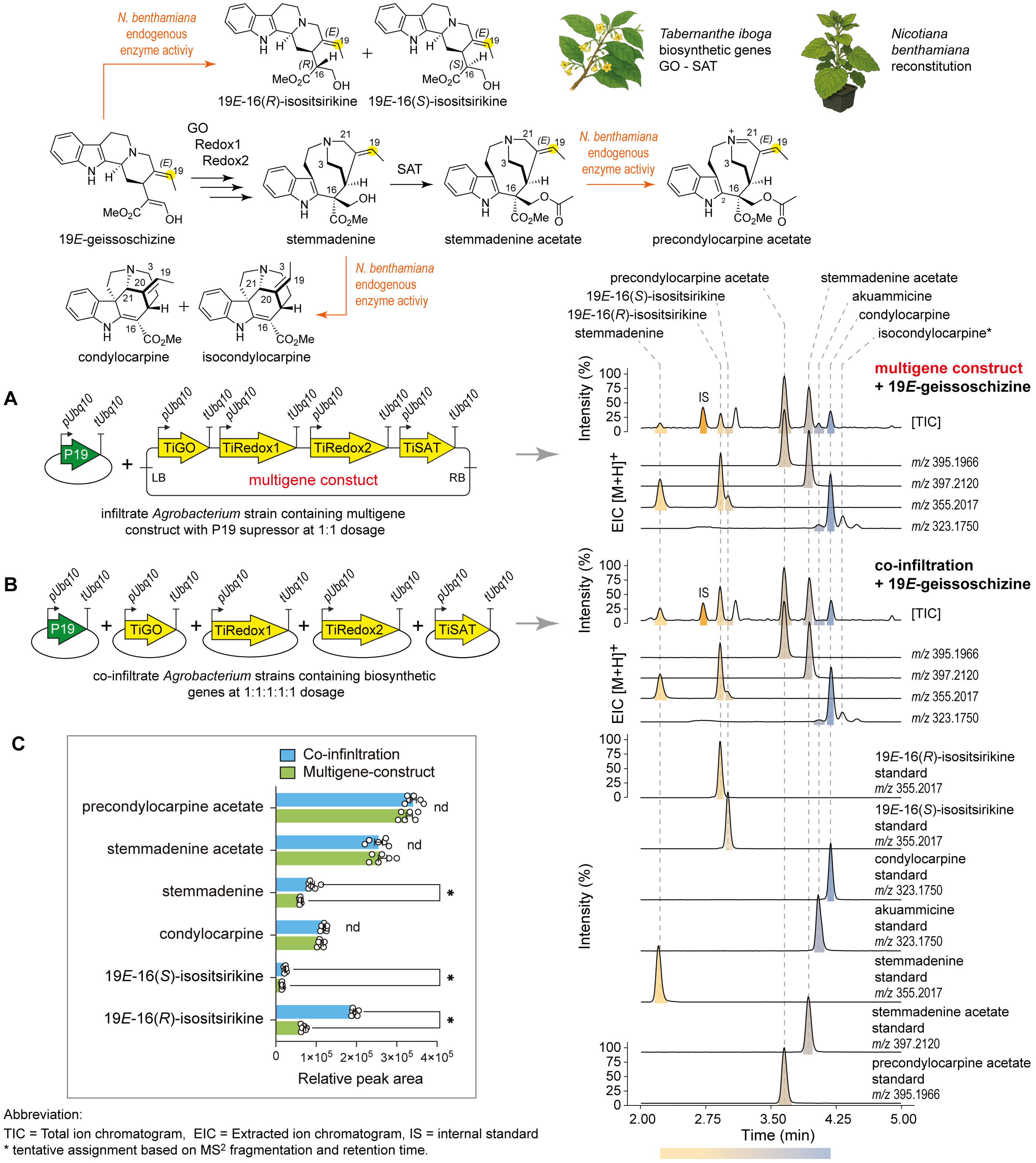

Abbreviation:

TIC = Total ion chromatogram, EIC = Extracted ion chromatogram, IS = internal standard
* tentative assignment based on MS^2 fragmentation and retention time.

Figure S13. Biosynthesis of stemmadenine acetate in *Nicotiana benthamiana* using a multigene construct of *Tabernanthe iboga* biosynthetic genes. (**A**) A multigene construct comprising *T. iboga* biosynthetic genes GO, Redox1, Redox2, and SAT, co-infiltrated with P19 viral silencing suppressor, enables the production of stemmadenine acetate in *N. benthamiana* upon exogenous supply of 19*E*-geissoschizine substrate. LC-MS chromatograms of the biosynthetic product, along with side-products, are shown. In *N. benthamiana*, stemmadenine acetate is readily oxidized to precondylocarpine acetate by endogenous metabolic activity. (**B**) Co-infiltration of individual GO, Redox1, Redox2, and SAT constructs with P19 also resulted in stemmadenine acetate production, as shown in the corresponding LC-MS chromatogram. (**C**) Comparison of stemmadenine acetate yields between the multigene and co-infiltration-based transient expression strategies, using equivalent substrate concentrations. No statistical difference (nd) was observed in stemmadenine acetate production between the two approaches (unpaired t-test, $n = 6$ biological replicates). However, the multigene construct produced statistically lower (*) levels of undesired side-products, arising from endogenous *N. benthamiana* metabolic activity (unpaired *t-test*, $^*P < 0.05$, nd = not significant, $P > 0.05$, n = 6 biological replicates). Relative peak area is normalized to the internal standard (IS). Bar graphs are plotted as the mean ± SD.

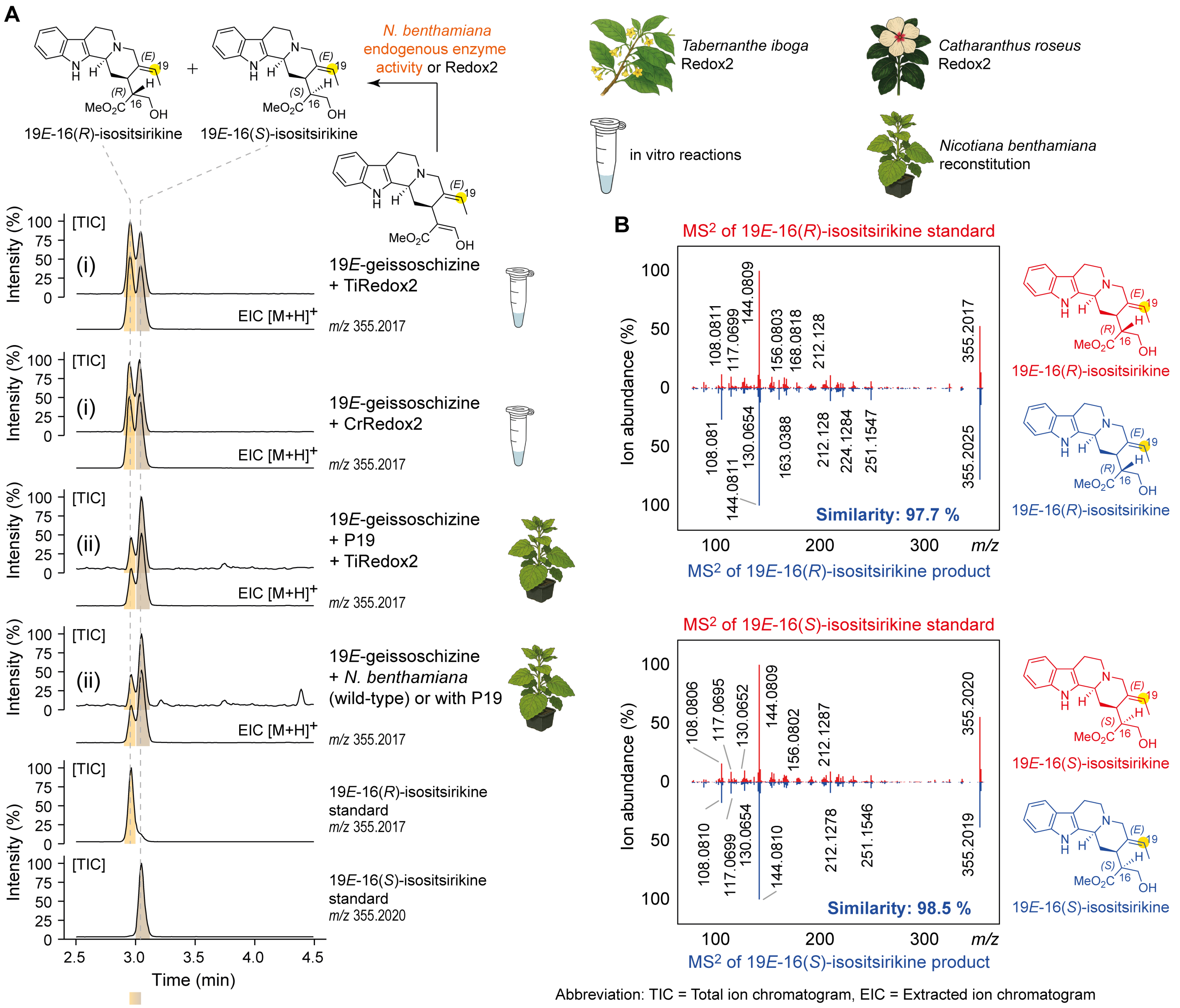

Figure S14. Functional characterization of *Tabernanthe iboga* redox enzyme 2 (TiRedox2) activity using 19*E*-geissoschizine. (**A**) (i) LC-MS chromatograms of in vitro enzymatic assays using heterologously expressed TiRedox2 and *Catharanthus roseus* (CrRedox2), assayed with the substrate 19*E*-geissoschizine. Both TiRedox2 and CrRedox2 catalyzed the reduction of 19*E*-geissoschizine to the isomeric products 19*E*-16(*R*)-isositsirikine and 19*E*-16(*S*)-isositsirikine. The absolute structure of 19*E*-16(*R*)-isositsirikine and 19*E*-16(*S*)-isositsirikine was confirmed after purification and NMR analysis. (ii) LC-MS chromatograms showing that endogenous *Nicotiana benthamiana* metabolic activity also catalyzes the reduction of 19*E*-geissoschizine to the identical 19*E*-isositsirikine isomers. (**B**) MS^2 spectra of 19*E*-16(*R*)-isositsirikine and 19*E*-16(*S*)-isositsirikine isomers generated by TiRedox2, shown alongside spectra of authentic synthetic standards.

A

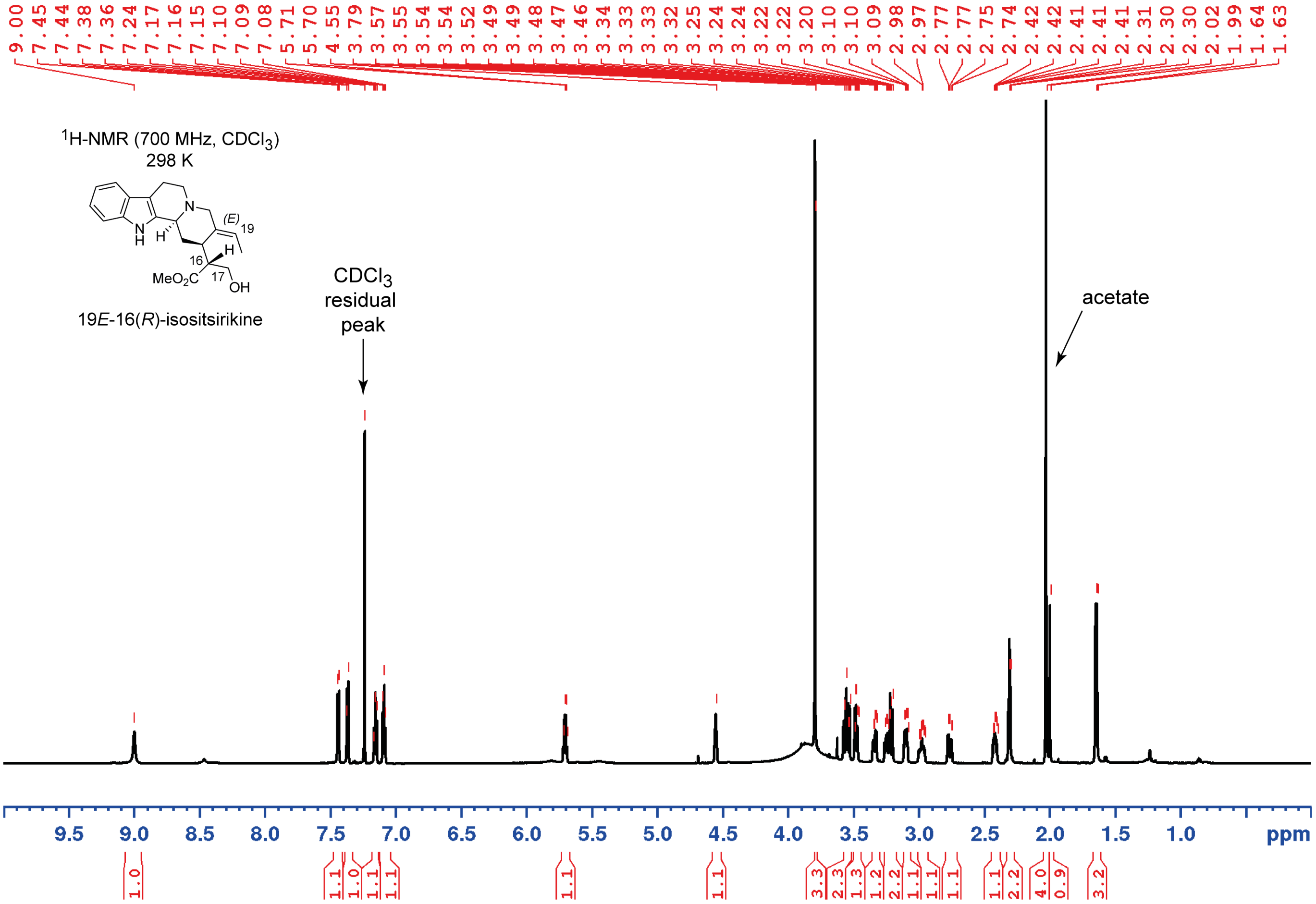

B

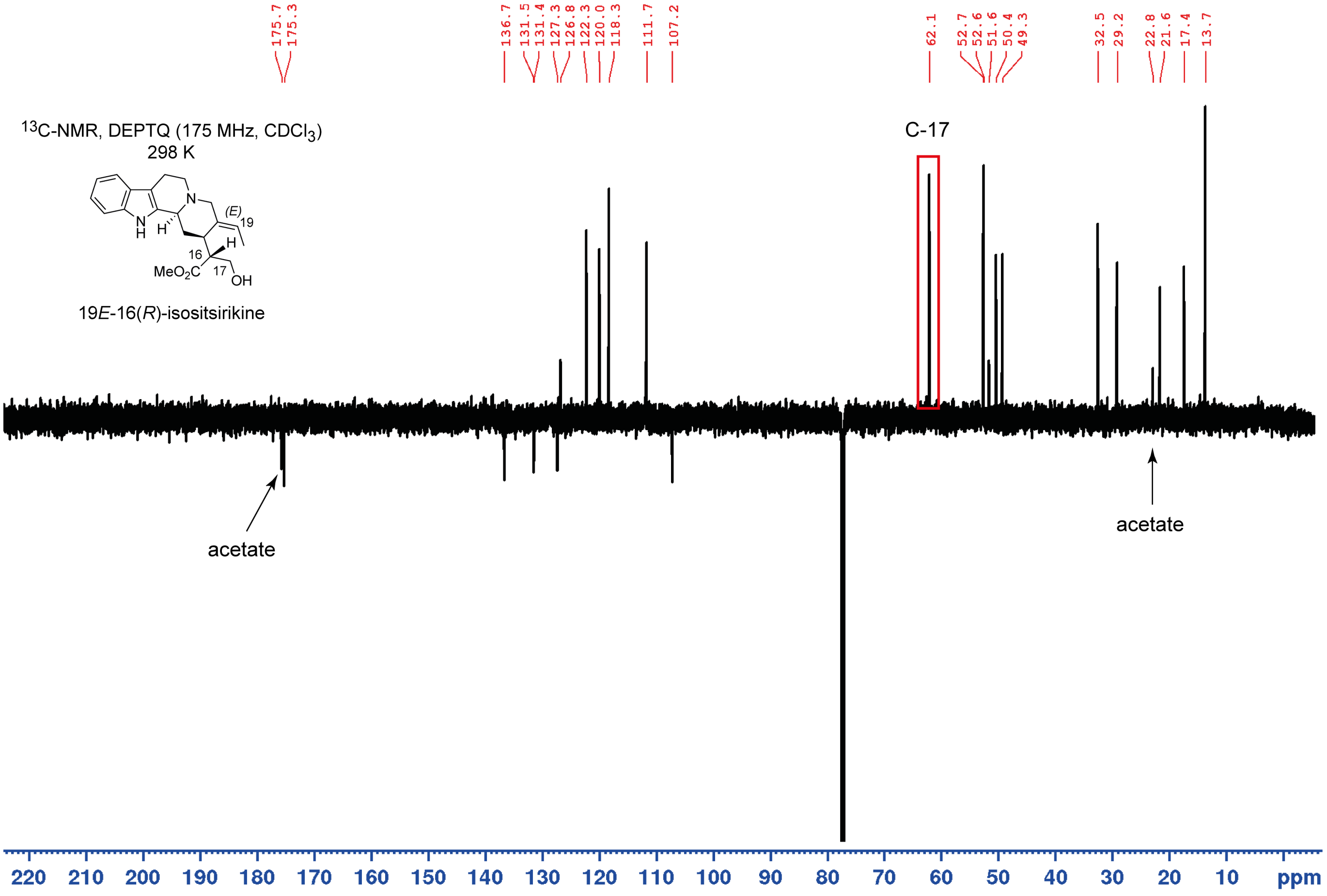

Figure S15. NMR of 19*E*-16(*R*)-isositsirikine. (**A**) ^{1}H NMR with water presaturation, full range in $CDCl_3$ of 19*E*-16(*R*)-isositsirikine as acetate. (**B**) DEPTQ full range in $CDCl_3$ of 19*E*-16(*R*)-isositsirikine as acetate.

A

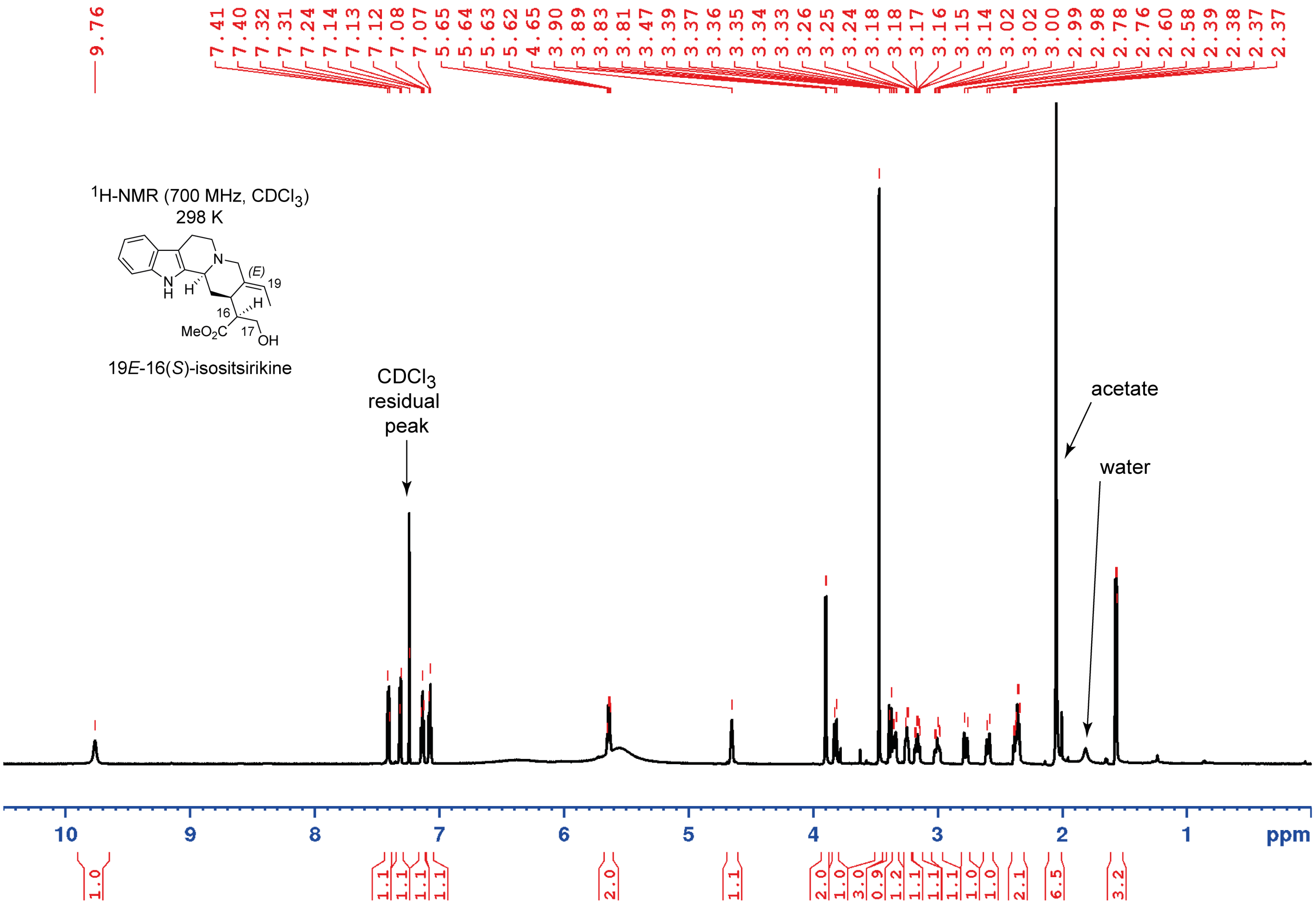

B

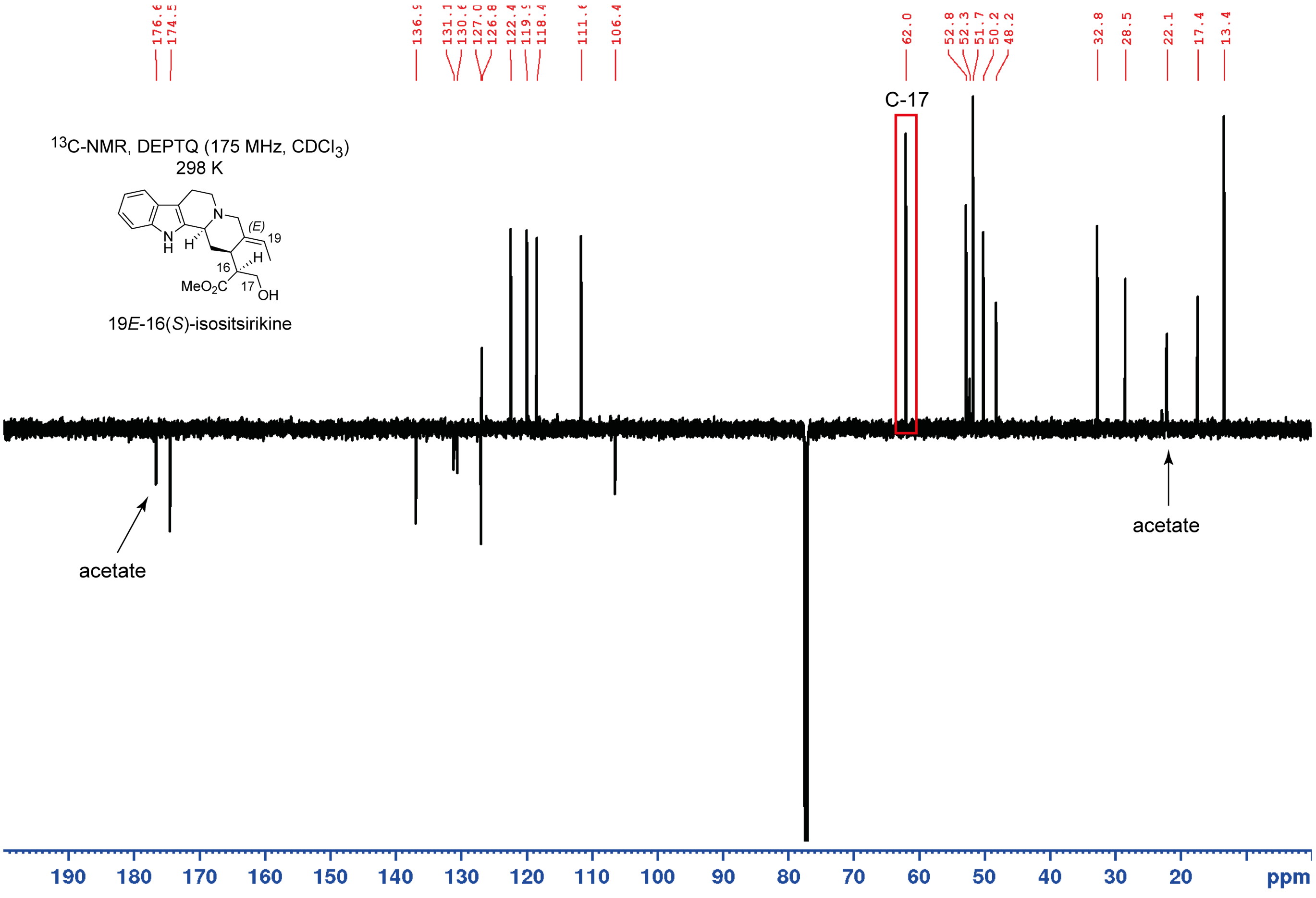

Figure S16. NMR of 19*E*-16(*S*)-isositsirikine. (**A**) ^{1}H NMR with water presaturation, full range in $CDCl_3$ of 19*E*-16(*S*)-isositsirikine as acetate. (**B**) DEPTQ full range in $CDCl_3$ of 19*E*-16(*S*)-isositsirikine as acetate.

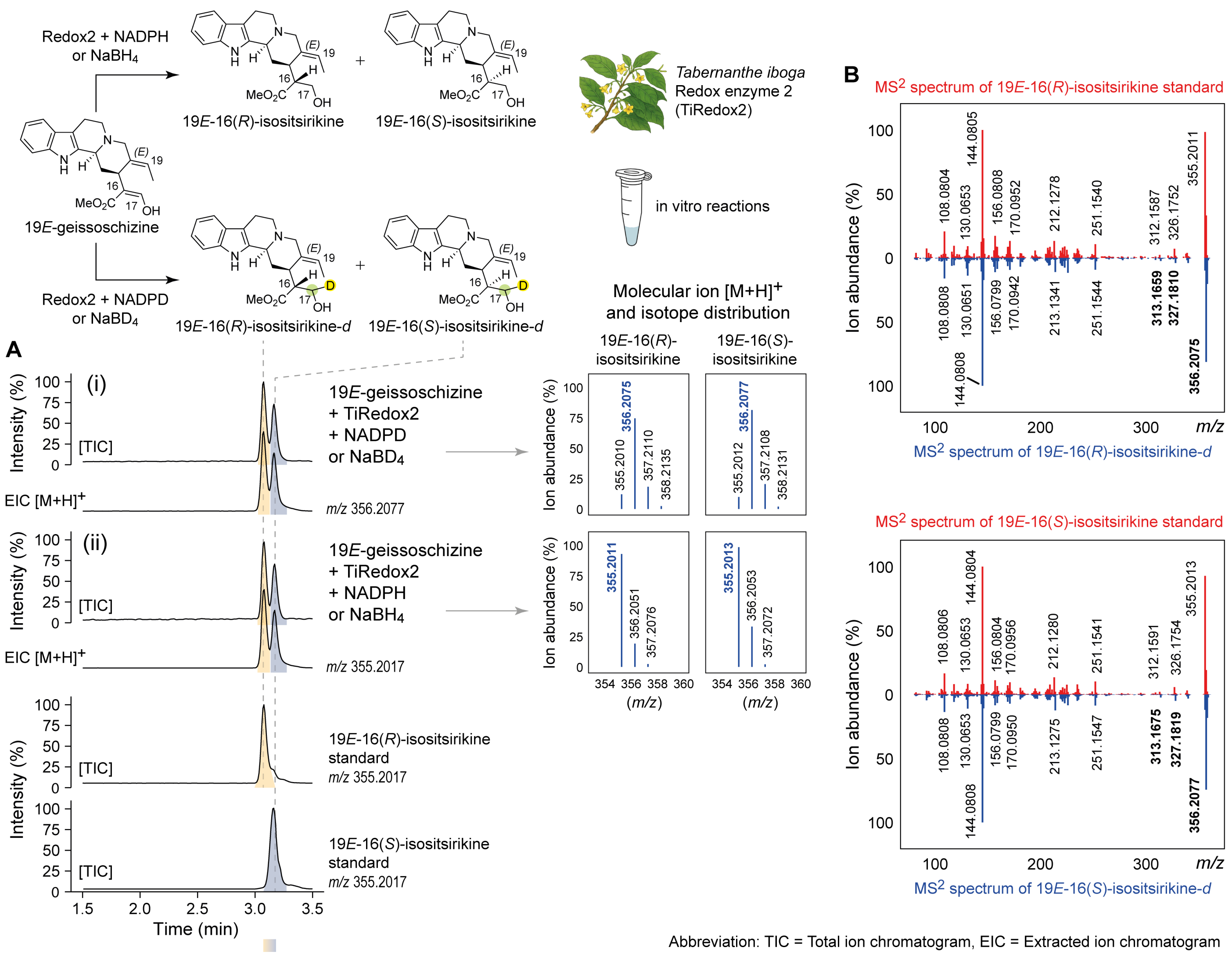

Figure S17. Deuterium-labeled (-*d*) reduction assays of 19*E*-geissoschizine. (**A**) (i) LC-MS chromatograms and the molecular ion mass spectra of 19*E*-16(*R*)-isositsirikine-*d* and 19*E*-16(*S*)-isositsirikine-*d* isomers generated by the chemical reduction of 19*E*-geissoschizine using $NaBD_4$ or by enzymatic reduction with TiRedox2 in the presence of isotopically labeled NADPH (NADPD). (ii) LC-MS chromatograms and the molecular ion mass spectra of the corresponding unlabeled 19*E*-16(*R*)-isositsirikine and 19*E*-16(*S*)-isositsirikine isomers, generated by the chemical reduction of 19*E*-geissoschizine using $NaBH_4$ or enzymatic assays using TiRedox2 with NADPH. (**B**) MS^2 spectra of the deuterium labeled isomers 19*E*-16(*R*)-isositsirikine-*d* and 19*E*-16(*S*)-isositsirikine-*d* compared to those of the authentic 19*E*-isomers standards, highlighting the incorporation of deuterium into each isomer and its corresponding fragment.

A

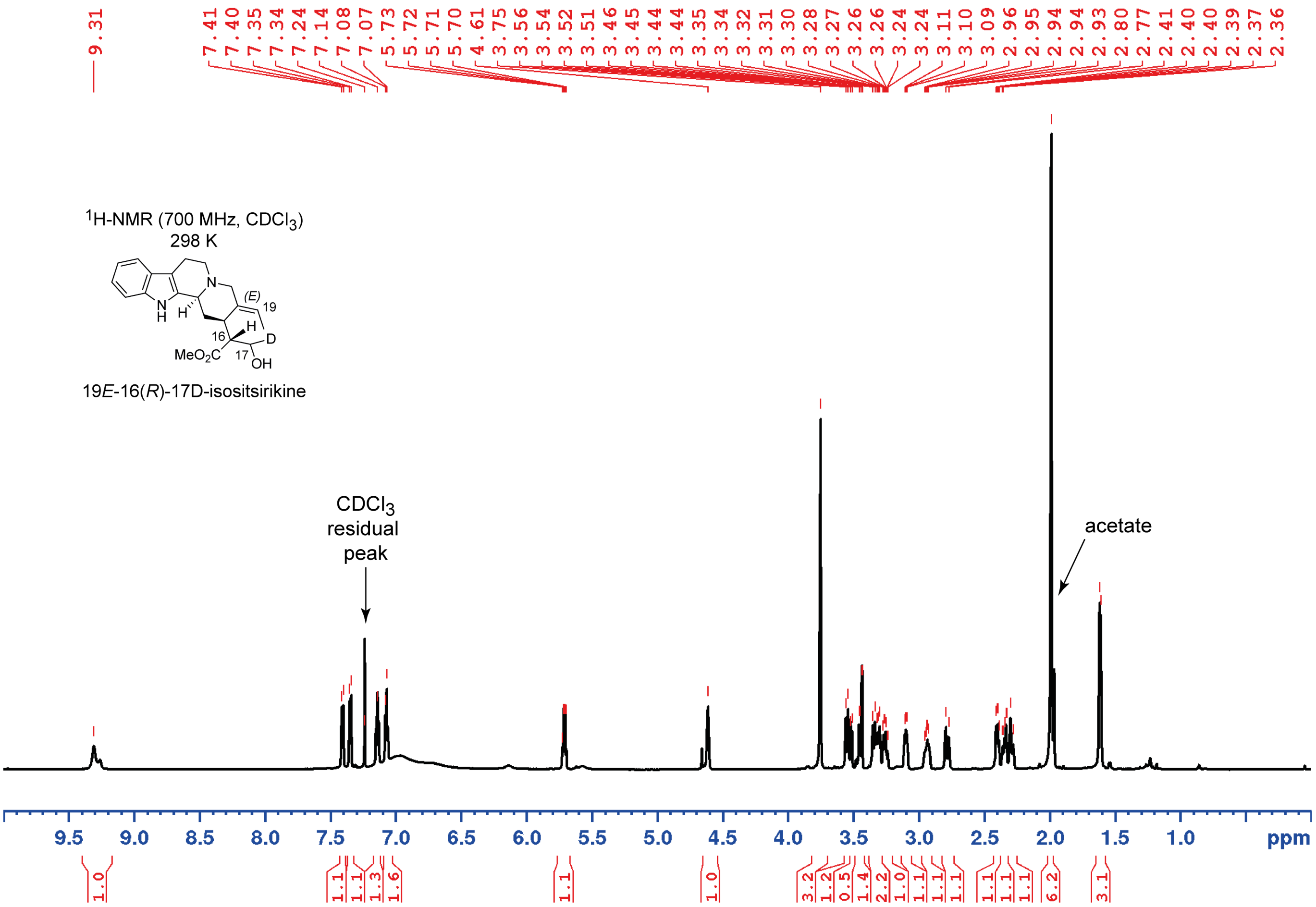

B

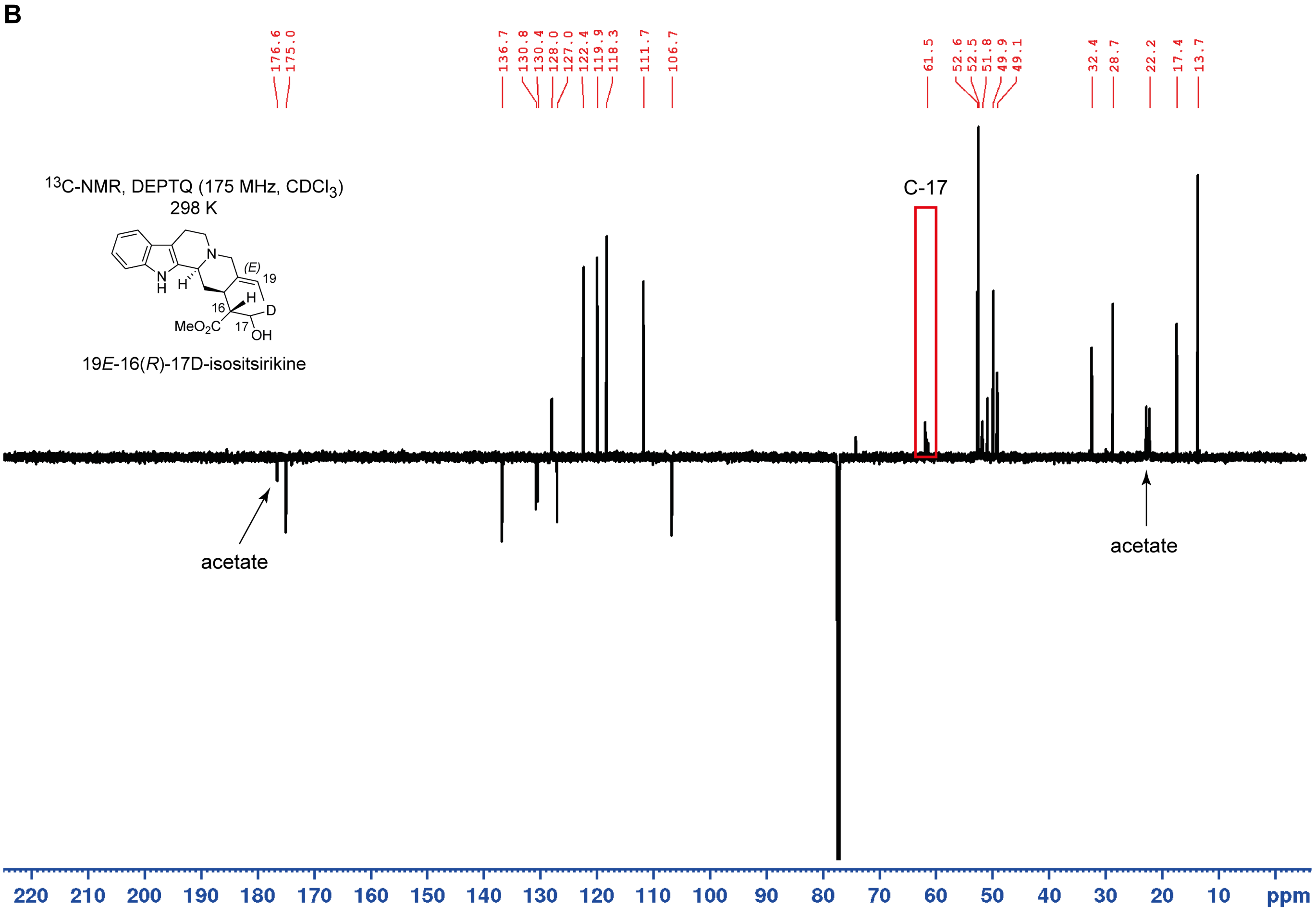

Figure S18. NMR of 19*E*-16(*R*)-17D-isositsirikine. (**A**) ^{1}H NMR with water presaturation, full range in $CDCl_3$ of 19*E*-16(*R*)-17D-isositsirikine as acetate. (**B**) DEPTQ full range in $CDCl_3$ of 19*E*-16(*R*)-17D-isositsirikine as acetate.

A

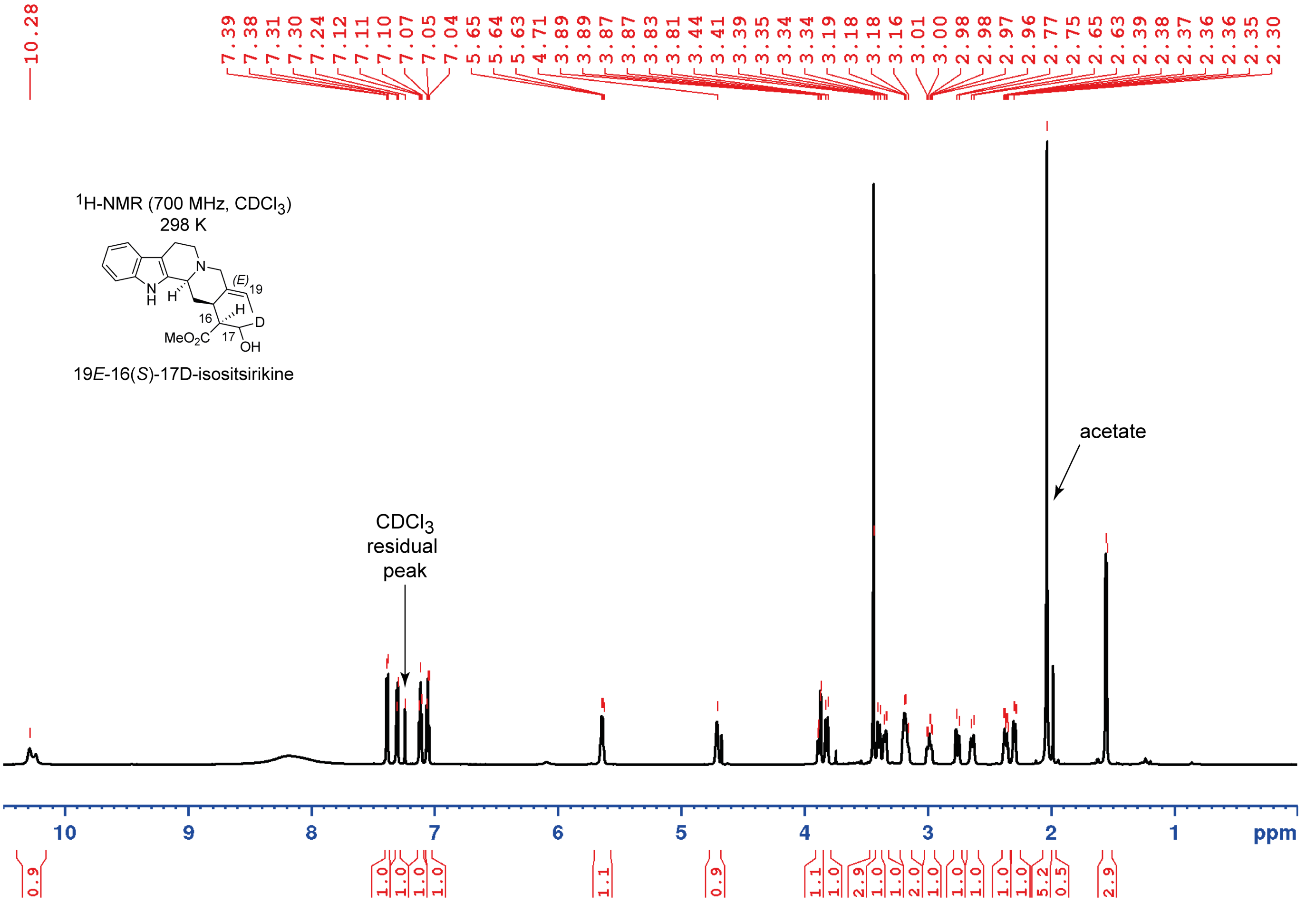

B

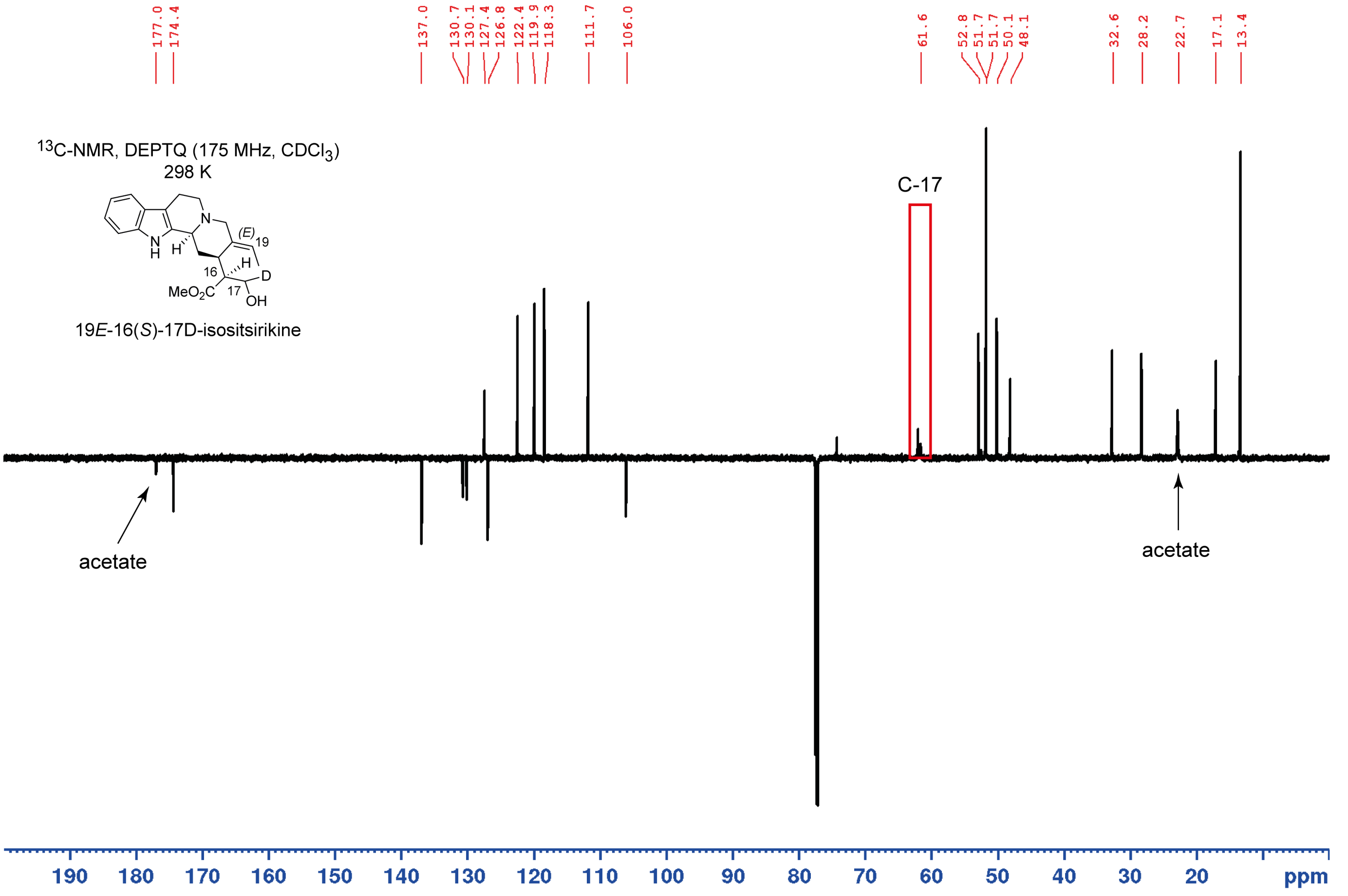

Figure S19. NMR of 19*E*-16(*S*)-17D-isositsirikine. (**A**) ^{1}H NMR with water presaturation, full range in $CDCl_3$ of 19*E*-16(*S*)-17D-isositsirikine as acetate. (**B**) DEPTQ full range in $CDCl_3$ of 19*E*-16(*S*)-17D-isositsirikine as acetate.

A

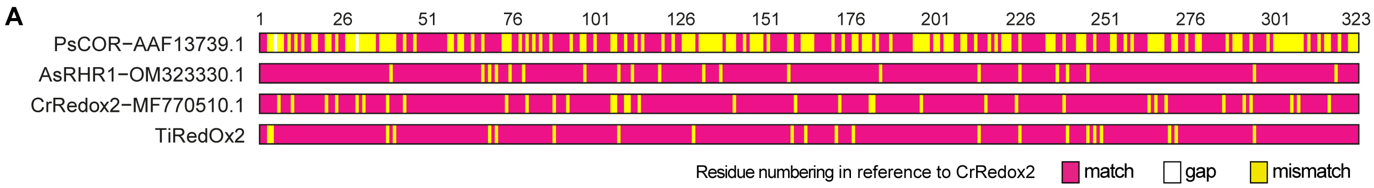

B

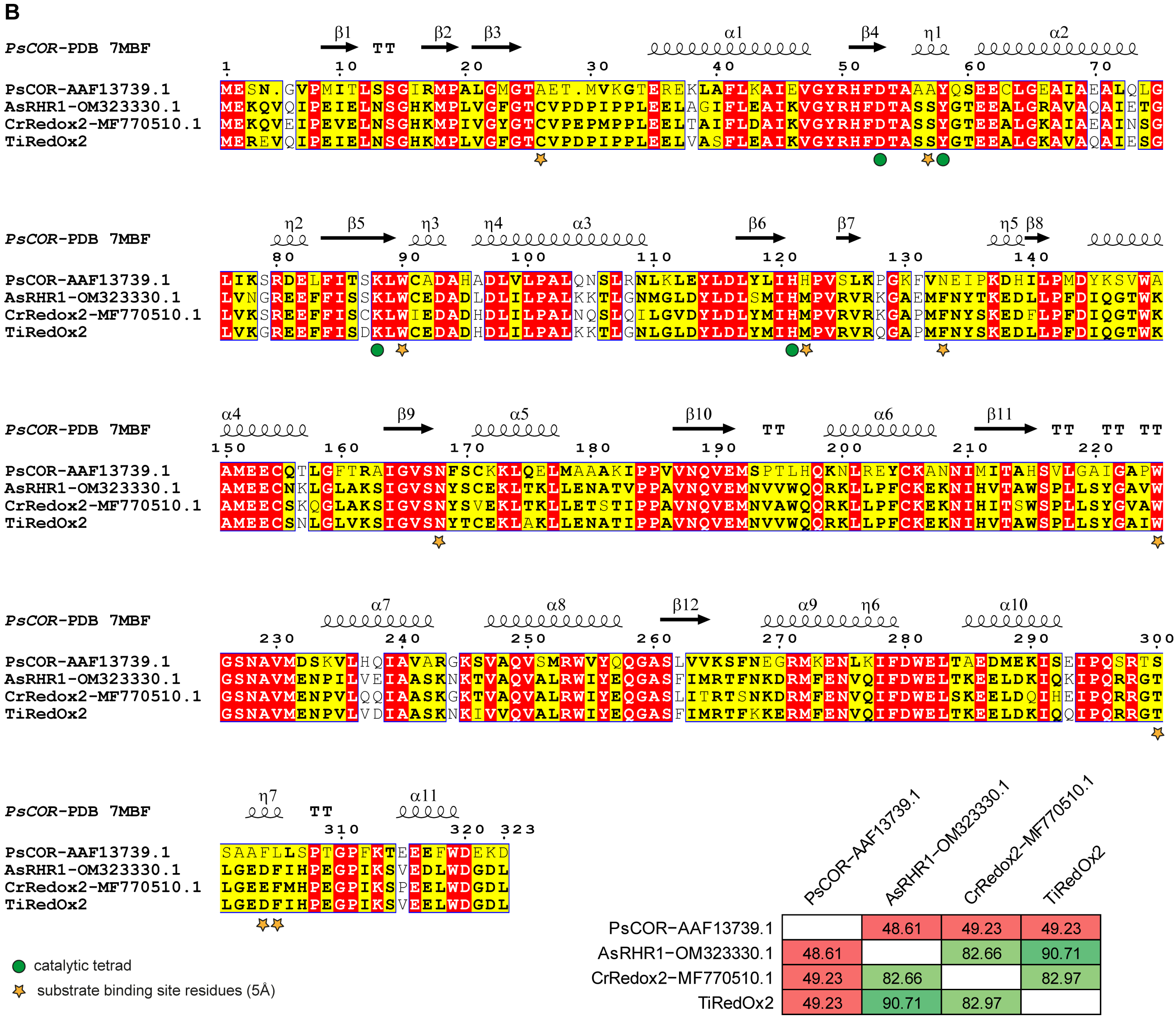

	PsCOR−AAF13739.1	AsRHR1−OM323330.1	CrRedox2−MF770510.1	TiRedOx2
PsCOR−AAF13739.1		48.61	49.23	49.23
AsRHR1−OM323330.1	48.61		82.66	90.71
CrRedox2−MF770510.1	49.23	82.66		82.97
TiRedOx2	49.23	90.71	82.97	

Figure S20. Multiple sequence alignment of aldo-keto reductase (AKR) enzymes involved in plant alkaloid biosynthesis. (**A**) Schematic representation of amino acid sequence alignment of functionally characterized AKR enzymes, the enzyme family to which Redox2 belongs in the stemmadenine biosynthetic pathway. (**B**) Detailed alignment of *T. iboga* Redox2 with orthologous AKR enzymes involved in monoterpene indole alkaloid (MIA) biosynthesis (3, 6), annotated with structural features based on the closest structural homolog, *Papaver somniferum* codeinone reductase (PsCOR) (7). Key residues comprising the catalytic tetrad and those involved in substrate binding pocket interactions are highlighted (8). The percentage amino acid identity between TiRedox2 and the other plant AKR enzymes is also shown. GenBank accession numbers for all functionally characterized AKR enzymes are provided as appropriate.

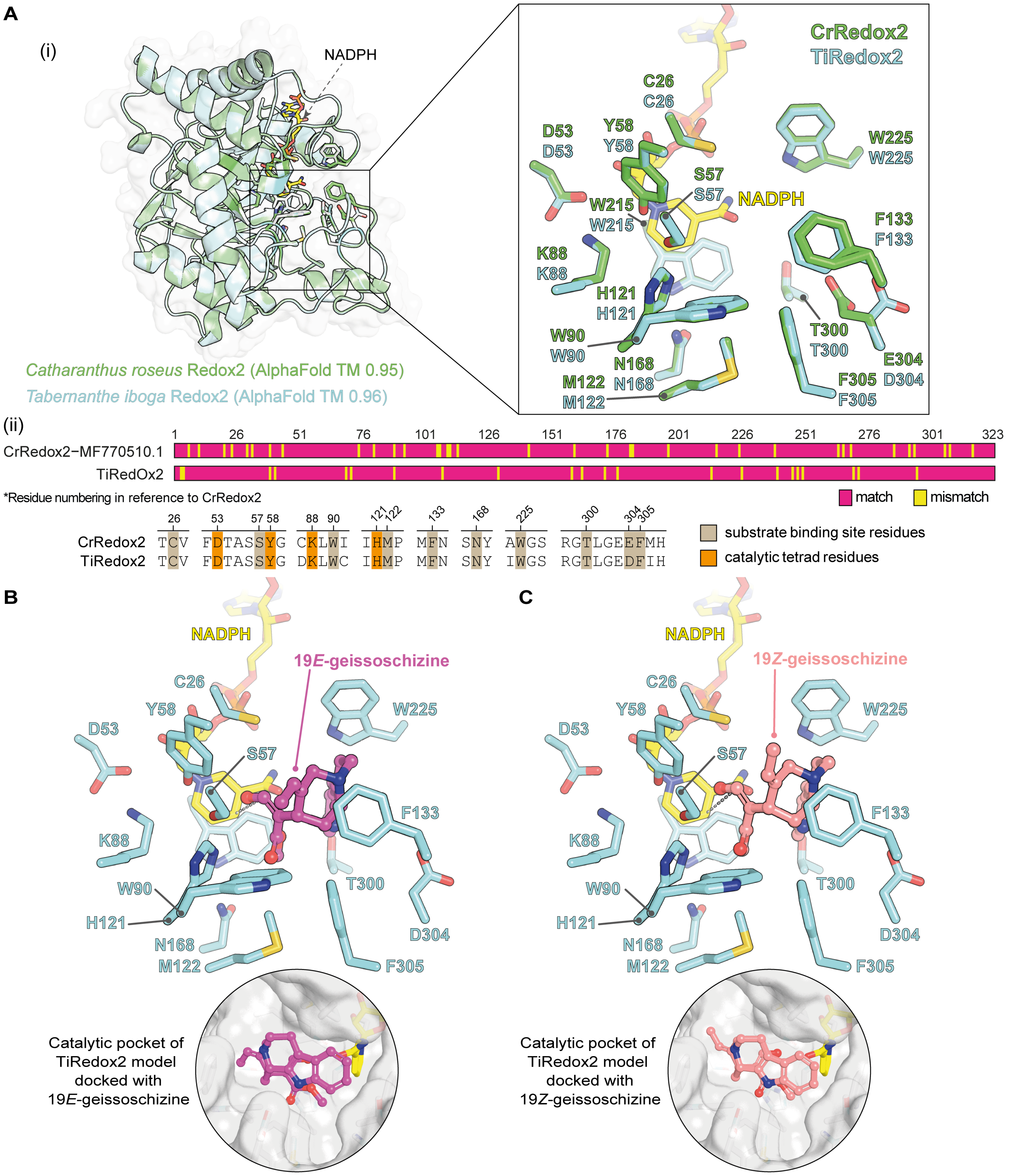

Figure S21. Structural analysis of *Tabernanthe iboga* redox enzyme 2 (TiRedox2) model. (**A**) (**i**) Superimposed AlphaFold models of TiRedox2 (TM-score = 0.9514) and *Catharanthus roseus* Redox2 (CrRedox2) (TM score = 0.9634), generated using AlphaFold v3 (9) with NADPH modeled in the cofactor pocket. The conserved catalytic tetrad characteristic of the aldo-keto reductase (AKR) family enzymes, along with residues located within 5 Å of the active site pocket, are visualized in complex with NADPH. (**ii**) Schematic representation of the amino acid sequence alignment between TiRedox2 and CrRedox2 (82.97% identity), highlighting conserved and divergent regions. The catalytic tetrad (Asp53, Tyr58, Lys88, and His121) (8) and putative substrate-binding residues are indicated. (**B**) Molecular docking of the substrate 19*E*-geissoschizine into the TiRedox2 model, illustrating interactions between the substrate and active site residues. The carbonyl (aldehyde) moiety of 19*E*-geissoschizine (substrate) is positioned adjacent to the catalytic residues and NADPH, consistent with a reductive mechanism for AKRs. (**C**) Molecular docking of the substrate 19*Z*-geissoschizine into the TiRedox2 model reveals a binding orientation comparable to the 19*E*-isomer. The *Z*-configuration of the ethyl moiety at C20 does not significantly alter the substrate orientation within the active site, suggesting a similar reduction mechanism. Template modelling (TM) scores reflect the overall confidence of the AlphaFold v3 (9) model. Molecular docking was performed using AutoDock Vina (10) with default settings; the best-ranked pose (lowest predicted binding energy and optimal orientation of interaction residues) was selected for analysis.

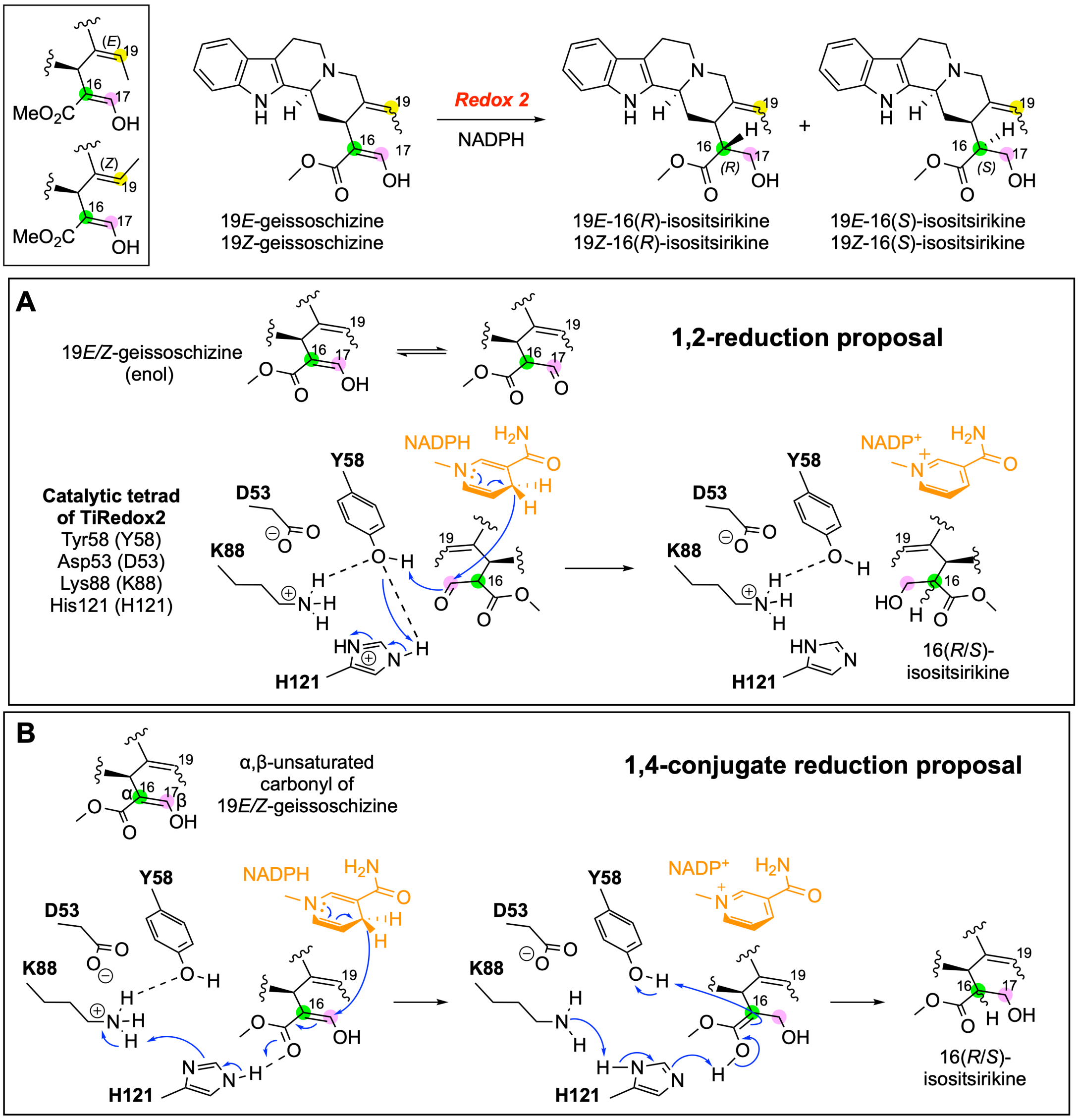

Figure S22. Mechanistic proposal of 19*E/Z*-geissoschizine reduction catalyzed by Redox2. *Tabernanthe iboga* aldo-keto reductase (AKR) enzyme Redox2 (TiRedox2) catalyzes the NADPH-dependent reduction of 19*E/Z*-geissoschizine at C17, to yield the diastereomeric products 16(*R*)- and 16(*S*)- isositsirikine. (**A**) The reaction proceeds via the canonical "push-pull" reduction mechanism typical of AKRs (8, 11, 12). The conserved catalytic tetrad of TiRedox2, Tyr58, Asp53, Lys88, and His122 plays a crucial role in catalysis to facilitate the push-pull reaction mechanism. The NADPH cofactor binds to the enzyme, followed by a 4-pro-*R* hydride transfer to the substrate aldehyde and protonation of the oxygen by Tyr58 which acts as general acid by participating in the proton relay with His121, completing the 1,2-reduction to form a hydroxyl group. Lys88 hydrogen-bonds to Tyr58 to tune its *pKa* and enforce geometry, whereas Asp53 forms a salt bridge with Lys88 to modulate overall electrostatics and facilitate proton donation. (**B**) The spatial conservation of the active-site tetrad allows the possibility for an alternative 1,4-reduction mechanism, which cannot be ruled out based on the hydride transfer to C17. In this case, the substrate ester (enol) forms a hydrogen bond with His121, which in turn acts as a general acid and proton donor upon hydride transfer by NADPH. Dashed lines indicate hydrogen bonds.

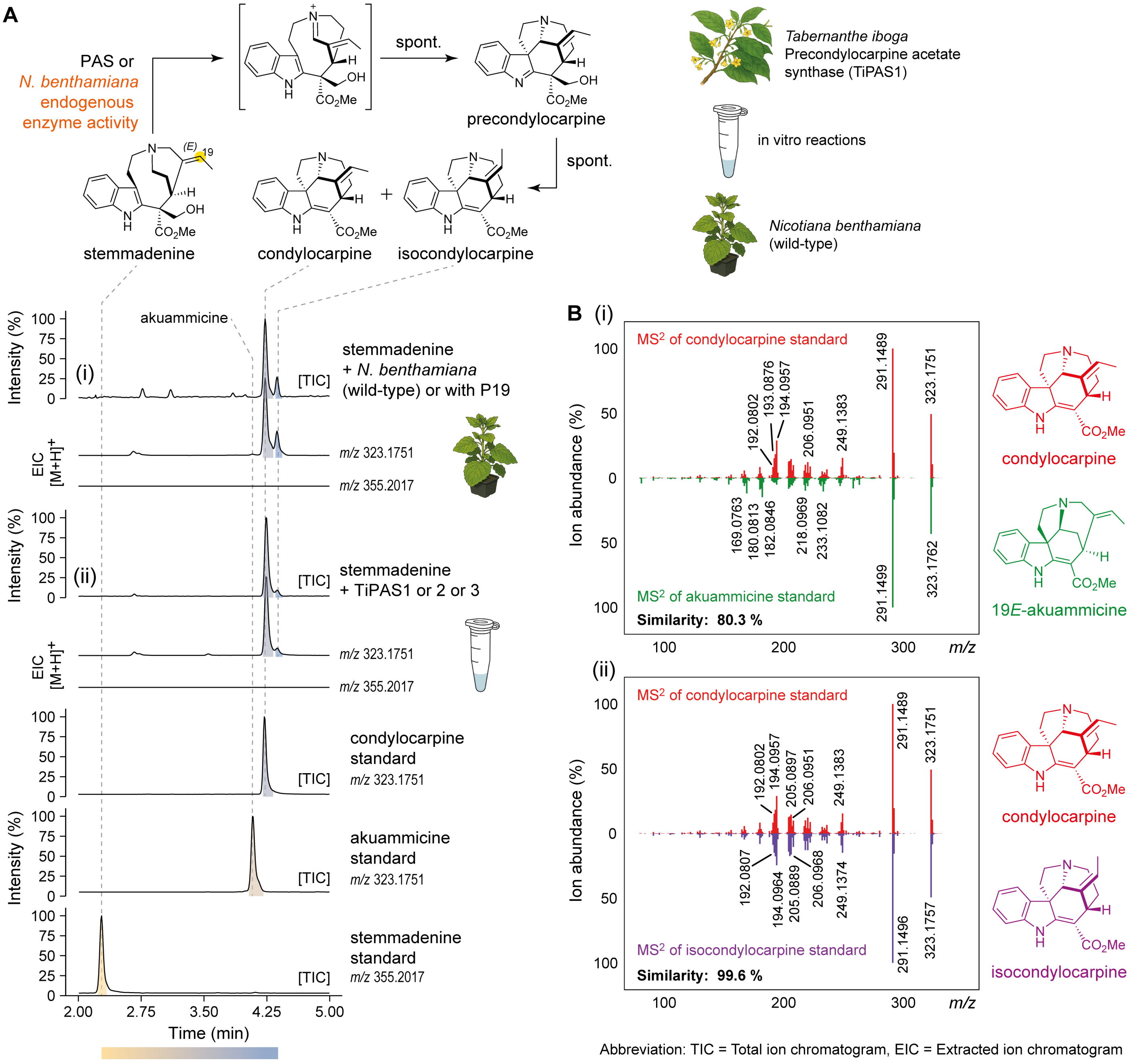

Figure S23. Biosynthesis of condylocarpine and isocondylocarpine in *Nicotiana benthamiana*. (**A**) (i) LC-MS chromatograms showing endogenous *N. benthamiana* metabolic activity resulting in the oxidation of stemmadenine to form condylocarpine and a putative isomer, isocondylocarpine. When stemmadenine is present in *N. benthamiana* either as a product of pathway reconstitution or following exogenous feeding, it is progressively oxidized to these products over time. (ii) LC-MS chromatograms of in vitro enzymatic assays with heterologously expressed *Tabernanthe iboga* precondylocarpine acetate synthase homologs (TiPAS1-3), incubated with stemmadenine. TiPAS homologs can catalyze the oxidation of stemmadenine to yield condylocarpine and isocondylocarpine. This suggests that the oxidation observed in *N. benthamiana* host may be mediated by berberine bridge enzyme-like (BBL) oxidases, which belong to the same family as TiPAS (13). The structure of condylocarpine was confirmed in this study by purification and NMR analysis, while the minor product isocondylocarpine was tentatively annotated based on its MS^2 fragmentation spectrum and retention time. (**B**) (i) MS^2 spectral comparison of condylocarpine and akuammicine, both metabolic sink products of the same *m/z* 323.1751 $(M+H)^+$ due to the absence of downstream biosynthetic enzymes. Although these compounds exhibit similar retention times and MS^2 fragment ions, close inspection of their MS^2 spectra reveals unique diagnostic fragments for condylocarpine. (ii) MS^2 spectral comparison between condylocarpine and isocondylocarpine shows high similarity in their fragmentation patterns, supporting the structural assignment of isocondylocarpine as a stereoisomer of condylocarpine.

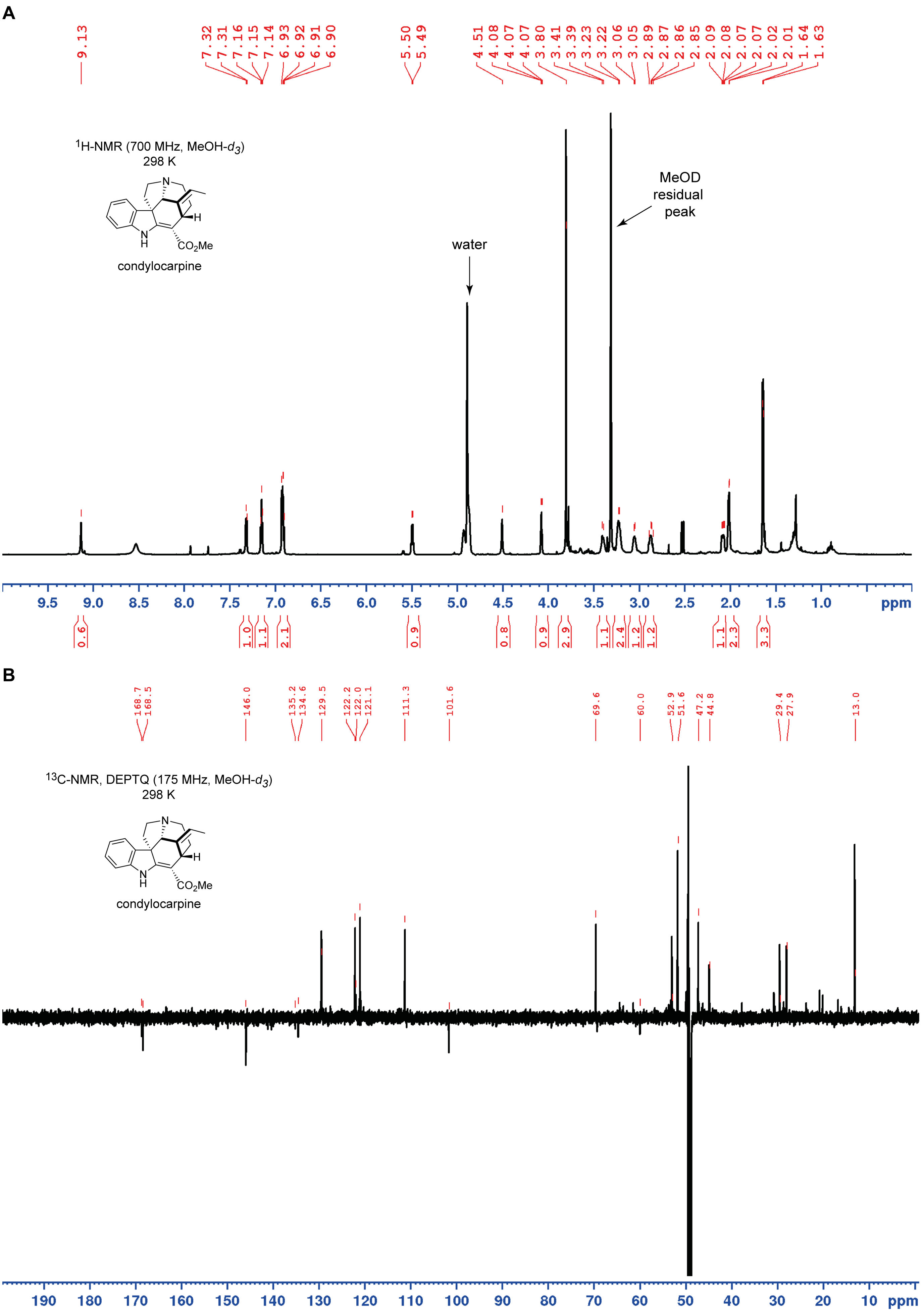

Figure S24. NMR of condylocarpine. (**A**) 1H NMR with water presaturation full range in MeOH-d_3 of condylocarpine as formate. (**B**) DEPTQ full range in MeOH-d_3 of condylocarpine as formate.

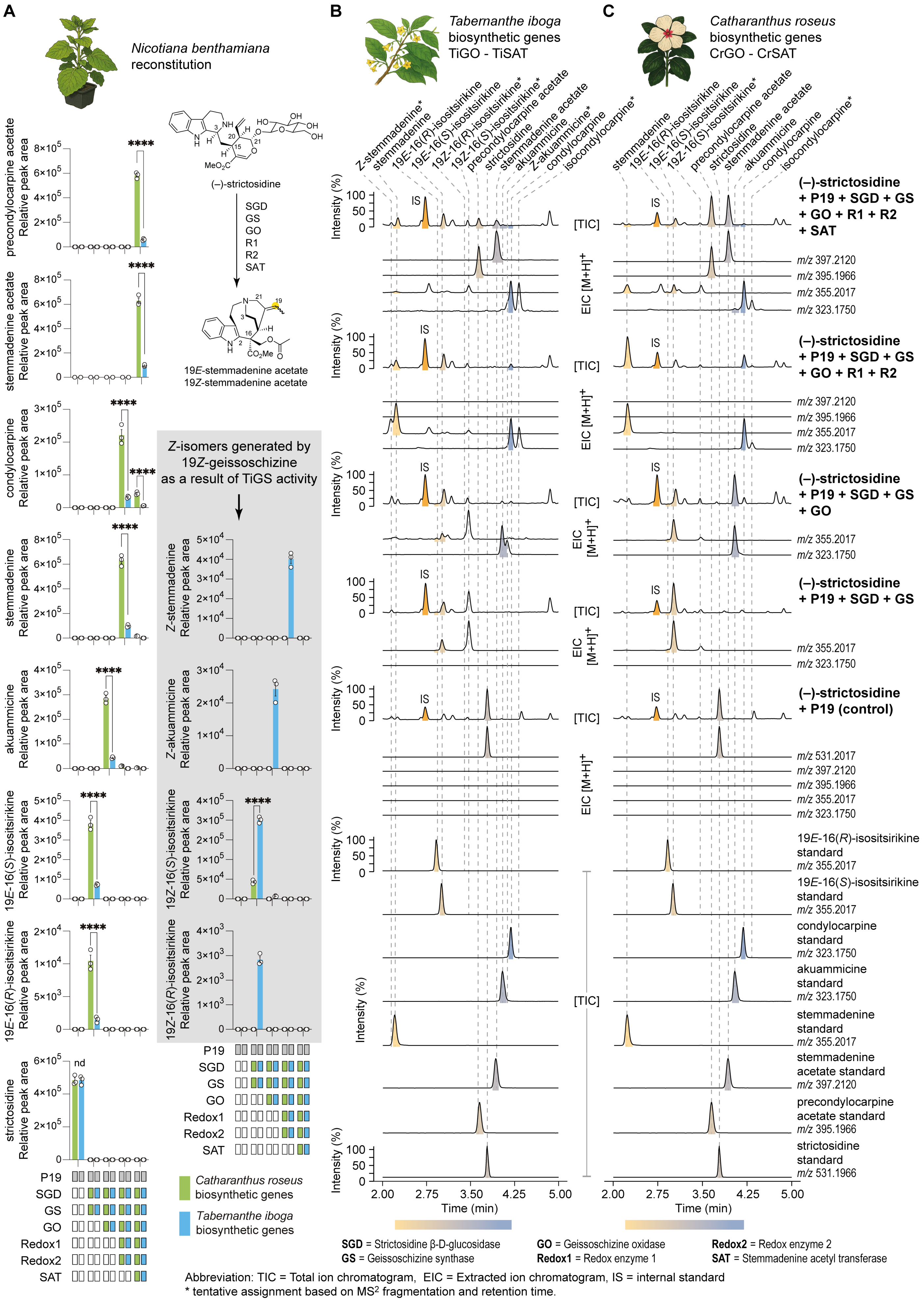
A
Nicotiana benthamiana
reconstitution
(–)-strictosidine
SGD
GS
GO
R1
R2
SAT
19E-stemmadenine acetate
19Z-stemmadenine acetate
precondylocarpine acetate
stemmadenine acetate
condylocarpine
stemmadenine
akuammicine
19E-16(S)-isositsirikine
19E-16(R)-isositsirikine
strictosidine
Relative peak area
Z-isomers generated by
19Z-geissoschizine
as a result of TiGS activity
Z-stemmadenine
Z-akuammicine
19Z-16(S)-isositsirikine
19Z-16(R)-isositsirikine
nd
P19
SGD
GS
GO
Redox1
Redox2
SAT
Catharanthus roseus
biosynthetic genes
Tabernanthe iboga
biosynthetic genes
B
Tabernanthe iboga
biosynthetic genes
TiGO - TiSAT
C
Catharanthus roseus
biosynthetic genes
CrGO - CrSAT
Intensity (%)
IS
[TIC]
EIC [M+H]+
(–)-strictosidine
+ P19 + SGD + GS
+ GO + R1 + R2
+ SAT
(–)-strictosidine
+ P19 + SGD + GS
+ GO + R1 + R2
(–)-strictosidine
+ P19 + SGD + GS
+ GO
(–)-strictosidine
+ P19 + SGD + GS
(–)-strictosidine
+ P19 (control)
m/z 531.2017
m/z 397.2120
m/z 395.1966
m/z 355.2017
m/z 323.1750
19E-16(R)-isositsirikine standard
19E-16(S)-isositsirikine standard
condylocarpine standard
akuammicine standard
stemmadenine standard
stemmadenine acetate standard
precondylocarpine acetate standard
strictosidine standard
m/z 531.1966
Time (min)
SGD = Strictosidine β-D-glucosidase
GS = Geissoschizine synthase
GO = Geissoschizine oxidase
Redox1 = Redox enzyme 1
Redox2 = Redox enzyme 2
SAT = Stemmadenine acetyl transferase
Abbreviation: TIC = Total ion chromatogram, EIC = Extracted ion chromatogram, IS = internal standard
* tentative assignment based on MS2 fragmentation and retention time.

Figure S25. Pathway reconstitution of stemmadenine acetate biosynthesis from strictosidine in *Nicotiana benthamiana*. (**A**) Comparison of metabolite production from pathway reconstitution using previously characterized *Catharanthus roseus* stemmadenine acetate biosynthetic genes (green bars) and orthologous *Tabernanthe iboga* genes described in this study (blue bars) in *N. benthamiana*, with strictosidine as the common starting substrate. Stemmadenine acetate production was lower with *T. iboga* genes, primarily due to the activity of TiGS. Unlike CrGS, which exclusively generates the canonical 19*E*-isomer (19*E*-geissoschizine), TiGS activity also leads to the formation of 19Z-isomeric intermediates and products, highlighted in the grey shaded box. (**B**) Representative LC-MS chromatograms of the pathway intermediates and products generated by *T. iboga* biosynthetic genes expressed in *N. benthamiana*, compared against authentic standards (19*E*-isomers). Analytes were identified by comparison with authentic standards of the 19*E*-configuration. Compounds with a 19*Z*-configuration were annotated based on MS^2 fragmentation patterns and retention time offsets attributable to the *Z*-configuration at C19. (**C**) Representative LC-MS chromatograms of the corresponding pathway intermediates and products from reconstitution with *C. roseus* biosynthetic genes under identical conditions. For all intermediates, data are represented as the mean ± standard deviation (n = 3 biological replicates), based on extracted ion chromatogram peak area ($[M+H]^+$) normalized to the internal standard (IS). Statistical significance was evaluated using an unpaired t-test (****$P < 0.0001$). Bar graphs are plotted as the mean ± SD.

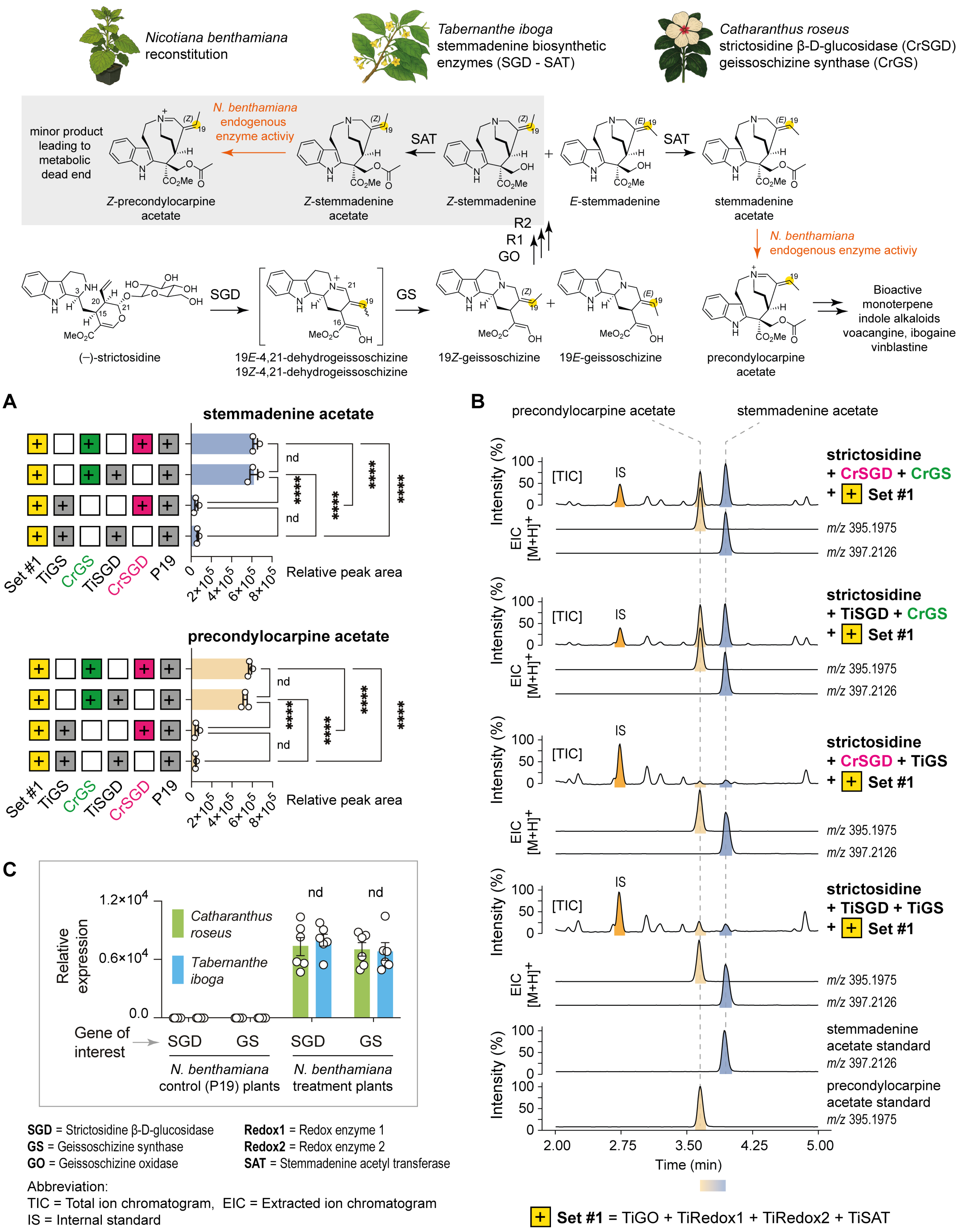

Figure S26. Functional analysis of strictosidine β-D-glucosidase (SGD) and geissoschizine synthase (GS) in the reconstitution of the stemmadenine acetate biosynthetic pathway in *Nicotiana benthamiana*. (**A**) Relative peak areas of stemmadenine acetate and precondylocarpine acetate produced in *N. benthamiana* leaf disks following combinatorial pathway reconstitution of *Tabernanthe iboga* multigene construct (pMGC2) with either *T. iboga* or *Catharanthus roseus* SGD and GS enzymes, using strictosidine as the substrate. Peak areas were normalized to an internal standard and are presented as mean ± standard deviation (n = 3 biological replicates). Statistical analysis was performed using one-way ANOVA (*****P* < 0.0001; nd. = not significant, P > 0.05, n = 3 biological replicates). (**B**) Representative LC-MS chromatograms corresponding to data in panel **A**, comparing product profiles resulting from different combinations of *T. iboga* or *C. roseus* SGD and GS enzymes in pathway reconstitution assays. (**C**) Relative gene expression of *T. iboga* and *C. roseus* SGD and GS genes transiently expressed in *N. benthamiana* leaves for pathway reconstitution. Gene expression was normalized to the reference gene *N. benthamiana* elongation factor 1α (EF1α) and compared to a P19-only control. Data are shown as mean ± standard deviation (n = 6 biological replicates). No significant differences in expression levels were observed between orthologous genes (unpaired *t-test*, nd = not significant P > 0.05). Bar graphs are plotted as the mean ± SD.

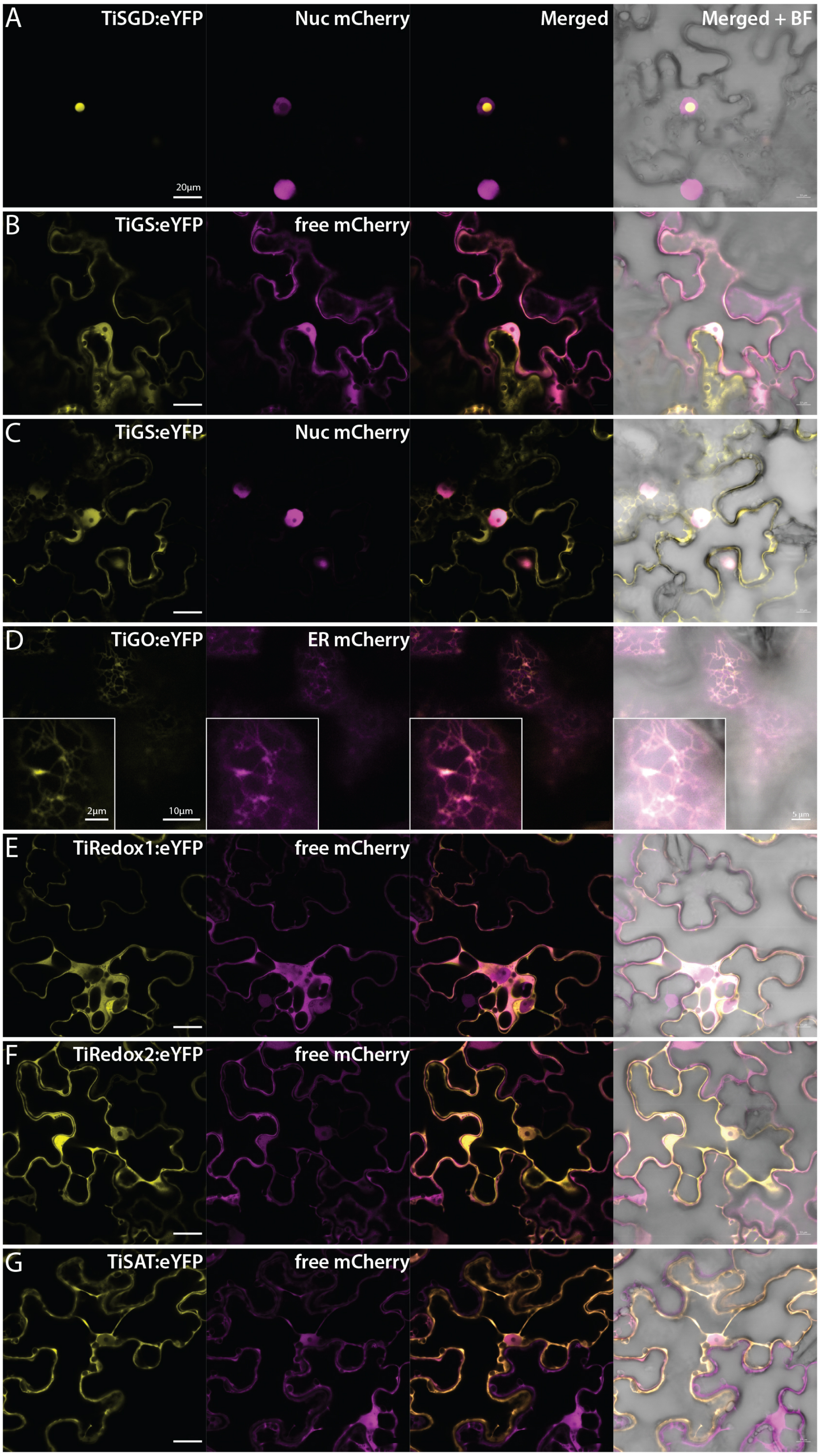

Figure S27. Subcellular localization of *Tabernanthe iboga* stemmadenine acetate biosynthetic enzymes in *Nicotiana benthamiana* leaves. *T. iboga* biosynthetic enzymes were C-terminally fused to enhanced yellow fluorescent protein (eYFP) and transiently co-expressed with subcellular localization marker in *N. benthamiana* leaves. Co-infiltration was performed with subcellular organelle markers fused to mCherry (red fluorescent protein) to determine localization (14). (**A**) *T. iboga* strictosidine β-D-glucosidase localizes to the nucleus. (**B, C**) Geissoschizine synthase (TiGS) localizes both to the nucleus and cytoplasm. (**D**) Geissoschizine oxidase (TiGO) localizes to the endoplasmic reticulum (ER). (**E**) Redox enzyme 1 (TiRedox1) localizes to the cytoplasm. (**F**) Redox enzyme 2 (TiRedox2) localizes to the cytoplasm. (**G**) Stemmadenine acetyltransferase (TiSAT) localizes to the cytoplasm. Scale bars are indicated in white.

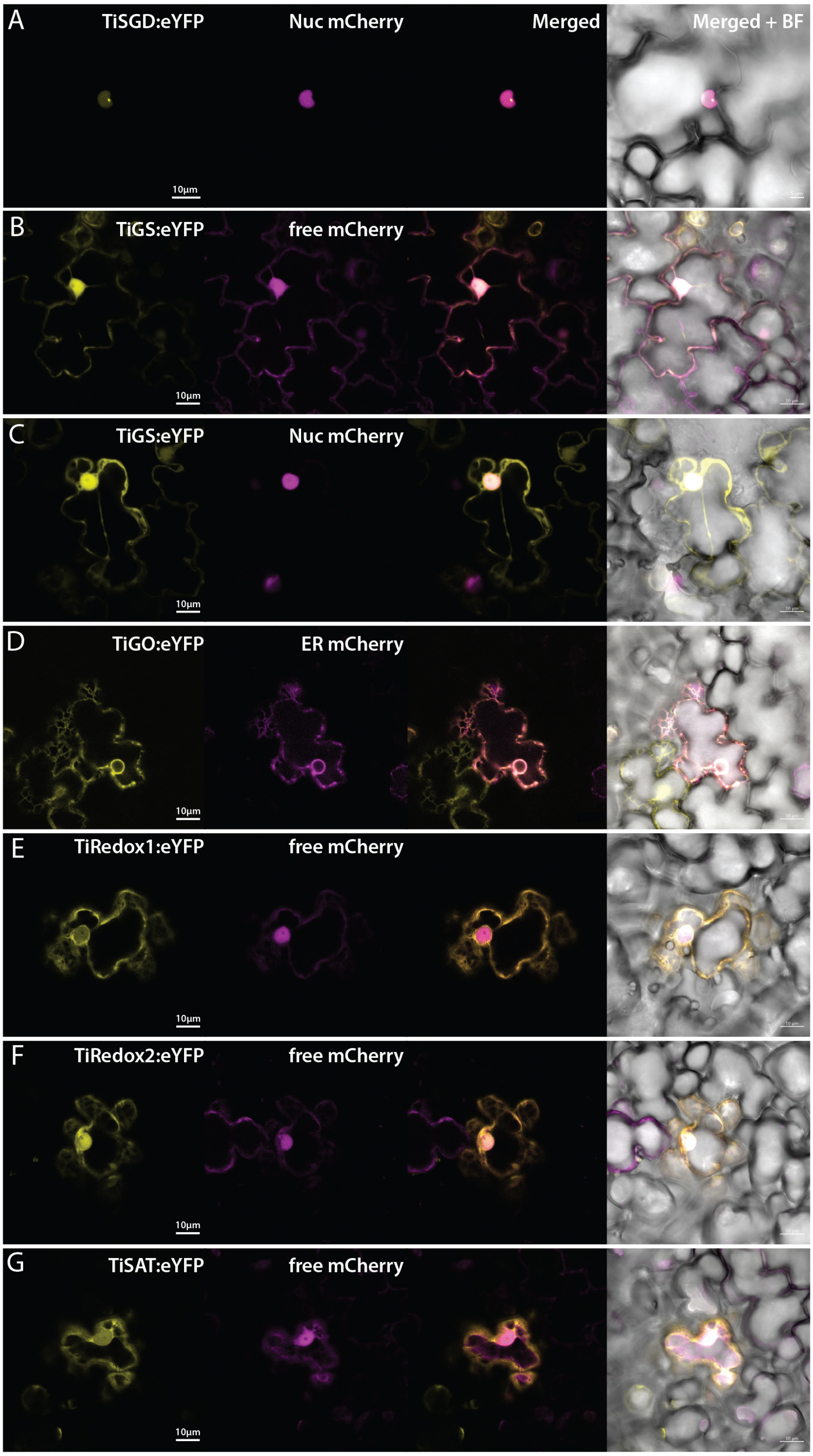

Figure S28. Subcellular localization of *Tabernanthe iboga* stemmadenine acetate biosynthetic enzymes in *Catharanthus roseus* flower petals. *T. iboga* biosynthetic enzymes were C-terminally fused to enhanced yellow fluorescent protein (eYFP) and transiently expressed with subcellular localization marker in *C. roseus* flower petals. Co-infiltration was performed with subcellular organelle markers fused to mCherry (red fluorescent protein) to determine localization (14). (**A**) *T. iboga* strictosidine β-D-glucosidase localizes to the nucleus. (**B, C**) Geissoschizine synthase (TiGS) localizes both to the nucleus and cytoplasm. (**D**) Geissoschizine oxidase (TiGO) localizes to the endoplasmic reticulum (ER). (**E**) Redox enzyme 1 (TiRedox1) localizes to the cytoplasm. (**F**) Redox enzyme 2 (TiRedox2) localizes to the cytoplasm. (**G**) Stemmadenine acetyltransferase (TiSAT) localizes to the cytoplasm. Scale bars are indicated in white.

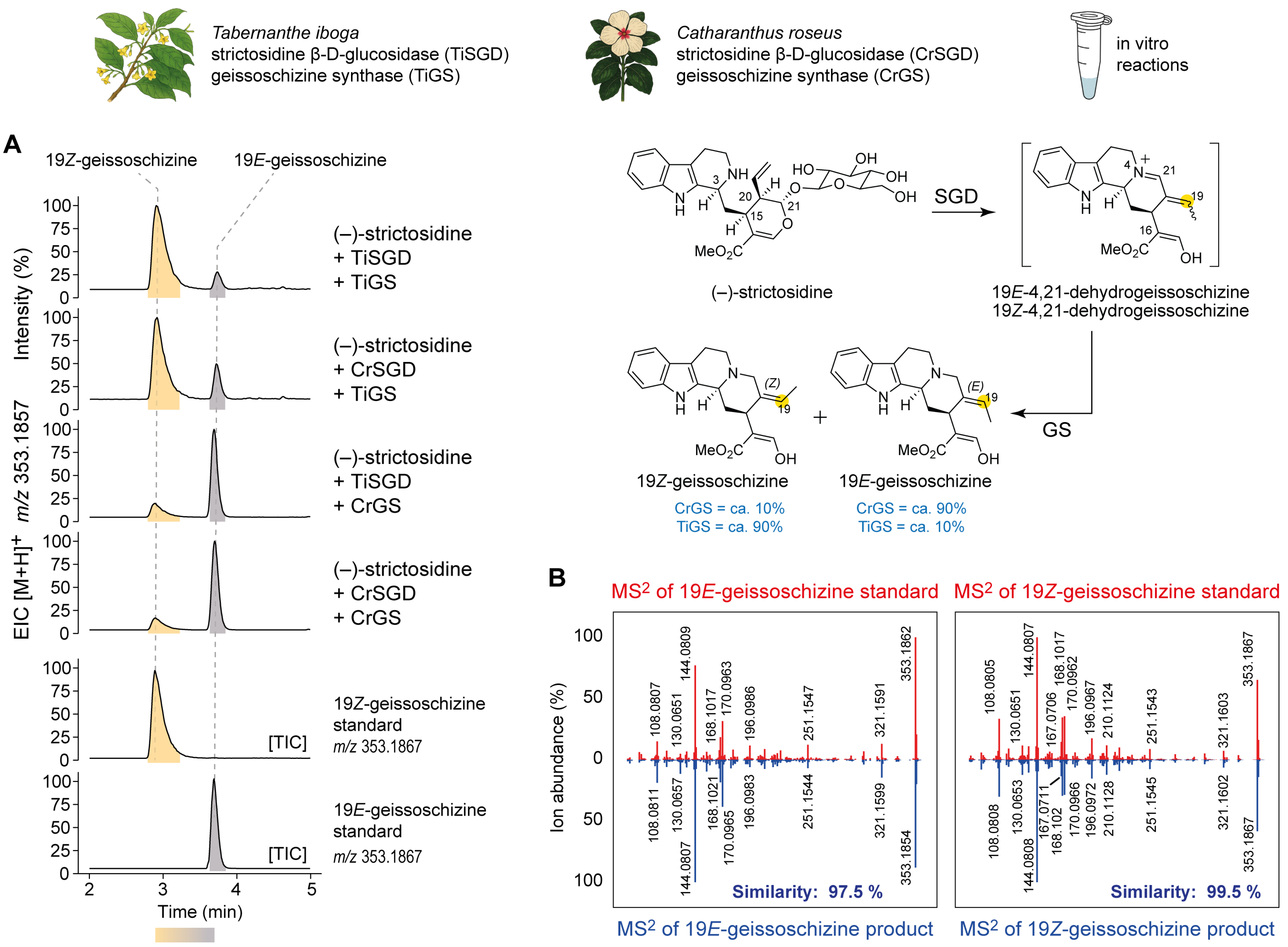

Figure S29. Functional characterization of *Tabernanthe iboga* geissoschizine synthase (GS) enzyme activity. (**A**) LC-MS chromatograms of in vitro enzymatic assays using heterologously expressed and purified GS and strictosidine β-D-glucosidase (SGD) enzymes from *T. iboga* and *C. roseus*, assayed with the substrate strictosidine. Coupled enzymatic assays revealed that *T. iboga* GS predominantly produces the 19*Z*-geissoschizine isomer, whereas *C. roseus* GS predominantly yields the 19*E*-geissoschizine isomer. (**B**) MS^2 spectra of the 19*E*- and 19*Z*-geissoschizine isomers generated by SGD-GS coupled reactions, shown alongside spectra of authentic synthetic standards.

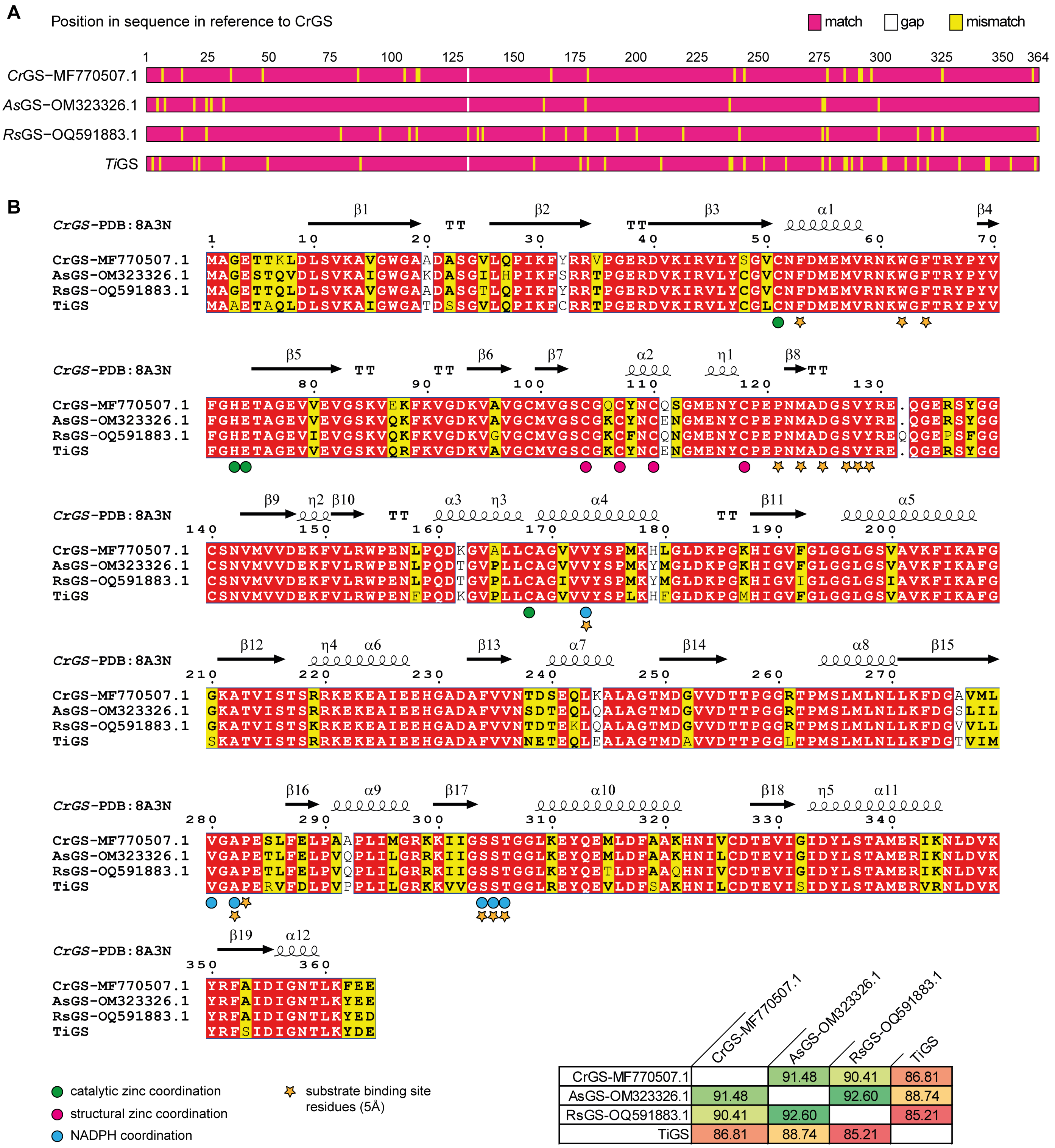

	CrGS-MF770507.1	AsGS-OM323326.1	RsGS-OQ591883.1	TiGS
CrGS-MF770507.1		91.48	90.41	86.81
AsGS-OM323326.1	91.48		92.60	88.74
RsGS-OQ591883.1	90.41	92.60		85.21
TiGS	86.81	88.74	85.21	

Figure S30. Multiple sequence alignment of geissoschizine synthase (GS) from monoterpene indole alkaloid (MIA) producing plant species. (**A**) Schematic representation of amino acid sequence alignment of functionally characterized GS enzymes, members of the medium-chain alcohol dehydrogenase/reductase (MDR) family (15), from various MIA-producing plant species, including *T. iboga* GS. (**B**) Detailed alignment of GS enzyme sequences, annotated with structural features based on *C. roseus* GS crystal structure (16). Key residues involved in zinc coordination (structural and catalytic), NADPH binding, and substrate pocket binding interactions are highlighted (15, 16). The percentage amino acid identity between TiGS and GS enzymes from other plant species is also shown. GenBank accession numbers of the functionally characterized GS enzymes are indicated as appropriate.

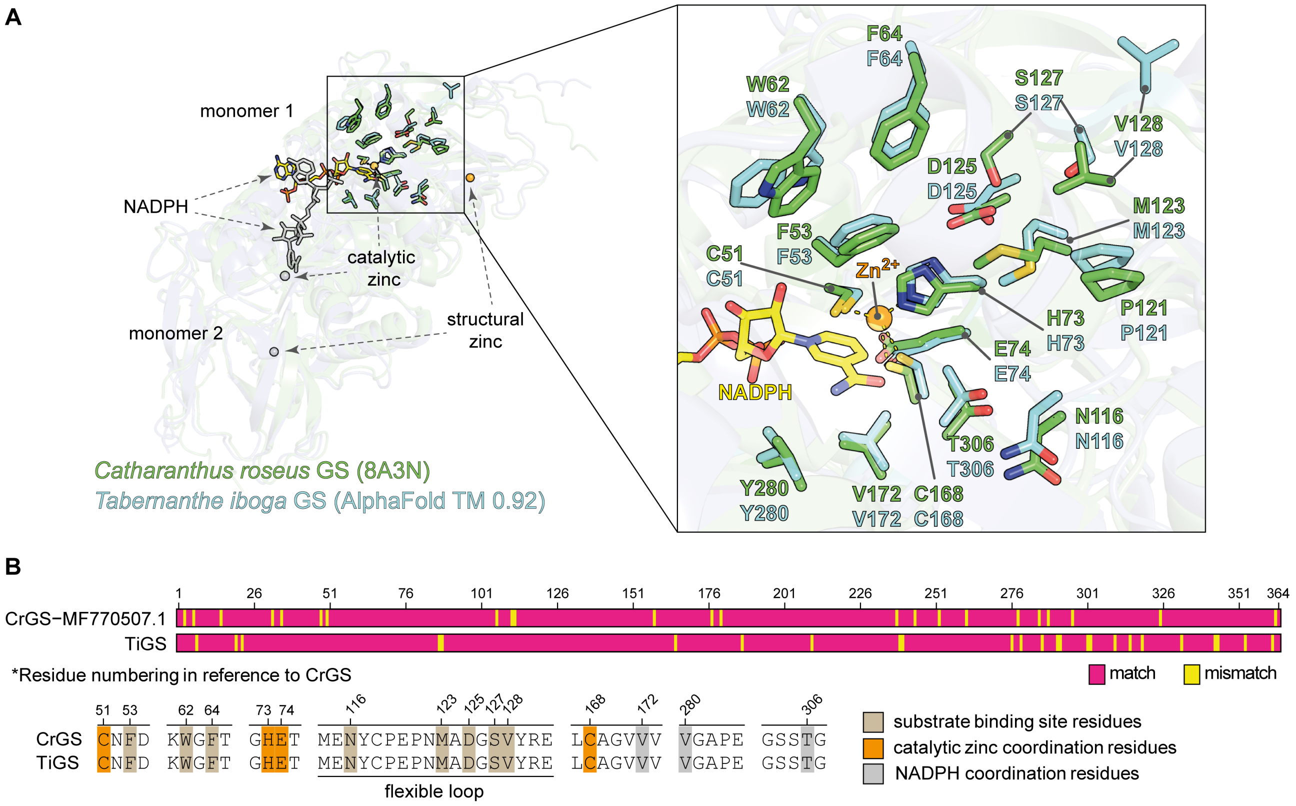

Figure S31. Structural analysis of *Tabernanthe iboga* geissoschizine synthase (TiGS) model. (**A**) Superimposed structures of the AlphaFold v3 model of TiGS (TM-score = 0.9261) and the experimentally determined crystal structure of *Catharanthus roseus* geissoschizine synthase (CrGS; PDB 8A3N) (16). The two medium-chain dehydrogenases/reductase (MDR) enzymes share a high structural similarity (RMSD 0.8), with the overall architecture of the dimeric protomers, the active site, catalytic and structural zinc binding sites, as well as NADPH binding being conserved. (**B**) Schematic alignment of TiGS and CrGS amino acid sequences (86.81% identity), highlighting conserved and divergent regions. Residues involved in catalytic zinc coordination (Cys51, His73, Glu74, and Cys168), NADPH coordination, and putative substrate-binding residues are indicated (16). No significant variation was observed in the catalytic pocket residues between TiGS and CrGS.

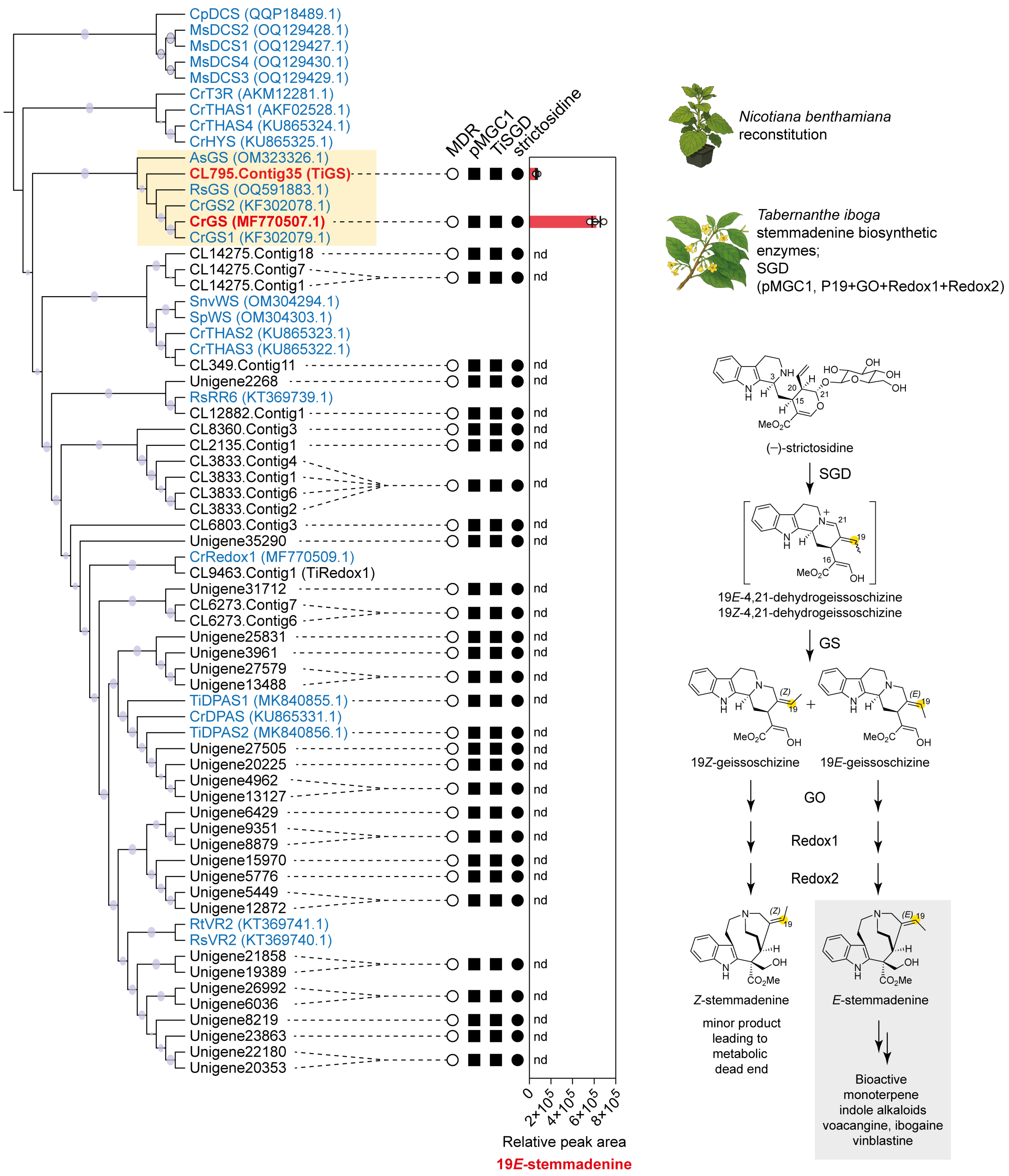

Figure S32. Screening *Tabernanthe iboga* MDRs for geissoschizine synthase-like activity. Medium chain dehydrogenase/reductase (MDR) candidates implicated in iminium reduction were tested for geissoschizine synthase (GS) activity by transient expression in *N. benthamiana*. Genes were selected based on homology to GS and whether these genes were expressed in *T. iboga* leaf. Co-expression of multigene construct pMGC1 harbouring P19, TiGO, TiRedox1, and TiRedox2, with TiSGD and a single MDR candidate, followed by strictosidine (substrate) feeding. GS activity was assessed by LC-MS detection of stemmadenine, where CrGS served as a positive control. Bar graphs represent peak areas normalized to an internal standard (mean ± standard deviation, *n* = 3 biological replicates).

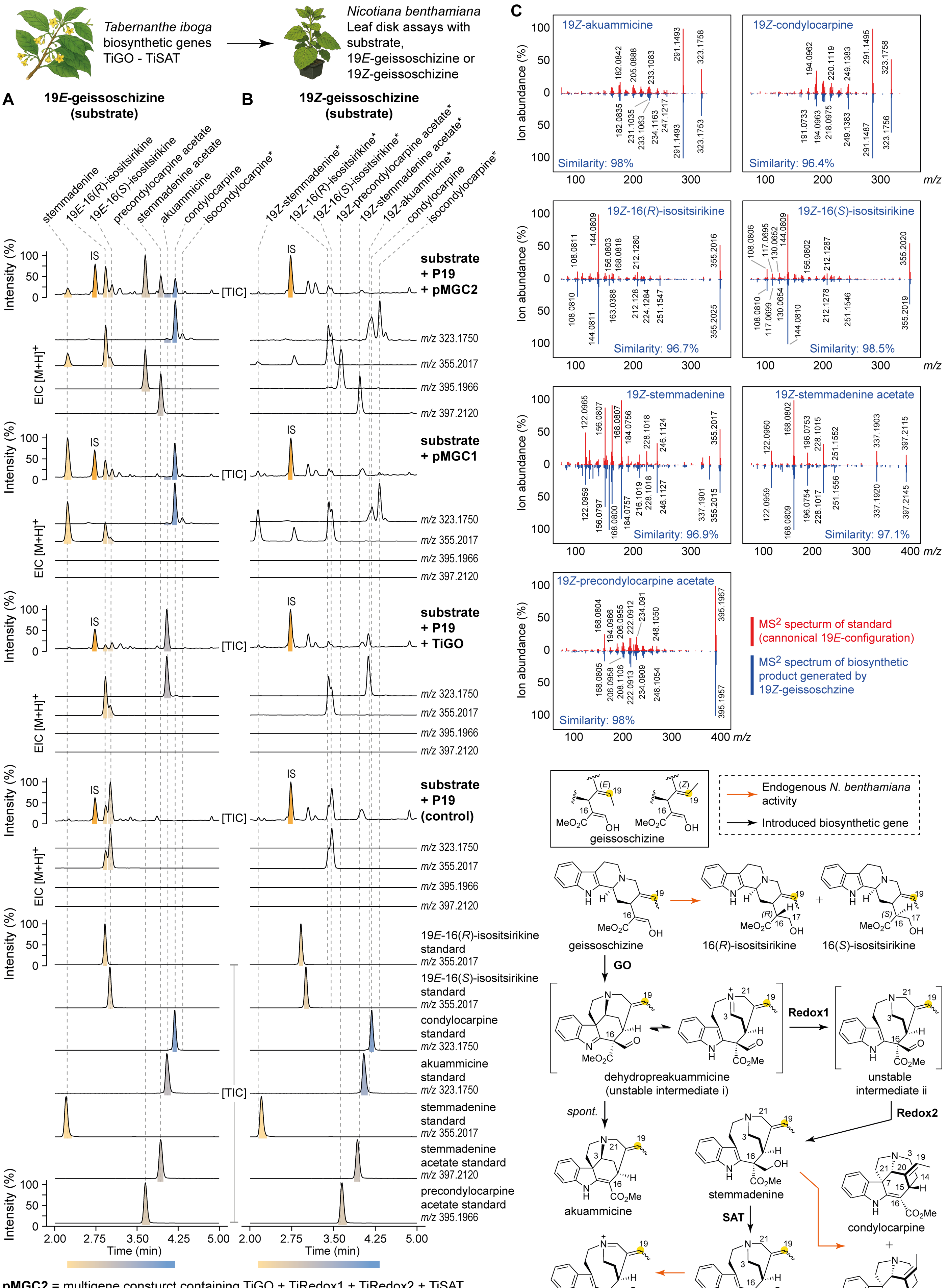

pMGC2 = multigene consturct containing TiGO + TiRedox1 + TiRedox2 + TiSAT
pMGC1 = multigene consturct containing TiGO + TiRedox1 + TiRedox2 + P19

Abbreviation: TIC = Total ion chromatogram, EIC = Extracted ion chromatogram, IS = internal standard
* tentative assignment based on MS^2 fragmentation and retention time.

Figure S33. Pathway reconstitution of *Tabernanthe iboga* stemmadenine acetate biosynthetic genes in *Nicotiana benthamiana* and biosynthetic outcomes following 19*E*- and 19*Z*-geissoschizine substrate feeding. Pathway reconstitution in *N. benthamiana* was performed by transient expression of multigene constructs encoding *T. iboga* stemmadenine acetate biosynthetic enzymes, followed by substrate feeding with 19*E*- or 19*Z*-geissoschizine. (**A**) LC-MS chromatograms of biosynthetic products obtained from 19*E*-geissoschizine feeding. Canonical products of the pathway, including stemmadenine acetate and its biosynthetic intermediates, were detected. (**B**) LC-MS chromatograms of products generated upon 19Z-geissoschizine feeding. 19Z-isomeric analogs of stemmadenine acetate pathway intermediates and products were detected, albeit at lower levels. (**C**) Comparison of MS^2 fragmentation spectra of representative 19*E*- and 19*Z*-isomeric products. MS^2 spectra of 19*Z*-isomers show high similarity to those of their 19*E* counterparts, supporting structural assignment. Reaction products were annotated by comparison to authentic 19*E*-isomer standards based on diagnostic MS^2 fragmentation and expected retention time offsets arising from the 19Z-configuration at C19.

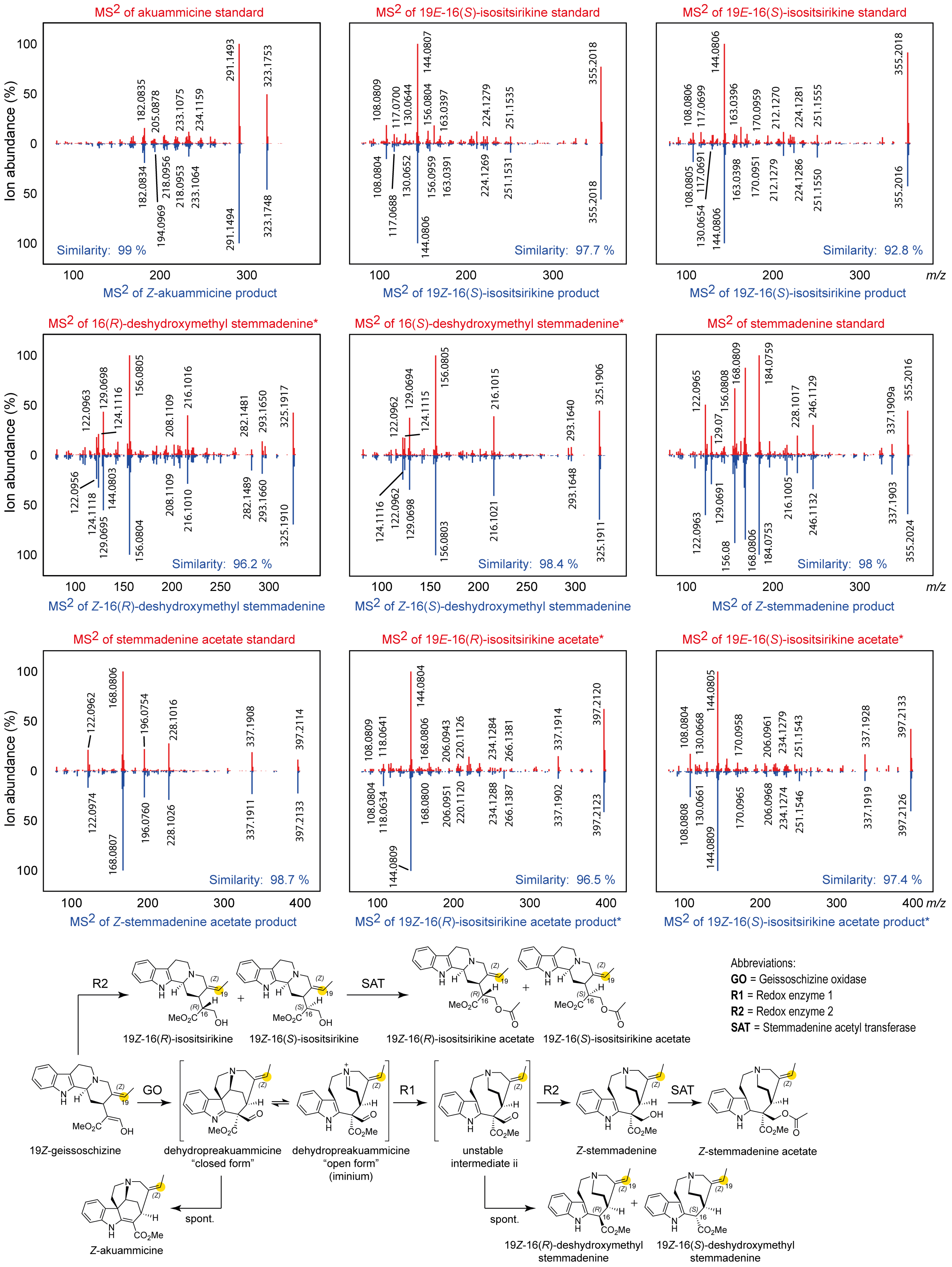

Figure S34. MS^2 spectra of 19*Z*-isomers generated by the 19Z-geissoschizine. MS^2 fragmentation spectra of 19*Z*-isomeric products generated from 19*Z*-geissoschizine following in vitro cascade reactions of *T. iboga* stemmadenine acetate biosynthetic genes, compared with MS^2 spectra of the corresponding 19*E*-isomer authentic standards produced by the canonical *Catharanthus roseus* MIA biosynthetic pathway.

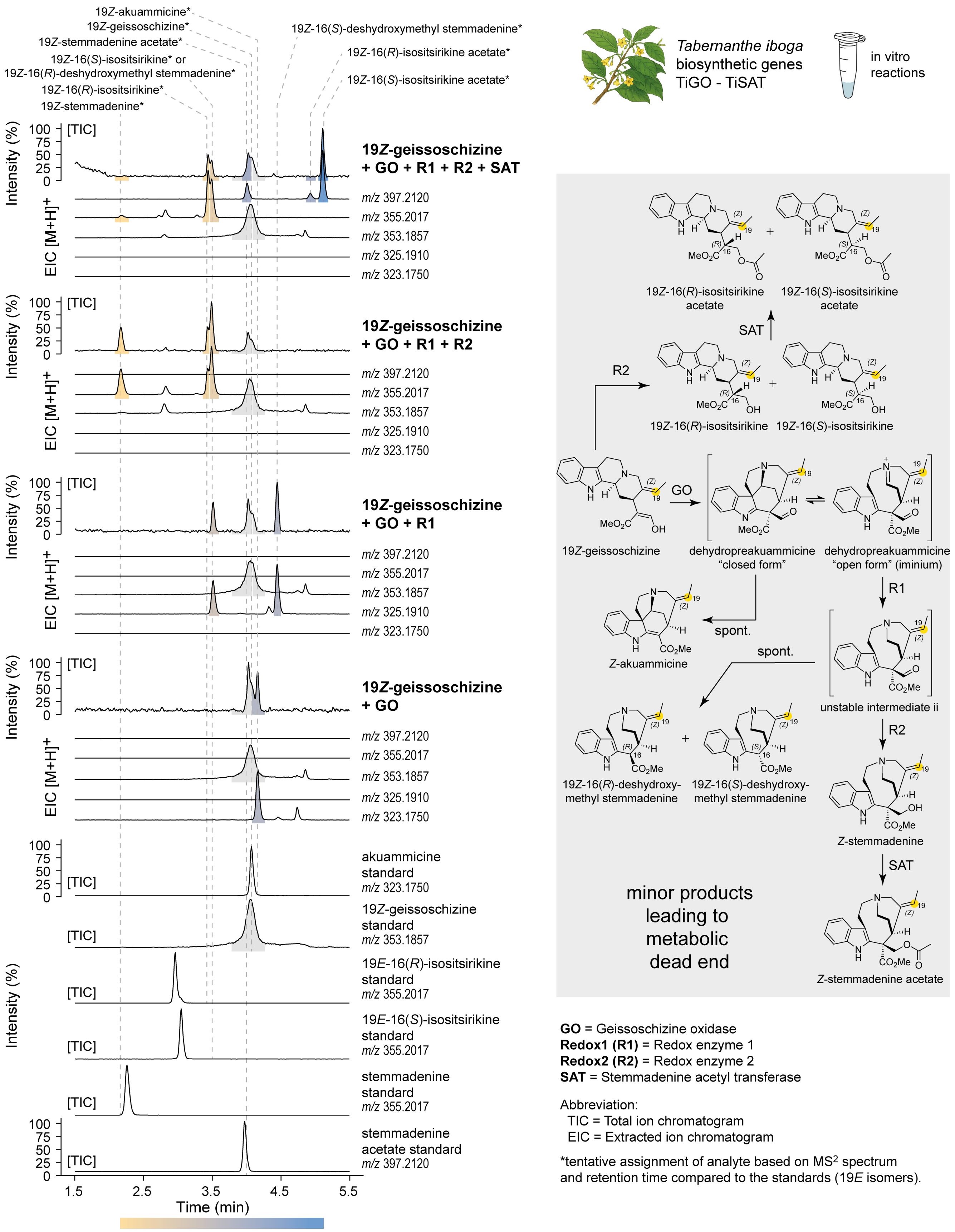

GO = Geissoschizine oxidase
Redox1 (R1) = Redox enzyme 1
Redox2 (R2) = Redox enzyme 2
SAT = Stemmadenine acetyl transferase

Abbreviation:
TIC = Total ion chromatogram
EIC = Extracted ion chromatogram

*tentative assignment of analyte based on MS^2 spectrum and retention time compared to the standards (19*E* isomers).

Figure S35. Stepwise in vitro reconstitution of 19*Z*-isomeric stemmadenine acetate pathway intermediates from 19*Z*-geissoschizine using *Tabernanthe iboga* biosynthetic enzymes. LC-MS chromatograms showing the sequential enzymatic conversion of 19*Z*-geissoschizine to 19*Z*-stemmadenine acetate via the action of *T. iboga* GO, Redox 1, Redox2, and SAT. The 19*Z*-isomeric products were produced at low levels, as evidenced by TIC and EIC traces, and are not biosynthetic precursors of bioactive compounds ibogaine and voacangine in *T. iboga*. Enzymes were incubated in combination to demonstrate stepwise pathway progression from 19*Z*-geissoschizine substrate. Reaction products were annotated by comparison to authentic 19*E*-isomer standards based on MS^2 fragmentation and retention time offsets attributable to the 19Z-configuration at C19.

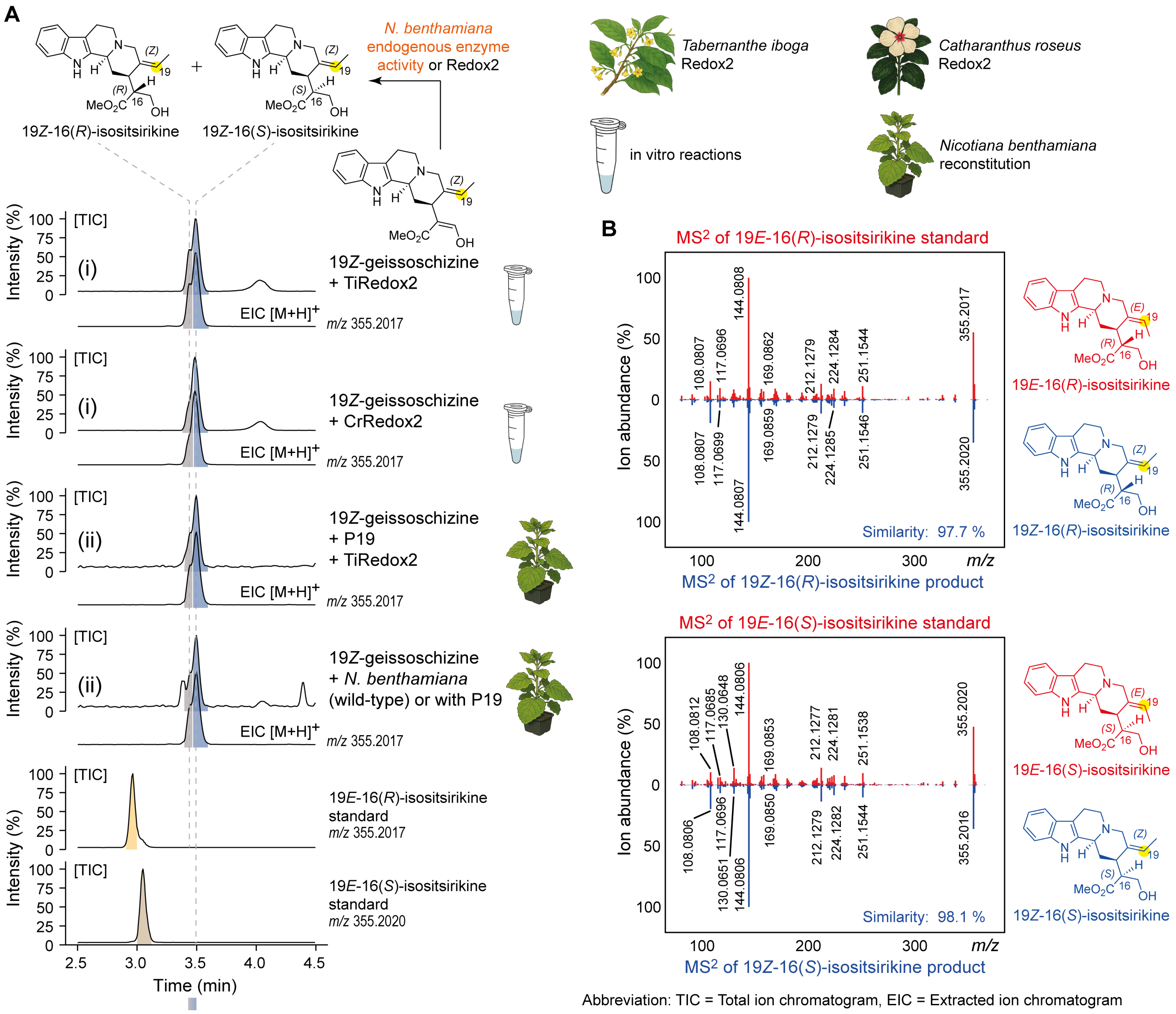

Figure S36. Functional characterization of *Tabernanthe iboga* redox enzyme 2 (TiRedox2) activity using 19Z-geissoschizine. (A) (i) LC-MS chromatograms of in vitro enzymatic assays using heterologously expressed TiRedox2 and *Catharanthus roseus* (CrRedox2), assayed with 19*Z*-geissoschizine as the substrate. Both enzymes catalyzed the reduction of 19*Z*-geissoschizine to the isomeric products 19*Z*-16(*R*)-isositsirikine and 19*Z*-16(*S*)-isositsirikine. (ii) LC-MS chromatograms showing that endogenous *Nicotiana benthamiana* metabolic activity also reduces 19*Z*-geissoschizine to the identical 19*Z*-isositsirikine isomers. (**B**) MS^2 spectra of the 19*Z*-16(*R*)-isositsirikine and 19*Z*-16(*S*)-isositsirikine isomers produced by TiRedox2 exhibited high spectral similarity to the authentic 19*E*-isomer standards, supporting the tentative structural assignment of the 19*Z*-isomers.

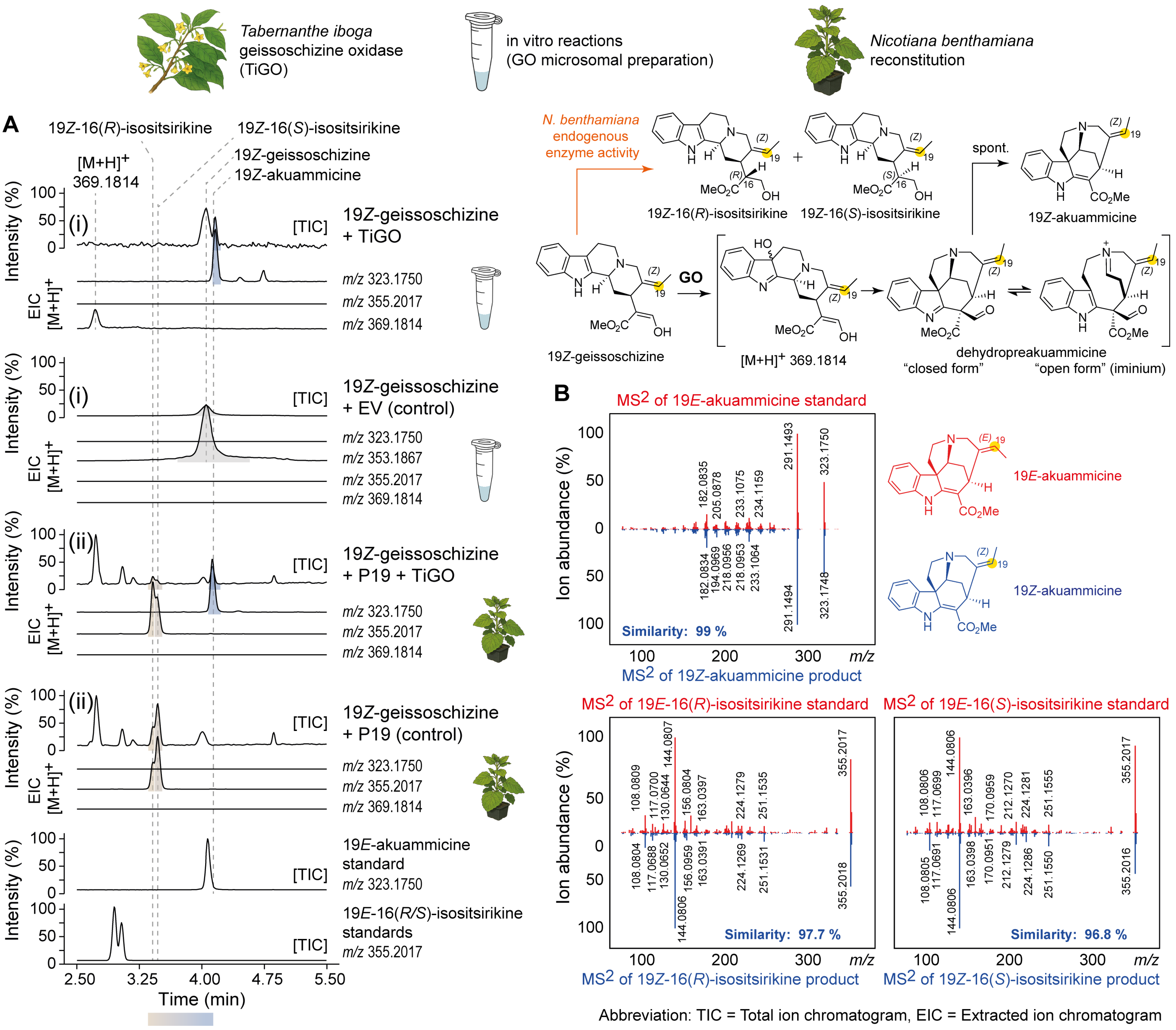

Figure S37. Functional characterization of *Tabernanthe iboga* geissoschizine oxidase (TiGO) activity with 19*Z*-geissoschizine. (**A**) (i) LC-MS chromatograms of in vitro enzymatic assays using yeast microsomes expressing TiGO, incubated with 19*Z*-geissoschizine as substrate. (ii) LC-MS chromatograms of TiGO transiently expressed in *Nicotiana benthamiana* with the P19 viral silencing suppressor, assayed in leaf discs supplied with 19*Z*-geissoschizine. In both systems, TiGO catalyzed the oxidation of 19*Z*-geissoschizine to produce the thermodynamic product 19*Z*-akuammicine in the absence of downstream biosynthetic enzymes. The endogenous *N. benthamiana* metabolic activity resulted in the reduction of 19*Z*-geissoschizine to 19*Z*-isositsirikine isomers. (**B**) MS^2 spectrum of TiGO-generated 19*Z*-configured products from both in vivo and in vitro assays, compared with spectra of authentic 19*E*-configured standards.

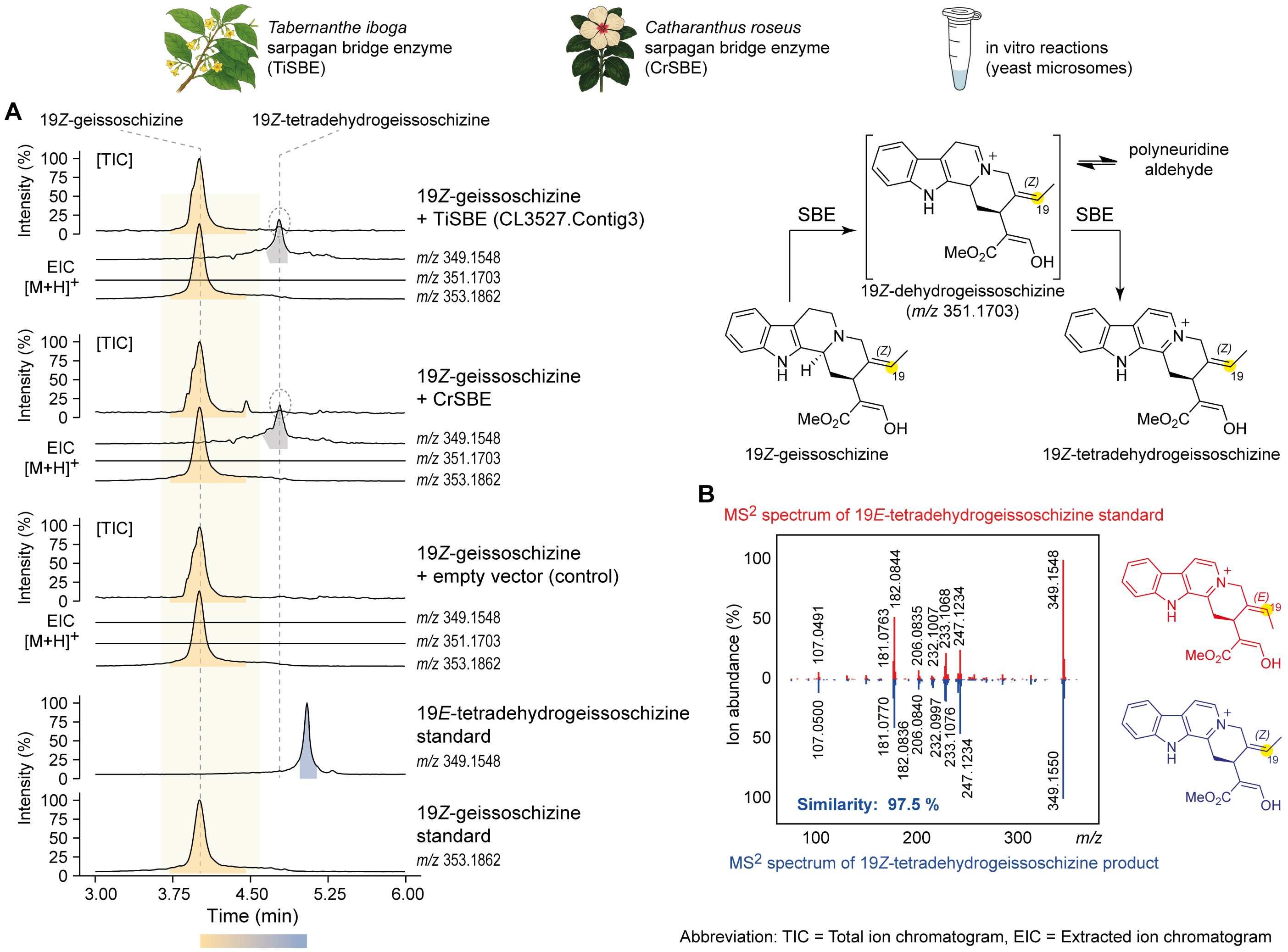

Figure S38. Functional characterization of *Tabernanthe iboga* sarpagan bridge enzyme (TiSBE) activity in vitro using 19*Z*-geissoschizine. (**A**) LC-MS chromatograms of in vitro enzymatic assays using yeast microsomes expressing TiSBE or *Catharanthus roseus* SBE (CrSBE). Microsomal fractions were assayed with 19*Z*-geissoschizine as substrate under identical conditions. Both enzymes catalyzed the formation of 19*Z*-tetradehydrogeissoschizine in very low amounts. (**B**) MS^2 spectrum of 19*Z*-tetradehydrogeissoschizine produced by TiSBE, compared with that of the authentic standard of the 19*E*-variant.

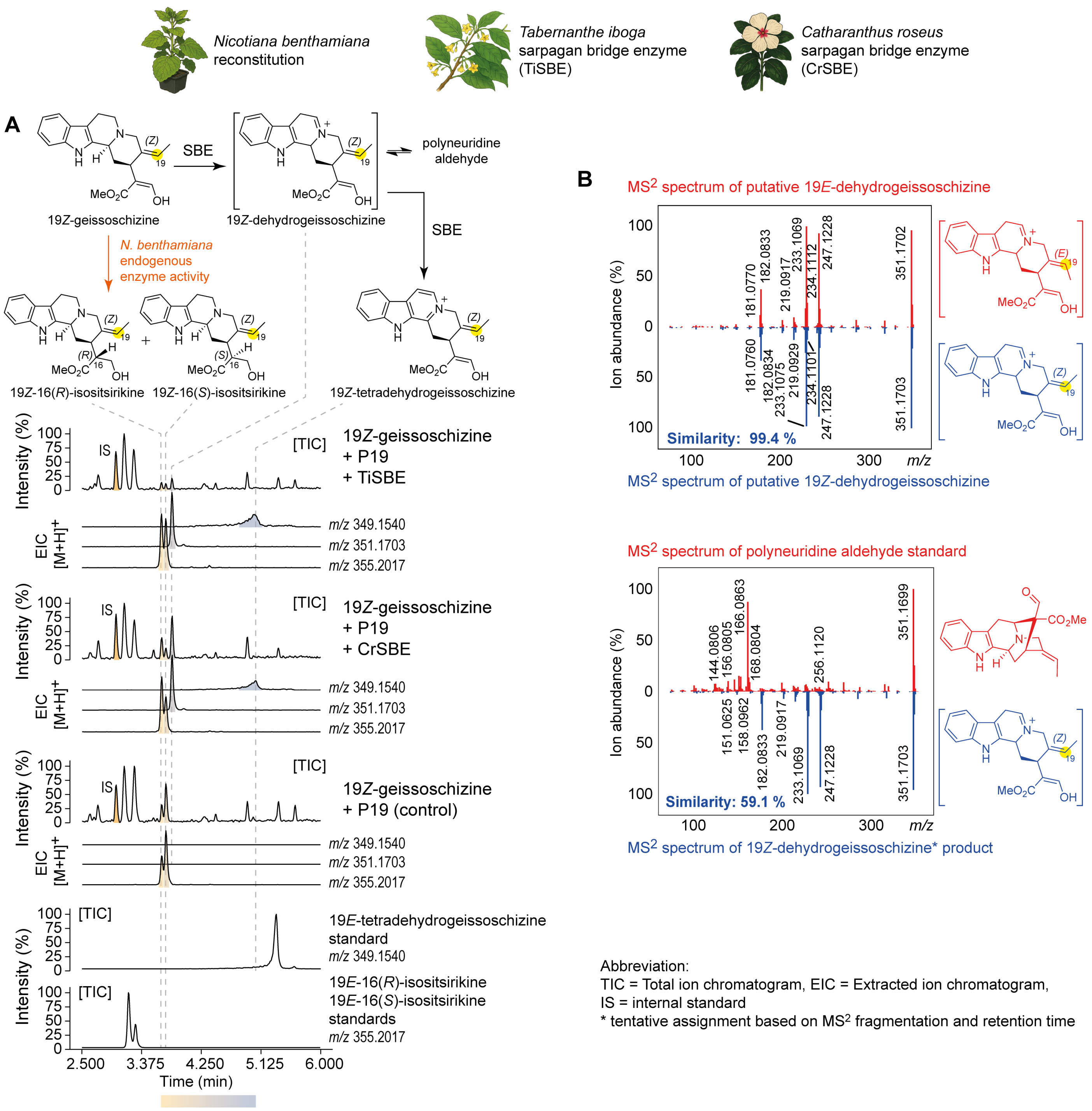

Figure S39. Functional characterization of *Tabernanthe iboga* sarpagan bridge enzyme (TiSBE) activity in *Nicotiana benthamiana* using 19Z-geissoschizine as substrate. (**A**) LC-MS chromatograms of TiSBE transiently expressed in *N. benthamiana* with the P19 viral silencing suppressor, assayed in leaf disks supplied with the substrate 19*Z*-geissoschizine. The previously characterized *Catharanthus roseus* SBE (CrSBE) (5) was included as a positive control under identical conditions. Both CrSBE and TiSBE catalyzed the formation of 19*Z*-tetradehydrogeissoschizine via the unstable intermediate 19*Z*-dehydrogeissoschizine, albeit at low abundance. Notably, the endogenous metabolic activity of *N. benthamiana* resulted in the reduction of 19*Z*-geissoschizine to 19*Z*-isositsirikine isomers. These 19Z-isomers were annotated based on MS^2 spectral similarity and retention time comparisons to the corresponding 19*E*-structural variants, for which authentic standards were available. (**B**) The MS^2 spectrum of the 19Z-dehydrogeissoschizine showed high similarity to that of 19*E*-dehydrogeissoschizine. This spectrum also differed from polyneuridine aldehyde, which could exist in equilibrium with 19*Z*-dehydrogeissoschizine, further supporting its structural annotation.

References

1. Farrow, S. C., Kamileen, M. O., Caputi, L., Bussey, K., Mundy, J. E. A., McAtee, R. C., Stephenson, C. R. J., and O'Connor, S. E. (2019) Biosynthesis of an Anti-Addiction Agent from the Iboga Plant. *J Am Chem Soc*. **141**, 12979–12983

2. Kamileen, M. O., Nakamura, Y., Luck, K., Heinicke, S., Hong, B., Colinas, M., Lichman, B. R., and O'Connor, S. E. (2024) Streamlined screening platforms lead to the discovery of pachysiphine synthase from Tabernanthe iboga. *N. Phytol.* 10.1111/nph.20133

3. Qu, Y., Easson, M. E. A. M., Simionescu, R., Hajicek, J., Thamm, A. M. K., Salim, V., and Luca, V. D. (2018) Solution of the multistep pathway for assembly of corynanthean, strychnos, iboga, and aspidosperma monoterpenoid indole alkaloids from 19E-geissoschizine. *Proc. Natl. Acad. Sci. United States Am.* **115**, 3180–3185

4. Caputi, L., Franke, J., Farrow, S. C., Chung, K., Payne, R. M. E., Nguyen, T.-D., Dang, T.-T. T., Carqueijeiro, I. S. T., Koudounas, K., Bernonville, T. D. de, Ameyaw, B., Jones, D. M., Vieira, I. J. C., Courdavault, V., and O'Connor, S. E. (2018) Missing enzymes in the biosynthesis of the anticancer drug vinblastine in Madagascar periwinkle. *Science*. **360**, 1235–1239

5. Kamileen, M. O., Hong, B., Gase, K., Kunert, M., Caputi, L., Lichman, B. R., and O'Connor, S. E. (2025) Oxidative Rearrangements of the Alkaloid Intermediate Geissoschizine. *Angew. Chem. Int. Ed.* **64**, e202501323

6. Wang, Z., Xiao, Y., Wu, S., Chen, J., Li, A., and Tatsis, E. C. (2022) Deciphering and reprogramming the cyclization regioselectivity in bifurcation of indole alkaloid biosynthesis. *Chem. Sci.* **13**, 12389–12395

7. Carr, S. C., Torres, M. A., Morris, J. S., Facchini, P. J., and Ng, K. K. S. (2021) Structural studies of codeinone reductase reveal novel insights into aldo-keto reductase function in benzylisoquinoline alkaloid biosynthesis. *J. Biol. Chem.* **297**, 101211

8. JEZ, J. M., BENNETT, M. J., SCHLEGEL, B. P., LEWIS, M., and PENNING, T. M. (1997) Comparative anatomy of the aldo–keto reductase superfamily. *Biochem. J.* **326**, 625–636

9. Abramson, J., Adler, J., Dunger, J., Evans, R., Green, T., Pritzel, A., Ronneberger, O., Willmore, L., Ballard, A. J., Bambrick, J., Bodenstein, S. W., Evans, D. A., Hung, C.-C., O'Neill, M., Reiman, D., Tunyasuvunakool, K., Wu, Z., Žemgulytė, A., Arvaniti, E., Beattie, C., Bertolli, O., Bridgland, A., Cherepanov, A., Congreve, M., Cowen-Rivers, A. I., Cowie, A., Figurnov, M., Fuchs, F. B., Gladman, H., Jain, R., Khan, Y. A., Low, C. M. R., Perlin, K., Potapenko, A., Savy, P., Singh, S., Stecula, A., Thillaisundaram, A., Tong, C., Yakneen, S., Zhong, E. D., Zielinski, M., Žídek, A., Bapst, V., Kohli, P., Jaderberg, M., Hassabis, D., and Jumper, J. M. (2024) Accurate structure prediction of biomolecular interactions with AlphaFold 3. *Nature*. **630**, 493–500

10. Eberhardt, J., Santos-Martins, D., Tillack, A. F., and Forli, S. (2021) AutoDock Vina 1.2.0: New Docking Methods, Expanded Force Field, and Python Bindings. *J. Chem. Inf. Model.* **61**, 3891–3898

11. Schlegel, B. P., Jez, J. M., and Penning, T. M. (1998) Mutagenesis of 3α-Hydroxysteroid Dehydrogenase Reveals a "Push−Pull" Mechanism for Proton Transfer in Aldo−Keto Reductases †. *Biochemistry*. **37**, 3538–3548

12. Penning, T. M., Wangtrakuldee, P., and Auchus, R. J. (2018) Structural and Functional Biology of Aldo-Keto Reductase Steroid-Transforming Enzymes. *Endocr. Rev.* **40**, 447–475

13. Tjallinks, G., Mattevi, A., and Fraaije, M. W. (2024) Biosynthetic Strategies of Berberine Bridge Enzyme-like Flavoprotein Oxidases toward Structural Diversification in Natural Product Biosynthesis. *Biochemistry*. **63**, 2089–2110

14. Ivanov, S., and Harrison, M. J. (2014) A set of fluorescent protein-based markers expressed from constitutive and arbuscular mycorrhiza-inducible promoters to label organelles, membranes and cytoskeletal elements in Medicago truncatula. *Plant J.* **80**, 1151–1163

15. Carr, S. C., and O'Connor, S. E. (2025) A Tight-Knit Family: The Medium-Chain Dehydrogenase/Reductases of Monoterpene Indole Alkaloid Biosynthesis. *Biochemistry*. **64**, 2712–2726

16. Langley, C., Tatsis, E., Hong, B., Nakamura, Y., Paetz, C., Stevenson, C. E. M., Basquin, J., Lawson, D. M., Caputi, L., and O'Connor, S. E. (2022) Expansion of the Catalytic Repertoire of Alcohol Dehydrogenases in Plant Metabolism**. *Angew. Chem. Int. Ed.* **61**, e202210934
